# Asymmetric Conjugate Addition of α,α-Disubstituted Aldehydes to Nitroalkenes Organocatalyzed by Chiral Monosalicylamides from *trans*-Cyclohexane-1,2-Diamines

**DOI:** 10.3390/molecules23010141

**Published:** 2018-01-11

**Authors:** José R. Martínez-Guillén, Jesús Flores-Ferrándiz, Cecilia Gómez, Enrique Gómez-Bengoa, Rafael Chinchilla

**Affiliations:** 1 Department of Organic Chemistry, Faculty of Sciences, and Institute of Organic Synthesis (ISO), University of Alicante, P.O. Box 99, 03080 Alicante, Spain; jramonimg@gmail.com (J.R.M.-G.); jfloresf@gmail.com (J.F.-F.); cgomez@ua.es (C.G.); 2 Department of Organic Chemistry I, University of the Basque Country, P.O. Box 1072, 20080 San Sebastíán, Spain

**Keywords:** organocatalysis, asymmetric synthesis, Michael addition, nitroalkenes

## Abstract

Primary amine-salicylamides derived from chiral *trans*-cyclohexane-1,2-diamines are used as organocatalysts for the enantioselective conjugate addition of α,α-disubstituted aldehydes to arylated and heteroarylated nitroalkenes. The reaction is performed in the presence of 4-dimethylaminopyridine as an additive in dichloromethane as a solvent at room temperature. The corresponding enantioenriched γ-nitroaldehydes are obtained with enantioselectivities up to 95%. Theoretical calculations are used to justify the reasons of the stereoinduction.

## 1. Introduction

The asymmetric synthesis of γ-nitroaldehydes has gained great importance during recent years. They are precursors of γ-aminobutyric acid analogues (GABAs), which exhibit a range of pharmacological activities including antidepressant, anticonvulsant, anxiolytic and others [1,2]. In addition, GABA derivatives can be potent drugs in the treatment of neurodegenerative disorders [3]. Moreover, the presence of the versatile nitro group allows for further transformations to valuable compounds. This is largely due to the utility of the nitro group as a masked functionality to be transformed to a variety of other useful functional groups [4,5], which was well described by Seebach with the term ‘synthetic chameleon’ [6].

**Figure 1:**
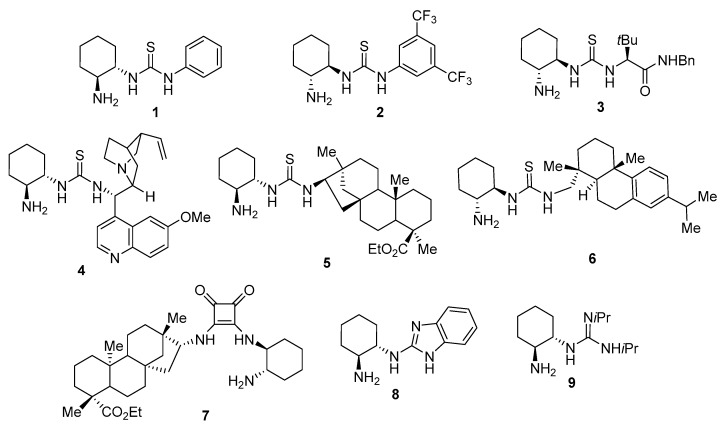
Chiral *trans*-cyclohexa-1,2-diamine-based organocatalysts employed in the enantioselective conjugate addition of aldehydes to nitroalkenes.

Nowadays, the enantioselective 1,4-addition reaction of enolizable aldehydes to nitroalkenes promoted by a chiral organocatalyst is one of the most common and convenient procedures for achieving the synthesis of γ-nitroaldehydes in an enantiomerically enriched form [7,8,9,10,11,12]. Particularly, bifunctional organocatalysts derived from enantiopure *trans*-cyclohexane-1,2-diamine, a commonly employed chiral auxiliary [13,14], bearing a primary amine and an additional H-bond-forming functionality, have been employed successfully in this transformation (Figure 1). Thus, the use of primary amine-thioureas has been frequent, as exemplified in organocatalyst **1** [15], **2** [16] and **3** [17], as well as the *Cinchona-*derived **4** [18], the isosteviol- and rosin-derived **5** [19] and **6** [20], respectively, and even calix[4]arene-derived compounds [21]. In addition, primary-amine squaramides have been used, as is the case of isosteviol-derived compound **7** [22], and the primary-amine-containing benzimidazole **8** [23] and guanidine **9** [24]. In all these primary-amine organocatalysts, the enantioselectivity is induced by addition of a transient enamine to the nitroolefin, which is H-bond-coordinated by the NO_2_ group to the NH’s of the catalyst functionality.

**Figure 2:**
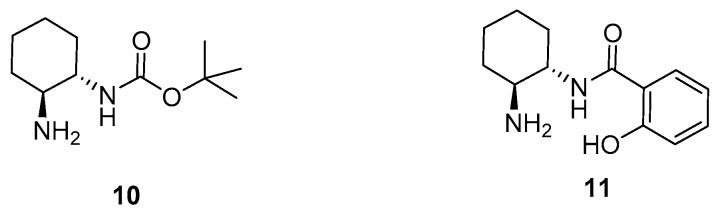
Organocatalysts employed in this study.

In the last years, our group has been involved in the use of small-size *trans*-cyclohexane-1,2-diamine-derived monocarbamates, such as the mono-Boc-protected diamine **10** as organocatalysts in the enantioselective conjugate addition reactions of carbonyl compounds to electron-deficient olefins (Figure 2) [25,26,27,28]. This paper shows now that a simple monoamidation of a chiral *trans*-cyclohexane-1,2-diamine with salicylic acid affords a primary amine-bearing salicylamide **11** (Figure 2), suitable to organocatalyze the asymmetric conjugate addition reaction of the ‘difficult’ α,α-disubstituted aldehydes to nitroalkenes, leading to enantioenriched γ-nitroaldehydes. Theoretical calculations can explain the observed enantioselectivity of the process.

## 2. Results and Discussion

The primary amine-salicylamide **11** employed as organocatalyst in this study was prepared by monoamidation of (1*S*,2*S*)-cyclohexane-1,2-diamine with phenyl salicylate in refluxing propan-2-ol [29]. The search for the most appropriate reaction conditions (Table 1) was carried out using the model conjugate addition reaction of isobutyraldehyde (**12a**) to *trans*-β-nitrostyrene (**13a**). Thus, this reaction organocatalyzed by **11** (20 mol %) in toluene as a solvent at room temperature afforded the corresponding γ-nitroaldehyde (*S*)-**14aa** in 79% *ee* but in a very low yield after 2 d reaction time (Table 1, entry 1). The (*S*) absolute configuration of the final adduct was determined by comparison of the elution order of the corresponding enantiomers in chiral HPLC with those in the literature [24]. The use of dimethylformamide (DMF) as solvent lowered down dramatically the stereoselectivity of the process (Table 1, entry 2), whereas the use of dichloromethane raised the enantioselectivity again up to 84%, but with almost negligible yield (Table 1, entry 3).

The addition of acid or basic additives frequently has proven beneficial in the organocatalyzed conjugate addition of carbonyl compounds to nitroalkenes, not only accelerating the formation of the transient enamine, but supposedly avoiding the formation of stable catalyst-derived byproducts [12]. Therefore, we assayed the model conjugate addition reaction in the presence of 4-dimethylaminopyridine (DMAP, 20 mol %) as a basic additive in dichloromethane as a solvent, now obtaining adduct (*S*)-**14aa** in an 81% isolated yield and with an enantioselectivity of 92% (Table 1, entry 4). However, the use of other basic additives resulted in being not so effective. Thus, the presence of imidazole or pyridine as additives gave lower enantioselections and very poor yields (Table 1, entries 5 and 6), whereas other basic species such as TMG, DBU or DABCO showed less efficiency than DMAP, considering the isolated yield of (*S*)-**14aa** (Table 1, entries 7–9). In addition, we also attempted the use of aromatic carboxylic acids as additives, but, in all cases, the achieved enantioselectivities were just moderate and yields were very low (Table 1, entries 10–12).

Considering the couple **11**/DMAP as the most efficient catalytic combination, we explore the influence of the ratio between both components. Thus, lowering the organocatalyst loading down to 10 mol % and keeping the additive loading to 20 mol % gave rise to (*S*)-**14aa** in only 17% yield but in 94% *ee* (Table 1, entry 13), whereas keeping the catalyst loading in 20 mol % and diminishing the amount of DMAP to 10 mol % maintained the *ee* unaltered, increasing the final yield (Table 1, entry 14). However, loadings of 20 mol % of **11** and 30 mol % of DMAP allowed to obtain (*S*)-**14aa** in 72% yield and 95% *ee* (Table 1, entry 15).

**Figure 3:**
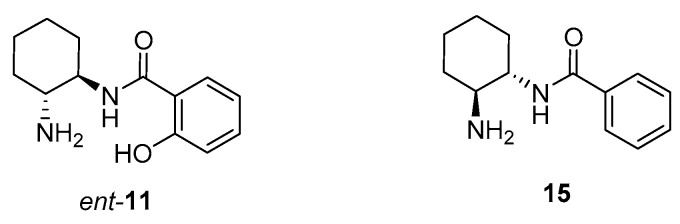
Organocatalysts employed in this study.

Expecting to achieve an opposite enantioselection, we also performed the reaction using as organocatalyst *ent-***11**, which was prepared similarly to its enantiomeric counterpart, but using (1*R*,2*R*)-cyclohexane-1,2-diamine as chirality source (Figure 3). Using this primary amine as organocatalyst (20 mol %) under the most effective reaction conditions (DMAP (30 mol %), CH_2_Cl_2_, rt), adduct (*R*)-**14aa** was isolated in the same 95% enantioselectivity than its opposite (*S*)-enantiomer (Table 1, entry 16).

We were intrigued to determine if the presence of the phenolic OH on the organocatalyst was a determinant for the high enantioselectivity obtained. Thus, we prepared the primary amine-containing benzamide **15** by reaction of (1*S*,2*S*)-cyclohexane-1,2-diamine with phenyl benzoate under similar conditions as **11** (Figure 3). However, under the above optimal reaction conditions, this organocatalyst **15** gave rise to adduct (*S*)-**14aa** in only a 79% *ee* (Table 1, entry 17). Therefore, the presence of the phenolic OH in organocatalyst **10** results as being important for achieving a good enantioinduction. It is interesting to note that the use of the monocarbamate **10** as an organocatalyst was not particularly successful, giving rise to the corresponding adduct in low yield and only 65% *ee* (Table 1, entry 18), whereas it has been able to reach up to 96% *ee* when employed in a related enantioselective Michael addition of aromatic ketones to nitroalkenes [27].

Next, we extended the addition reaction of isobutyraldehyde to other trans-β-nitroalkenes **13** under the most favorable reaction conditions (**11** (20 mol %), DMAP (30 mol %), CH_2_Cl_2_, rt), the results being summarized in Table 2. The absolute configuration of the known γ-nitroaldehydes **14** was assigned in accordance with the elution order of the enantiomers in chiral HPLC when compared to the literature (see Experimental Section).

Thus, when nitroalkenes **13b** and **13c**, bearing electron-releasing groups such as methyl or methoxy in the aromatic ring, were used, the corresponding Michael adducts (*S*)-**14ab** and (*S*)-**14ac** were both isolated in enantioselectivities of 92% (Table 2, entries 2 and 3). In addition, the presence of a dioxolane system on the aromatic ring, as in the nitroolefin **13d**, lowered the enantioselection down to 85% (Table 2, entry 4), whereas this was raised up to 94% when **13e** containing three methoxy groups was used as electrophile (Table 2, entry 5). When halogen groups were present onto the aromatic ring of the nitroalkene, the achieved enantioselectivities were rather uneven. Thus, a fluoro group (**13f**) gave rise to (*S*)-**14af** in 92% *ee*, whereas chloro groups at 2- (**13g**) and 4-positions (**13h**) afforded adducts (*R*)-**14ag** and (*S*)-**14ah** in similar 87 and 88% *ee*, respectively (Table 2, entries 6–8), and a bromo group (**13i**) gave (*S*)-**14ai** in 94% *ee* (Table 2, entry 9). Moreover, an electron-withdrawing group such as the trifluoromethyl (**13j**) gave also a good enantioselectivity for (*S*)-**14aj** (93%) (Table 2, entry 10).

When nitroalkene **13k** bearing a 2-naphthyl group was employed as Michael acceptor, the corresponding adduct (*S*)-**14ak** was obtained in 91% *ee* (Table 2, entry 11). In addition, the influence of the presence of heteroarylated rings in the nitroalkene was also explored with the use as Michael acceptors of the 3-pyridinyl- and 2-furanyl-containing nitroalkenes **13l** and **13m**, which gave rise to adducts (*S*)-**14al** and (*S*)-**14am** in 91 and 92% *ee*, respectively (Table 2, entries 12 and 13).

We also explored the conjugate addition reaction of other α,α-disubstituted aldehydes with nitroalkene **13a**. Thus, when cyclopentanecarbaldehyde (**12b**) was used, the corresponding Michael adduct (*S*)-**14ba** was isolated in an excellent 94% *ee* (Table 2, entry 14). However, when diphenylacetaldehyde or 2-phenylpropionaldehyde were used as pro-nucleophiles, almost negligible amounts (5%) of the corresponding adducts were detected as racemates.

**Figure 4:**
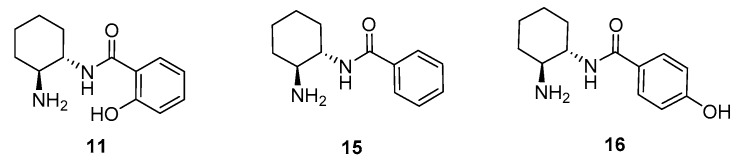
Model catalysts employed in the computational study.

To get further insight into the origin of the enantioselectivity, we carried out theoretical calculations on the reaction between isobutyraldehyde **12a** and nitroalkene **13a** in the presence of catalyst **11**. A salient feature of this catalyst is the presence of a phenolic OH in the *ortho* position, which seems to play a key role to enhance the activity and/or selectivity of the process. To understand this intriguing behavior, the results obtained with **11** were compared with those of the less active catalyst **15**, and also with the model system **16** (Figure 4), which bears the OH group *para* to the carbonyl substituent. This species was not checked experimentally but would give us a better understanding of the relevance of the *ortho* substitution for the reactivity.

**Figure 5:**
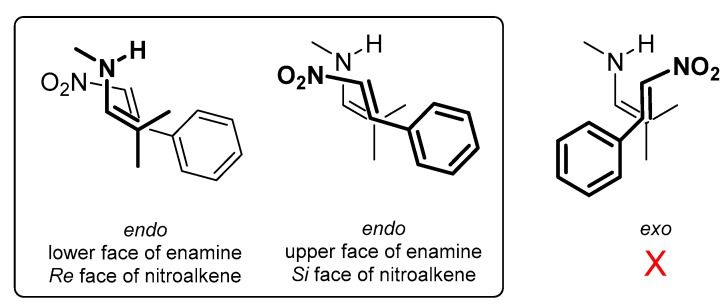
Seebach’s synclinal model for the approach of enamine and nitroalkene faces.

As expected, the initial formation of an enamine between the catalyst free amine and the aldehyde is followed by attack to the nitrostyrene according to Seebach’s synclinal model (*endo* attack) [30,31], which was confirmed by the much higher energy of other possible approaches, like *exo* depicted in Figure 5. The synclinal model secures a diastereoselective approach of the reacting faces of enamine and alkene, meaning that the lower face (in our view) of the enamine reacts with the *Re* face of alkene and the opposite, the upper face of enamine with the *Si* face of alkene. As a direct consequence, the approach of the alkene from each side of the enamine produces a single diastereoisomer.

**Figure 6:**
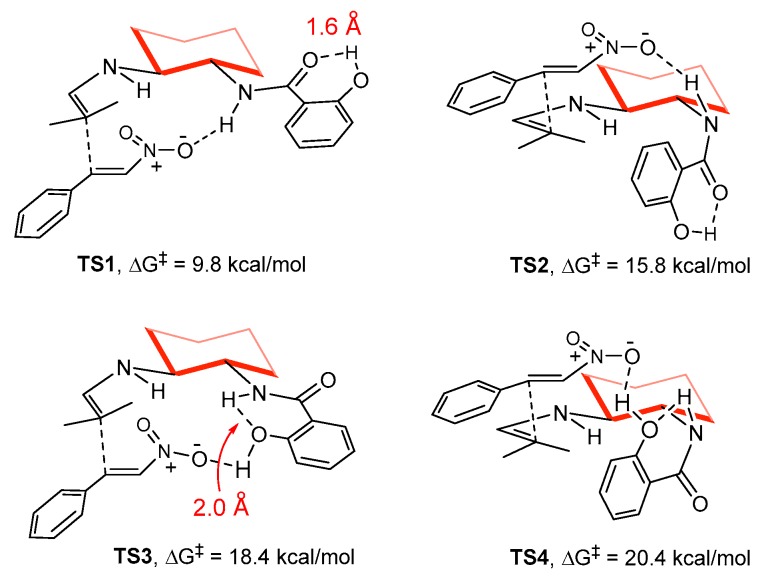
Computed Free energies and structures of the diastereoselective transition states for the C-C bond formation in the presence of catalyst **11**.

The different conformations of the both cyclohexyl-amine substituents in the catalysts were taken into consideration. The amine side arms of cyclohexane possess several free rotating bonds, and, among all the possibilities checked, we selected the most stable ones, outlined in Figure 6. In them, the enamine and amide groups occupy the equatorial positions of the cyclohexane, pointing up and down, respectively. The fragment NH-C-C-NH is in staggered conformation. We then located the transition states for **11**, corresponding to the different approaches, taking into account all possible H-bonding activation networks of the NH and OH groups (Figure 6). As expected, the amide NH group in **11** is H-binding the nitro group of the electrophile, activating it for the nucleophilic attack, and inducing a good differentiation of both faces of the enamine. The *lower face* approach in **TS1** is much lower in energy (9.8 kcal/mol) than **TS2** (15.8 kcal/mol), justifying the experimental selectivity. The origin of the selectivity is clearly linked to the high strain developing in **TS2** to accommodate the H-bond between the nitro and amide groups. We could demonstrate that OH is not actively participating in the activation of the nitro group, since the barrier in **TS3** is clearly higher (18.4 kcal/mol) than in **TS1**, even if the amide-NH in **TS3** is slightly contributing to increase the acidity of the OH through Brønsted assistance (δ_O–H_ = 1.98 Å). Interestingly, this H-bonding network alternative is more flexible, and the energy difference between **TS3** and **TS4** is fairly reduced to only 2.0 kcal/mol.

**Figure 7:**
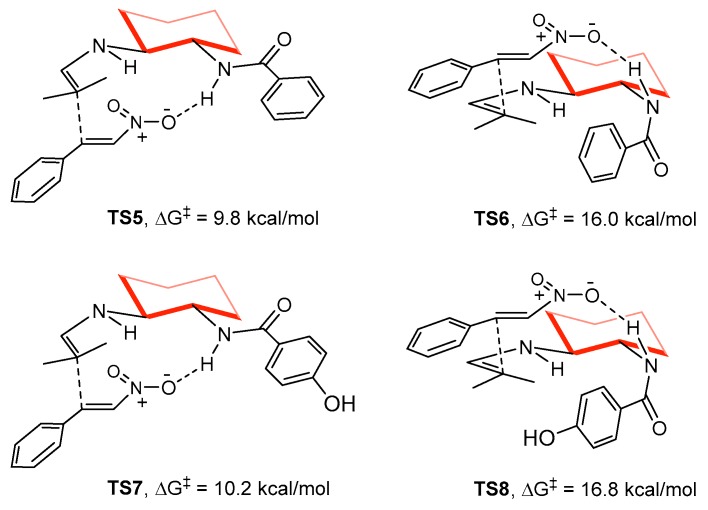
Computed Free energies and structures of the diastereoselective transition states for the C-C bond formation in the presence of the catalysts **15** and **16**.

Initially, the tentative role of OH in promoting the reaction was assigned to its suitability to enhance the H-bond donor ability of the amide group through an extra activation by Brønsted acid-assistance (δ_O–H_ = 1.62 Å in **TS1**). However, to our surprise, the calculations predicted a similar reactivity and selectivity for species **15** and **16** (Figure 4), where the OH group is lacking or cannot interact with the amide because it is positioned at the *para* position. In fact, the activation energies of transition states **TS5** and **TS7** are undistinguishable from **TS1** (Figure 7), and a similar situation is found comparing **TS2**, **TS6** and **TS8**. Thus, the presence or absence of the *ortho* OH group in **11** is not predicted computationally to have a significant effect on both reactivity and selectivity.

**Figure 8:**
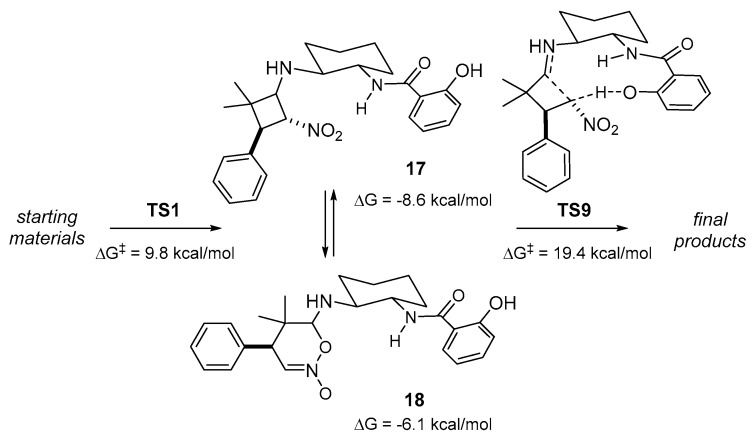
Computed Free energies and structures for the formation of the cyclic intermediates and their protonation to the final products.

In this regard, there is an increasing number of studies indicating that C-C bond formation between enamine and nitroalkene is facile and might not be the rate-determining step in catalyzed processes [32,33,34]. Instead, the highest energy along the reaction coordinate would correspond to the protonation of the highly stable cyclic intermediates that arise after the C-C bond formation. Thus, the rate- and stereodetermining steps might not be identical. We checked this alternative in our case, and could locate two cyclic intermediates, **17** and **18** (Figure 8), showing energies of −8.6 and −6.1 kcal/mol with respect to the starting materials. Furthermore, the lowest transition state for their protonation (**TS9**) would be the rate limiting step of our cycle, presenting a Free energy barrier of 28.0 kcal/mol (from **17**).

A similar protonation cannot obviously be envisioned for catalytic species lacking *ortho*-phenol, like **15** and **16**, and, in those cases, adventitious water must be responsible for the cleavage and protonation of the proposed intermediates, although at a higher energetic cost. As a confirmation, we were able to locate some transition states involving a molecule of water, which are in every case at least 1.5–2.0 kcal/mol higher in energy than **TS9**. Thus, we can confirm that also in our case the stereoselectivity is controlled during the C-C bond formation event, but the reaction rate is governed by the protonation and cleavage of the stable cyclic intermediates. It is at this point where the phenol moiety plays a crucial role, nicely explaining the experimental results.

## 3. Experimental Section

### 3.1. General Information

All the reagents and solvents employed were of the best grade available and were used without further purification. The ^1^H- and ^13^C-NMR spectra were recorded at room temperature on a Bruker (Bruker, Billerica, MA, USA) AC-400 at 400 MHz and 101 MHz, respectively, using TMS as internal standard. IR spectra were measured on a Nicolet Impact 400D-FT instrument (Thermo Fisher Scientific, Waltham, MA, USA). Electron Ionized Mass Spectrometry (EIMS) spectra were obtained on an Agilent Technologies GC/MS-5973N equipment (Agilent Technologies, Santa Clara, CA, USA) at 70 eV. HR-MS spectra were obtained on an Agilent Technologies 7200 Accurate-Mass Q-TOF GC/MS equipment at using EI at 70 eV. Compounds **11** and *ent*-**11** were obtained as described [29]. Nitroalkenes **13** were purchased or prepared following a reported procedure [35], except **13l,** which was obtained differently [36]. Absolute configuration for adducts **14** was determined according to the order of elution of their enantiomers in chiral HPLC. The absolute configuration of the not described compounds **14ad** and **14ae** was assigned by analogy. Reference racemic samples of adducts **14** were obtained by performing the conjugate addition reaction using 4-methylbenzylamine (20 mol %) as organocatalyst in toluene as a solvent at room temperature.

### 3.2. General Procedure for the Asymmetric Conjugate Addition Reaction

To a solution of **10**, **11**, *ent*-**11** or **15** (0.04 mmol), the nitroalkene **13** (0.2 mmol) and DMAP (7.3 mg, 0.06 mmol) in CH_2_Cl_2_ (0.3 mL) was added the aldehyde **12** (0.4 mmol) and the mixture was stirred at rt until completion (TLC). The reaction was quenched with HCl 2N (10 mL) and the mixture was extracted with AcOEt (3 × 10 mL). The organic phase was washed with sat. NaHCO_3_ (2 × 10 mL), dried over MgSO_4_, and the solvent was evaporated (15 Torr) to get the crude product, which was purified by silica gel chromatography (*n*-hexane/AcOEt gradients). Known adducts **14** were identified by comparison of their NMR data with those of the literature (Supplementary Materials NMR spectra). Their enantiomeric excesses were determined by chiral HPLC using the conditions described in each case (Supplementary Materials HPLC chromatograms). Not described compounds **14ad** and **14ae** have been fully characterized.

*2,2-Dimethyl-4-nitro-3-phenylbutanal* (**14aa**) [24]. Colorless oil; ^1^H-NMR (CDCl_3_): δ*_H_* = 9.52 (s, 1H), 7.35–7.28 (m, 3H), 7.22–7.17 (m, 2H), 4.85 (dd, *J* = 13.1, 11.3 Hz, 1H), 4.69 (dd, *J* = 13.1, 4.2 Hz, 1H), 3.79 (dd, *J* = 11.3, 4.2 Hz, 1H), 1.12 (s, 3H), 0.99 (s, 3H) ppm; ^13^C-NMR (CDCl_3_): δ*_C_* = 204.2, 135.3, 129.0, 128.6, 128.0, 76.2, 48.3, 48.1, 21.5, 18.7 ppm; HPLC: Chiralpak OD-H, λ = 210 nm, *n*-hexane/2-propanol, 80:20, 1.0 mL/min, t_r_ (*R*) = 13.4 min, t_r_ (*S*) = 19.2 min.

*2,2-Dimethyl-4-nitro-3-(*p*-tolyl)butanal* (**14ab**) [24]. Colorless oil; ^1^H-NMR (CDCl_3_): δ*_H_* = 9.52 (s, 1H), 7.13 (d, *J* = 8.0 Hz, 2H), 7.08 (d, *J* = 8.2 Hz, 2H), 4.83 (dd, *J* = 12.9, 11.4 Hz, 1H), 4.67 (dd, *J* = 12.9, 4.2 Hz, 1H), 3.74 (dd, *J* = 11.4, 4.2 Hz, 1H), 2.32 (s, 3H), 1.12 (s, 3H), 1.00 (s, 3H) ppm; ^13^C-NMR (CDCl_3_): δ*_C_* = 204.4, 137.8, 132.1, 129.3, 128.9, 76.3, 48.2, 48.1, 21.5, 21.0, 18.8 ppm; HPLC: Chiralpak OD-H, λ = 210 nm, *n*-hexane/2-propanol, 80:20, 1.0 mL/min, t_r_ (*R*) = 9.7 min, t_r_ (*S*) = 13.2 min.

*3-(4-Methoxyphenyl)-2,2-dimethyl-4-nitrobutanal* (**14ac**) [24]. Colorless oil; ^1^H-NMR (CDCl_3_): δ*_H_* = 9.51 (s, 1H), 7.12 (d, *J* = 8.7 Hz, 2H), 6.85 (d, *J* = 8.8 Hz, 2H), 4.81 (dd, *J* = 12.8, 11.4 Hz, 1H), 4.66 (dd, *J* = 12.8, 4.2 Hz, 1H), 3.78 (s, 3H), 3.73 (dd, *J* = 11.4, 4.2 Hz, 1H), 1.11 (s, 3H), 0.99 (s, 3H) ppm; ^13^C-NMR (CDCl_3_): δ*_C_* = 204.4, 159.2, 130.0, 127.0, 114.0, 76.4, 55.1, 48.3, 47.7, 21.4, 18.7 ppm; HPLC: Chiralpak OD-H, λ = 210 nm, *n*-hexane/2-propanol, 80:20, 1.0 mL/min, t_r_ (*R*) = 12.0 min, t_r_ (*S*) = 16.1 min.

*3-(Benzo[*d*]*[1,3]*dioxol-5-yl)-2,2-dimethyl-4-nitrobutanal* (**14ad**). Colorless oil; IR (ATR): *ν* = 2972, 2904, 2817, 1722, 1552, 1491, 1444, 1376, 1241, 1038, 931, 816 cm^−1^; ^1^H-NMR (CDCl_3_): δ*_H_* = 9.51 (s, 1H), 6.75 (d, *J* = 8.0 Hz, 1H), 6.69 (d, *J* = 1.8 Hz, 1H), 6.65 (dd, *J* = 8.0, 1.8 Hz, 1H), 5.96 (s, 2H), 4.78 (dd, *J* = 13.0, 11.4 Hz, 1H), 4.65 (dd, *J* = 13.0, 4.2 Hz, 1H), 3.70 (dd, *J* = 11.4, 4.2 Hz, 1H), 1.13 (s, 3H), 1.02 (s, 3H) ppm; ^13^C-NMR (CDCl_3_): δ*_C_* = 204.2, 147.9, 147.4, 128.8, 122.6, 109.1, 108.3, 101.2, 76.5, 48.3, 21.6, 19.0 ppm; MS (EI, 70 eV): *m*/*z* (%) = 265 (M^+^, 10), 148 (100); HR-MS (EI): *m*/*z* calcd. for C_13_H_15_NO_5_ [M]^+^: 265.0952, found: 265.0947; HPLC: Chiralpak OD-H, λ = 210 nm, *n*-hexane/2-propanol, 80:20, 1.0 mL/min, t_r_ (*R*) = 17.2 min, t_r_ (*S*) = 23.0 min.

*2,2-Dimethyl-4-nitro-3-(3,4,5-trimethoxyphenyl)butanal* (**14ae**). Colorless oil; IR (ATR): ν = 2972, 2939, 2835, 1722, 1589, 1552, 1460, 1242, 1124, 1005, 729 cm^−1^; ^1^H-NMR (CDCl_3_): δ*_H_* = 9.52 (s, 1H), 6.38 (s, 2H), 4.85 (dd, *J* = 13.1, 11.3 Hz, 1H), 4.69 (dd, *J* = 13.1, 4.2 Hz, 1H), 3.85 (s, 6H), 3.83 (s, 3H), 3.70 (dd, *J* = 11.3, 4.2 Hz, 1H), 1.16 (s, 3H), 1.06 (s, 3H) ppm; ^13^C-NMR (CDCl_3_): δ*_C_* = 204.3, 153.1, 137.7, 131.0, 106.2, 76.3, 60.7, 56.1, 48.9, 48.2, 21.7, 19.3 ppm; MS (EI, 70 eV): *m*/*z* (%) = 311 (M^+^, 16), 194 (100), 179 (35); HR-MS (EI): *m*/*z* calcd. for C_15_H_21_NO_6_ [M]^+^: 311.1369, found: 311.1367; HPLC: Chiralpak OD-H, λ = 210 nm, *n*-hexane/2-propanol, 80:20, 1.0 mL/min, t_r_ (*R*) = 18.2 min, t_r_ (*S*) = 20.7 min.

*3-(4-Fluorophenyl)-2,2-dimethyl-4-nitrobutanal* (**14af**) [24]. Colorless oil; ^1^H-NMR (CDCl_3_): δ*_H_* = 9.51 (s, 1H), 7.21–7.17 (m, 2H), 7.05–7.01 (m, 2H), 4.82 (dd, *J* = 13.1, 11.4 Hz, 1H), 4.69 (dd, *J* = 13.1, 4.2 Hz, 1H), 3.78 (dd, *J* = 11.4, 4.2 Hz, 1H), 1.12 (s, 3H), 1.01 (s, 3H) ppm; ^13^C-NMR (CDCl_3_): δ*_C_* = 204.0, 162.4 (d, *J* = 247.4 Hz), 131.2 (d, *J* = 3.1 Hz), 130.6 (d, *J* = 8.2 Hz), 115.7 (d, *J* = 21.5 Hz), 76.3, 48.2, 47.7, 21.6, 18.8 ppm; HPLC: Chiralpak OD-H, λ = 210 nm, *n*-hexane/2-propanol, 80:20, 1.0 mL/min, t_r_ (*R*) = 11.1 min, t_r_ (*S*) = 17.8 min.

*3-(2-Chlorophenyl)-2,2-dimethyl-4-nitrobutanal* (**14ag**) [37]. Colorless oil; ^1^H-NMR (CDCl_3_): δ*_H_* = 9.55 (s, 1H), 7.44–7.40 (m, 1H), 7.31–7.21 (m, 3H), 4.89–4.80 (m, 1H), 4.73 (dd, *J* = 13.3, 4.1 Hz, 1H), 4.63 (dd, *J* = 11.3, 3.5 Hz, 1H), 1.17 (s, 3H), 1.08 (s, 3H) ppm; ^13^C-NMR (CDCl_3_): δ*_C_* = 203.8, 135.8, 133.7, 130.4, 129.1, 128.2, 127.1, 76.2, 49.0, 42.4, 20.9, 18.6 ppm; HPLC: Chiralpak OD-H, λ = 210 nm, *n*-hexane/2-propanol, 80:20, 1.0 mL/min, t_r_ (*S*) = 11.1 min, t_r_ (*R*) = 27.9 min.

*3-(4-Chlorophenyl)-2,2-dimethyl-4-nitrobutanal* (**14ah**) [24]. Colorless oil; ^1^H-NMR (CDCl_3_): δ*_H_* = 9.50 (s, 1H), 7.32 (d, *J* = 8.5 Hz, 2H), 7.15 (d, *J* = 8.5 Hz, 2H), 4.83 (dd, *J* = 13.1, 11.4 Hz, 1H), 4.69 (dd, *J* = 13.1, 4.2 Hz, 1H), 3.77 (dd, *J* = 11.4, 4.2 Hz, 1H), 1.12 (s, 3H), 1.01 (s, 3H) ppm; ^13^C-NMR (CDCl_3_): δ*_C_* = 203.8, 134.1, 133.9, 130.4, 128.9, 76.1, 48.1, 47.8, 21.7, 18.8 ppm; HPLC: Chiralpak OD-H, λ = 210 nm, *n*-hexane/2-propanol, 80:20, 1.0 mL/min, t_r_ (*R*) = 11.9 min, t_r_ (*S*) = 17.6 min.

*3-(4-Bromophenyl)-2,2-dimethyl-4-nitrobutanal* (**14ai**) [24]. Colorless oil; ^1^H-NMR (CDCl_3_): δ*_H_* = 9.50 (s, 1H), 7.47 (d, *J* = 8.5 Hz, 2H), 7.09 (d, *J* = 8.5 Hz, 2H), 4.82 (dd, *J* = 13.2, 11.4 Hz, 1H), 4.69 (dd, *J* = 13.2, 4.1 Hz, 1H), 3.76 (dd, *J* = 11.4, 4.1 Hz, 1H), 1.12 (s, 3H), 1.01 (s, 3H) ppm; ^13^C-NMR (CDCl_3_): δ*_C_* = 203.8, 134.5, 131.9, 130.7, 122.2, 76.0, 48.1, 47.9, 21.7, 18.9 ppm; HPLC: Chiralpak OD-H, λ = 210 nm, *n*-hexane/2-propanol, 80:20, 1.0 mL/min, t_r_ (*R*) = 14.0 min, t_r_ (*S*) = 19.6 min.

*2,2-Dimethyl-4-nitro-3-(4-(trifluoromethyl)phenyl)butanal* (**14aj**) [38]. Colorless oil; ^1^H-NMR (CDCl_3_): δ*_H_* = 9.50 (s, 1H), 7.61 (d, *J* = 8.2 Hz, 3H), 7.36 (d, *J* = 8.2 Hz, 2H), 4.89 (dd, *J* = 13.3, 11.4 Hz, 1H), 4.74 (dd, *J* = 13.3, 4.1 Hz, 1H), 3.88 (dd, *J* = 11.4, 4.1 Hz, 1H), 1.14 (s, 3H), 1.02 (s, 3H) ppm; ^13^C-NMR (CDCl_3_): δ*_C_* = 203.5, 139.8, 130.4 (q, *J* = 32.7 Hz), 125.7 (q, *J* = 3.6 Hz), 123.8 (q, *J* = 272.2 Hz), 75.9, 48.1, 21.8, 18.9 ppm; HPLC: Chiralpak OD-H, λ = 210 nm, *n*-hexane/2-propanol, 80:20, 1.0 mL/min, t_r_ (*R*) = 11.7 min, t_r_ (*S*) = 18.5 min.

*2,2-Dimethyl-3-(naphthalen-2-yl)-4-nitrobutanal* (**14ak**) [24]. Colorless oil; ^1^H-NMR (CDCl_3_): δ*_H_* = 9.57 (s, 1H), 7.84–7.82 (m, 3H), 7.68 (d, *J* = 1.3 Hz, 1H), 7.54–7.46 (m, 2H), 7.34 (dd, *J* = 8.5, 1.9 Hz, 1H), 5.00 (dd, *J* = 13.1, 11.3 Hz, 1H), 4.79 (dd, *J* = 13.1, 4.1 Hz, 1H), 3.97 (dd, *J* = 11.3, 4.1 Hz, 1H), 1.19 (s, 3H), 1.05 (s, 3H) ppm; ^13^C-NMR (CDCl_3_): δ*_C_* = 204.2, 133.0, 132.9, 132.8, 128.4, 128.3, 127.8, 127.6, 126.5, 126.5, 126.3, 76.3, 48.6, 48.4, 21.7, 19.0 ppm; HPLC: Chiralpak OD-H, λ = 210 nm, *n*-hexane/2-propanol, 70:30, 1.0 mL/min, t_r_ (*R*) = 16.4 min, t_r_ (*S*) = 29.6 min.

*2,2-Dimethyl-4-nitro-3-(pyridin-3-yl)butanal* (**14al**) [24]. Colorless oil; ^1^H-NMR (CDCl_3_): δ*_H_* = 9.50 (s, 1H), 8.56 (d, *J* = 3.9 Hz, 1H), 8.51 (s, 1H), 7.63–7.57 (m, 1H), 7.30 (dd, *J* = 8.3, 5.2 Hz, 1H), 4.89 (dd, *J* = 13.3, 11.4 Hz, 1H), 4.75 (dd, *J* = 13.3, 4.1 Hz, 1H), 3.83 (dd, *J* = 11.4, 4.1 Hz, 1H), 1.15 (s, 3H), 1.04 (s, 3H) ppm; ^13^C-NMR (CDCl_3_): δ*_C_* = 203.3, 150.4, 149.4, 136.2, 131.5, 123.5, 75.7, 48.2, 46.0, 21.8, 18.8 ppm; HPLC: Chiralpak AD-H, λ = 210 nm, *n*-hexane/2-propanol, 80:20, 1.0 mL/min, t_r_ (*S*) = 12.6 min, t_r_ (*R*) = 14.6 min.

*3-(Furan-2-yl)-2,2-dimethyl-4-nitrobutanal* (**14am**) [39]. Colorless oil; ^1^H-NMR (CDCl_3_): δ*_H_* = 9.52 (s, 1H), 7.37 (dd, *J* = 1.8, 0.6 Hz, 1H), 6.32 (dd, *J* = 3.3, 1.8 Hz, 1H), 6.22 (dd, *J* = 3.3, 0.6 Hz, 1H), 4.76 (dd, *J* = 12.9, 11.1 Hz, 1H), 4.59 (dd, *J* = 12.9, 3.9 Hz, 1H), 3.93 (dd, *J* = 11.1, 3.9 Hz, 1H), 1.18 (s, 3H), 1.05 (s, 3H) ppm; ^13^C-NMR (CDCl_3_): δ*_C_* = 203.4, 149.7, 142.7, 110.4, 109.6, 74.8, 48.1, 42.2, 21.1, 19.0 ppm; HPLC: Chiralpak OD-H, λ = 210 nm, *n*-hexane/2-propanol, 80:20, 1.0 mL/min, t_r_ (*R*) = 9.0 min, t_r_ (*S*) = 13.2 min.

*1-(2-Nitro-1-phenylethyl)cyclopentanecarbaldehyde* (**14ba**) [40]. Colorless oil; ^1^H-NMR (CDCl_3_): δ*_H_* = 9.49 (s, 1H), 7.34–7.19 (m, 5H), 4.96 (dd, *J* = 13.1, 11.4 Hz, 1H), 4.68 (dd, *J* = 13.1, 3.8 Hz, 1H), 3.70 (dd, *J* = 11.4, 3.8 Hz, 1H), 2.07–2.03 (m, 1H), 1.90–1.86 (m, 1H), 1.68–1.50 (m, 6H) ppm; ^13^C-NMR (CDCl_3_): δ*_C_* = 204.4, 136.4, 128.80, 128.78, 128.07, 77.3, 60.2, 49.3, 32.6, 31.5, 24.8, 24.6 ppm; HPLC: Chiralpak OD-H, λ = 210 nm, *n*-hexane/2-propanol, 80:20, 1.0 mL/min, t_r_ (*S*): 11.0 min, t_r_ (*R*): 14.4 min.

### 3.3. Computational Methods

All reported structures were optimized at Density Functional Theory level by using the B3LYP [41,42,43] functional as implemented in Gaussian 09 [44]. Optimizations were carried out with the 6-31G (d,p) basis set. The stationary points were characterized by frequency calculations in order to verify that they have the right number of imaginary frequencies. The reported energy values correspond to Gibbs Free energies, including single point refinements at M06-2X/6-311 + G (d,p) [45] level of theory in a solvent model (IEFPCM, dichloromethane) [46,47,48] on the previously optimized structures (Supplementary Materials computed structures).

## 4. Conclusions

We conclude that readily available primary amine-salicylamides, prepared by a simple monoamidation of enantiomerically pure *trans*-cyclohexane-1,2-diamines, act as efficient organocatalysts in the enantioselective conjugate addition of aldehydes to nitroalkenes, leading to enantiomerically enriched γ-nitroaldehydes. Good yields and high enantioselectivities can be achieved working in the presence of DMAP as a rate-accelerating additive. Theoretical calculations suggest that the stereoselectivity is defined during the C-C bond forming event, through a H-bond activation of the nitroalkene with the NH of the amide. The approach of the alkene happens preferentially through the lower face of the enamine, where the amide group is located. Meanwhile, the rate-determining step occurs at a later stage, corresponding to the protonation of downstream stable cyclic intermediates. The *ortho* phenolic moiety of the catalyst is able to act as an internal proton source, and its presence increases the protonation rate, accelerating the reaction.

## Figures and Tables

**Table 1:**
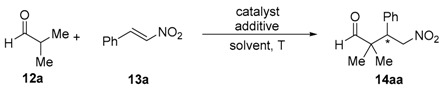
Screening and optimization of the reaction conditions for the model enantioselective conjugate addition.

Entry	Catalyst (mol %)	Additive (mol %) ^a^	Solvent	*t* (d)	Yield (%) ^b^	*ee* (%) ^c^
1	**11** (20)	-	PhMe	2	10 ^d^	79 (*S*)
2	**11** (20)	-	DMF	2	26	9 (*S*)
3	**11** (20)	-	CH_2_Cl_2_	2	10 ^d^	84 (*S*)
4	**11** (20)	DMAP (20)	CH_2_Cl_2_	2	81	92 (*S*)
5	**11** (20)	Imidazole (20)	CH_2_Cl_2_	2	10 ^d^	76 (*S*)
6	**11** (20)	Pyridine (20)	CH_2_Cl_2_	2	10 ^d^	81 (*S*)
7	**11** (20)	TMG (20)	CH_2_Cl_2_	2	43	91 (*S*)
8	**11** (20)	DBU (20)	CH_2_Cl_2_	2	31	38 (*S*)
9	**11** (20)	DABCO (20)	CH_2_Cl_2_	2	47	93 (*S*)
10	**11** (20)	PhCO_2_H (20)	CH_2_Cl_2_	2	10 ^d^	71 (*S*)
11	**11** (20)	4-O_2_NC_6_H_4_CO_2_H (20)	CH_2_Cl_2_	2	10 ^d^	78 (*S*)
12	**11** (20)	3,4-(MeO)_2_C_6_H_3_CO_2_H (20)	CH_2_Cl_2_	2	10 ^d^	75 (*S*)
13	11 (10)	DMAP (20)	CH_2_Cl_2_	2	17	94 (*S*)
14	**11** (20)	DMAP (10)	CH_2_Cl_2_	2	41	94 (*S*)
15	**11** (20)	DMAP (30)	CH_2_Cl_2_	2	72	95 (*S*)
16	*ent*-**11** (20)	DMAP (30)	CH_2_Cl_2_	2	74	95 (*R*)
17	**15** (20)	DMAP (30)	CH_2_Cl_2_	3	40	79 (*S*)
18	**10** (20)	DMAP (30)	CH_2_Cl_2_	3	30	65 (*S*)

^a^ DMAP: 4-Dimethylaminopyridine; TMG: 1,1,3,3-Tetramethylguanidine; DBU: 1,8-Diazabicyclo[5.4.0]undec-7-ene; DABCO: 1,4-Diazabicyclo[2.2.2]octane. ^b^ Isolated yield after flash chromatography. ^c^ Enantioselectivities and absolute stereochemistry determined by chiral HPLC (see Experimental Section). ^d^ Estimated by ^1^H-NMR (400 MHz).

**Table 2:**
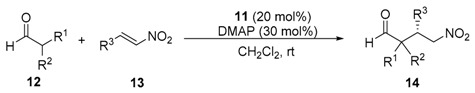
Enantioselective conjugate addition of aldehydes to nitroalkenes organocatalyzed by **11**.

Entry	Aldehyde		β-Nitroalkene		*t* (d)	γ-Nitroaldehyde		
	R^1^,R^2^	No.	R^3^	No.		No.	Yield (%) ^a^	*ee*^b^ (%) ^b^
1	Me,Me	**12a**	Ph	**13a**	2	(*S*)-**14aa**	72	95
2	Me,Me	**12a**	4-MeC_6_H_4_	**13b**	2	(*S*)-**14ab**	67	92
3	Me,Me	**12a**	4-MeOC_6_H_4_	**13c**	2	(*S*)-**14ac**	91	92
4	Me,Me	**12a**	3,4-(OCH_2_O)C_6_H_3_	**13d**	2	(*S*)-**14ad**	64	85
5	Me,Me	**12a**	3,4,5-(MeO)_3_C_6_H_2_	**13e**	2	(*S*)-**14ae**	85	94
6	Me,Me	**12a**	4-FC_6_H_4_	**13f**	2	(*S*)-**14af**	62	92
7	Me,Me	**12a**	2-ClC_6_H_4_	**13g**	2	(*R*)-**14ag** ^c^	50	87
8	Me,Me	**12a**	4-ClC_6_H_4_	**13h**	2	(*S*)-**14ah**	70	88
9	Me,Me	**12a**	4-BrC_6_H_4_	**13i**	2	(*S*)-**14ai**	50	94
10	Me,Me	**12a**	4-F_3_CC_6_H_4_	**13j**	2	(*S*)-**14aj**	51	93
11	Me,Me	**12a**	2-Naphthyl	**13k**	2	(*S*)-**14ak**	68	91
12	Me,Me	**12a**	3-Pyridinyl	**13l**	2	(*S*)-**14al**	43	91
13	Me,Me	**12a**	2-Furanyl	**13m**	2	(*S*)-**14am**	82	92
14	-(CH_2_)_4_-	**12b**	Ph	**13a**	3	(*S*)-**14ba**	60	94

^a^ Isolated yield after flash chromatography. ^b^ Enantioselectivities determined by chiral HPLC. Absolute configuration assigned by the order of elution of the enantiomers in chiral HPLC (See Experimental Section). ^c^ Apparent change in the sense of the enantioselectivity because of the application of the Cahn-Ingold-Prelog rules.

## Supplementary Materials

The following are available online, NMR spectra, HPLC chromatograms and cartesian coordinates of the computed structures.
