# Supplementary material for: Asymmetric Conjugate Addition of α,α-Disubstituted Aldehydes to Nitroalkenes Organocatalyzed by Chiral Monosalicylamides from trans-Cyclohexane-1,2-Diamines
Source: Molecules. 2018 Jan 11;23(1):141. doi: 10.3390/molecules23010141 (PMC6017890; doi:10.3390/molecules23010141)
Supplement: Supplementary file 1 [file molecules-23-00141-s001.pdf]

Suplementary Materials:

Asymmetric conjugate addition of α,α-disubstituted aldehydes to nitroalkenes organocatalyzed by chiral monosalicylamides from *trans*-cyclohexane-1,2-diamines

José R. Martínez-Guillén, Jesús Flores-Ferrándiz, Cecilia Gómez, Enrique Gómez-Bengoa *, Rafael Chinchilla *

NMR Spectra

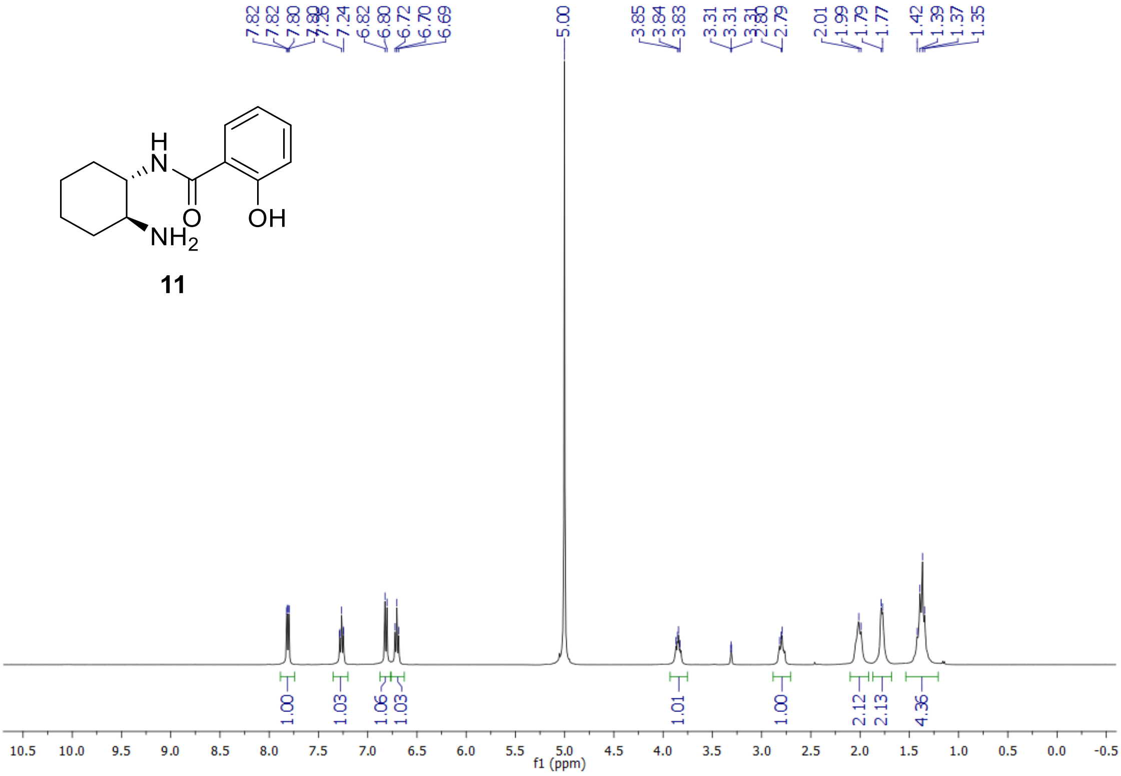

7.82
7.82
7.80
7.80
7.26
7.24
6.82
6.80
6.72
6.70
6.69
5.00
3.85
3.84
3.83
3.31
3.31
3.31
2.80
2.79
2.01
1.99
1.79
1.77
1.42
1.39
1.37
1.35
H
N
O
OH
NH_2
11
1.00
1.03
1.06
1.03
1.01
1.00
2.12
2.13
4.36
10.5 10.0 9.5 9.0 8.5 8.0 7.5 7.0 6.5 6.0 5.5 5.0 4.5 4.0 3.5 3.0 2.5 2.0 1.5 1.0 0.5 0.0 -0.5
f1 (ppm)

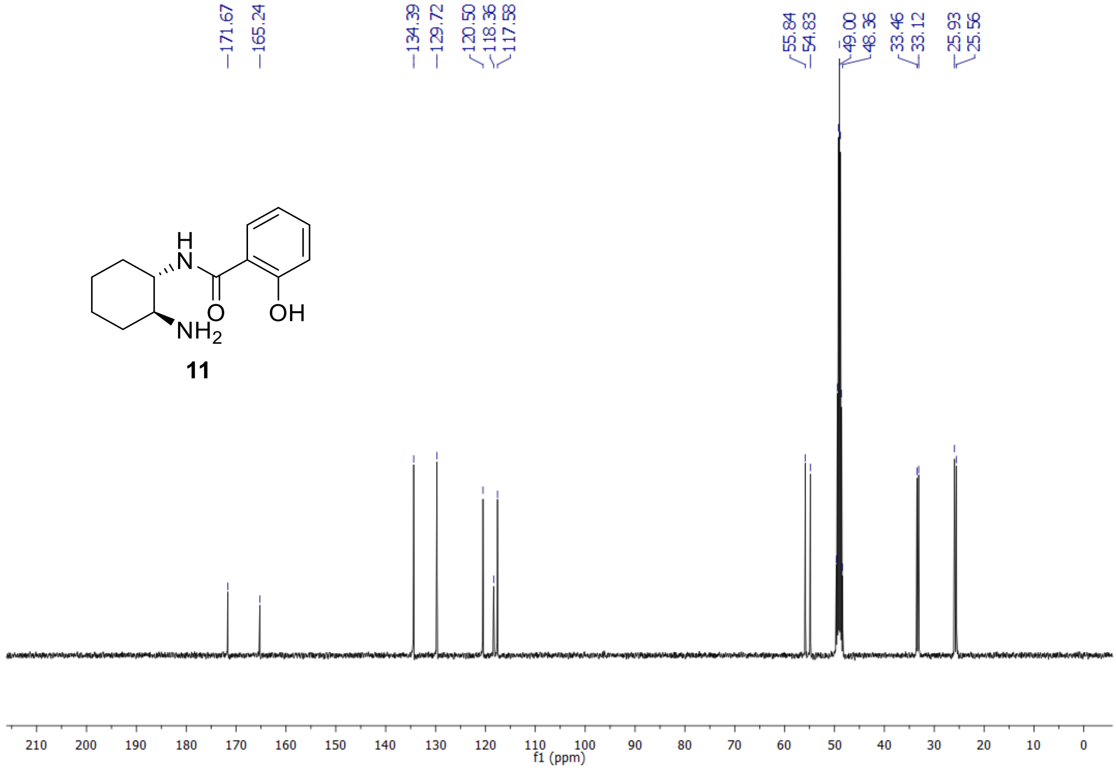

171.67
165.24
134.39
129.72
120.50
118.36
117.58
55.84
54.83
49.00
48.36
33.46
33.12
25.93
25.56
H
N
O
OH
NH_2
11
210 200 190 180 170 160 150 140 130 120 110 100 90 80 70 60 50 40 30 20 10 0
f1 (ppm)

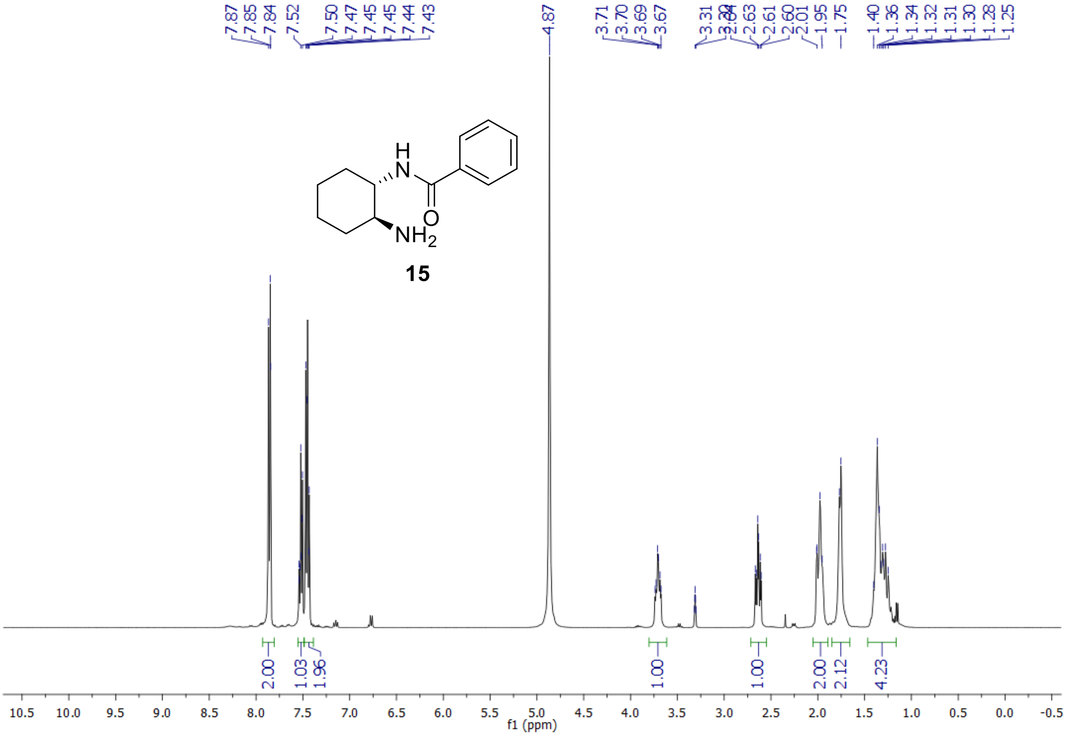
7.87
7.85
7.84
7.52
7.50
7.47
7.45
7.45
7.44
7.43
4.87
3.71
3.70
3.69
3.67
3.31
2.63
2.61
2.60
2.01
1.95
1.75
1.40
1.36
1.34
1.32
1.31
1.30
1.28
1.25
H
N
O
NH2
15
2.00
1.03
1.96
1.00
1.00
2.00
2.12
4.23
f1 (ppm)

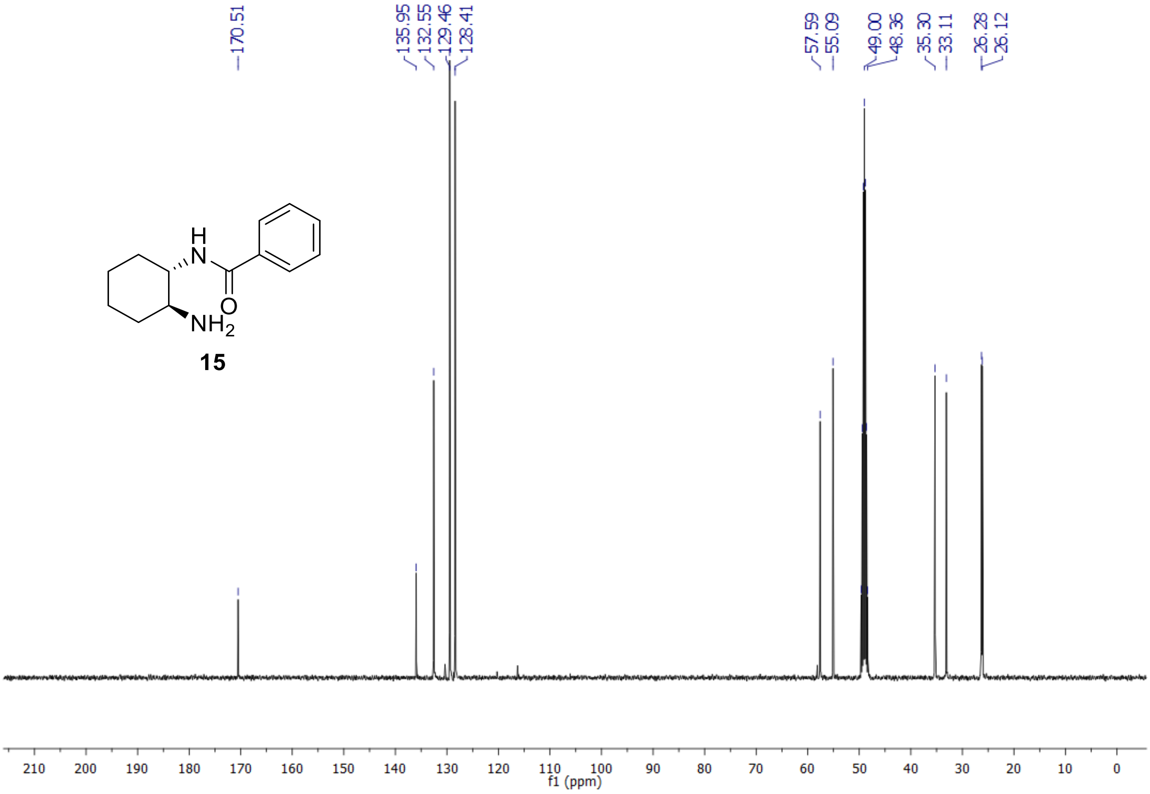
170.51
135.95
132.55
129.46
128.41
57.59
55.09
49.00
48.36
35.30
33.11
26.28
26.12
H
N
O
NH2
15
f1 (ppm)

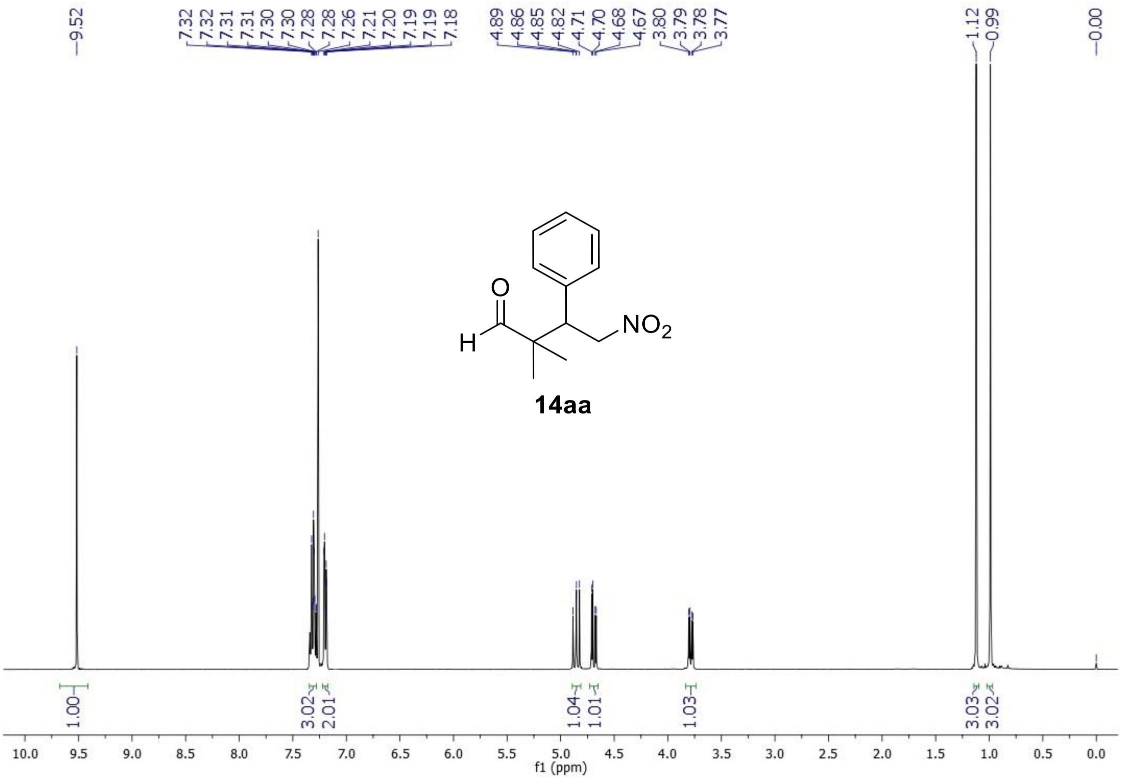
9.52
7.32 7.32 7.31 7.31 7.30 7.30 7.28 7.28 7.26 7.21 7.20 7.19 7.19 7.18
4.89 4.86 4.85 4.82 4.71 4.70 4.68 4.67
3.80 3.79 3.78 3.77
1.12 0.99
0.00
O
H
NO2
14aa
1.00
3.02 2.01
1.04 1.01
1.03
3.03 3.02
10.0 9.5 9.0 8.5 8.0 7.5 7.0 6.5 6.0 5.5 5.0 4.5 4.0 3.5 3.0 2.5 2.0 1.5 1.0 0.5 0.0
f1 (ppm)

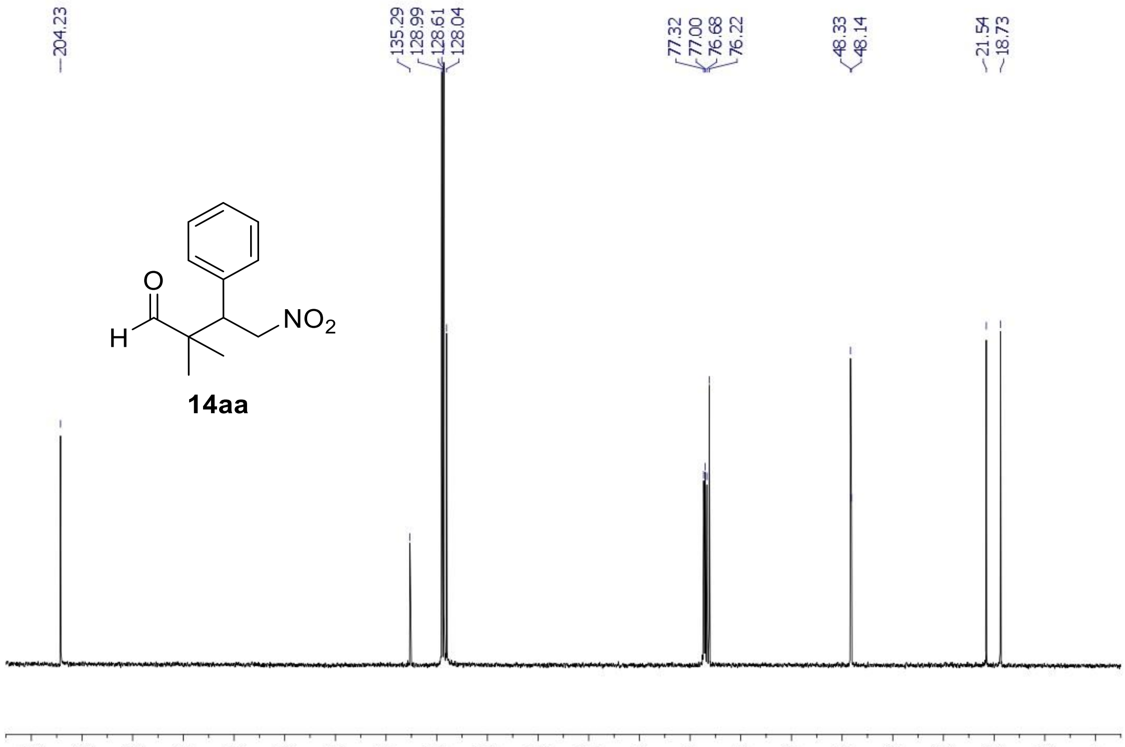
204.23
135.29 128.99 128.61 128.04
77.32 77.00 76.68 76.22
48.33 48.14
21.54 18.73
O
H
NO2
14aa
210 200 190 180 170 160 150 140 130 120 110 100 90 80 70 60 50 40 30 20 10 0
f1 (ppm)

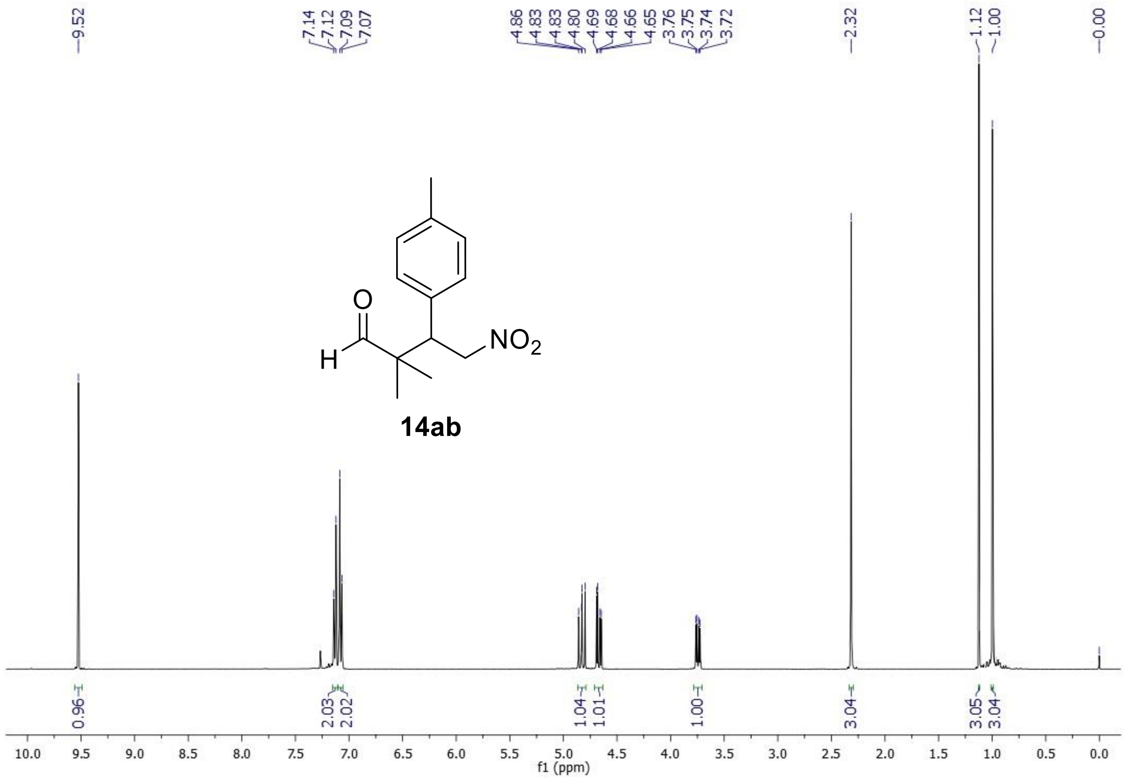
9.52
7.14
7.12
7.09
7.07
4.86
4.83
4.83
4.80
4.69
4.68
4.66
4.65
3.76
3.75
3.74
3.72
2.32
1.12
1.00
0.00
O
H
NO2
14ab
0.96
2.03
2.02
1.04
1.01
1.00
3.04
3.05
3.04
10.0 9.5 9.0 8.5 8.0 7.5 7.0 6.5 6.0 5.5 5.0 4.5 4.0 3.5 3.0 2.5 2.0 1.5 1.0 0.5 0.0
f1 (ppm)

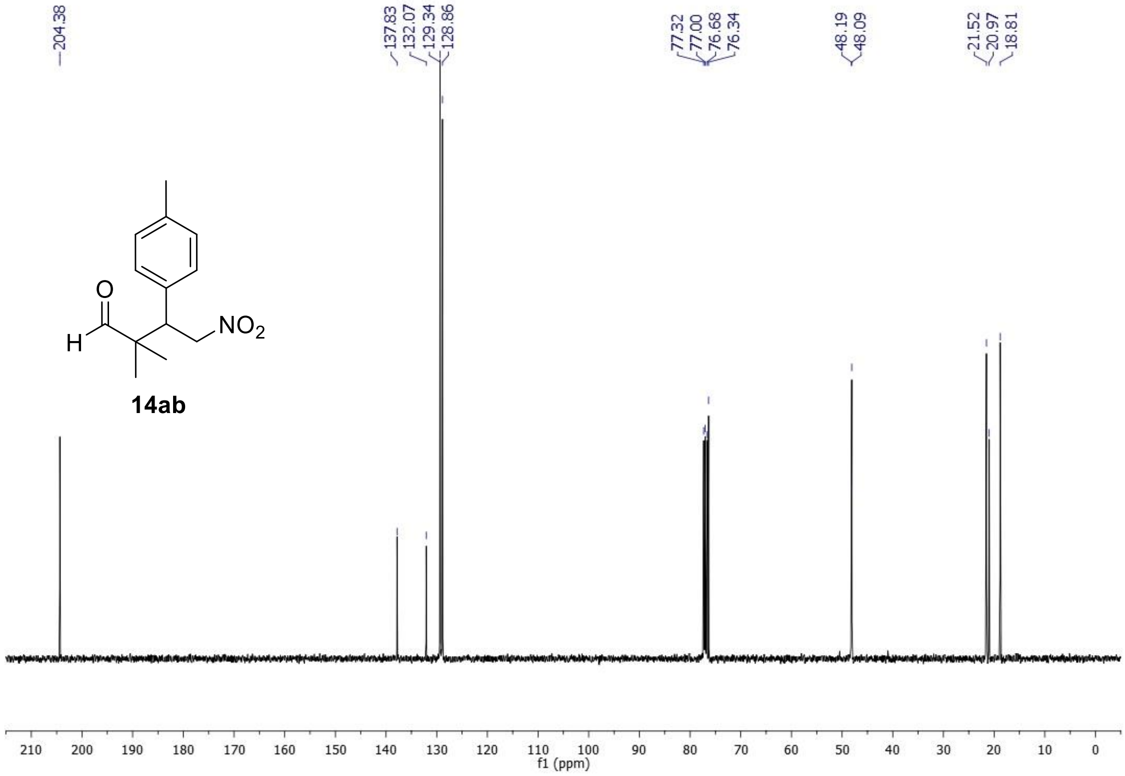
204.38
137.83
132.07
129.34
128.86
77.32
77.00
76.68
76.34
48.19
48.09
21.52
20.97
18.81
O
H
NO2
14ab
210 200 190 180 170 160 150 140 130 120 110 100 90 80 70 60 50 40 30 20 10 0
f1 (ppm)

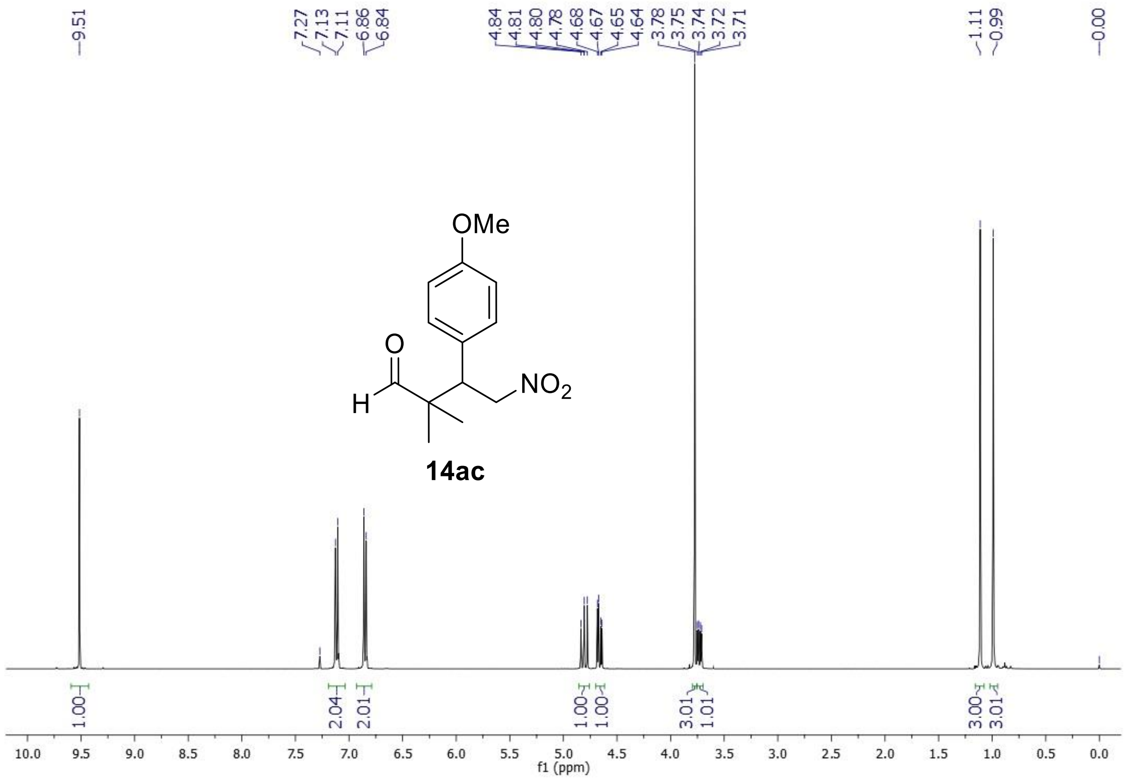
9.51
7.27
7.13
7.11
6.86
6.84
4.84
4.81
4.80
4.78
4.68
4.67
4.65
4.64
3.78
3.75
3.74
3.72
3.71
1.11
0.99
0.00
OMe
O
H
NO2
14ac
1.00
2.04
2.01
1.00
1.00
3.01
1.01
3.00
3.01
10.0 9.5 9.0 8.5 8.0 7.5 7.0 6.5 6.0 5.5 5.0 4.5 4.0 3.5 3.0 2.5 2.0 1.5 1.0 0.5 0.0
f1 (ppm)

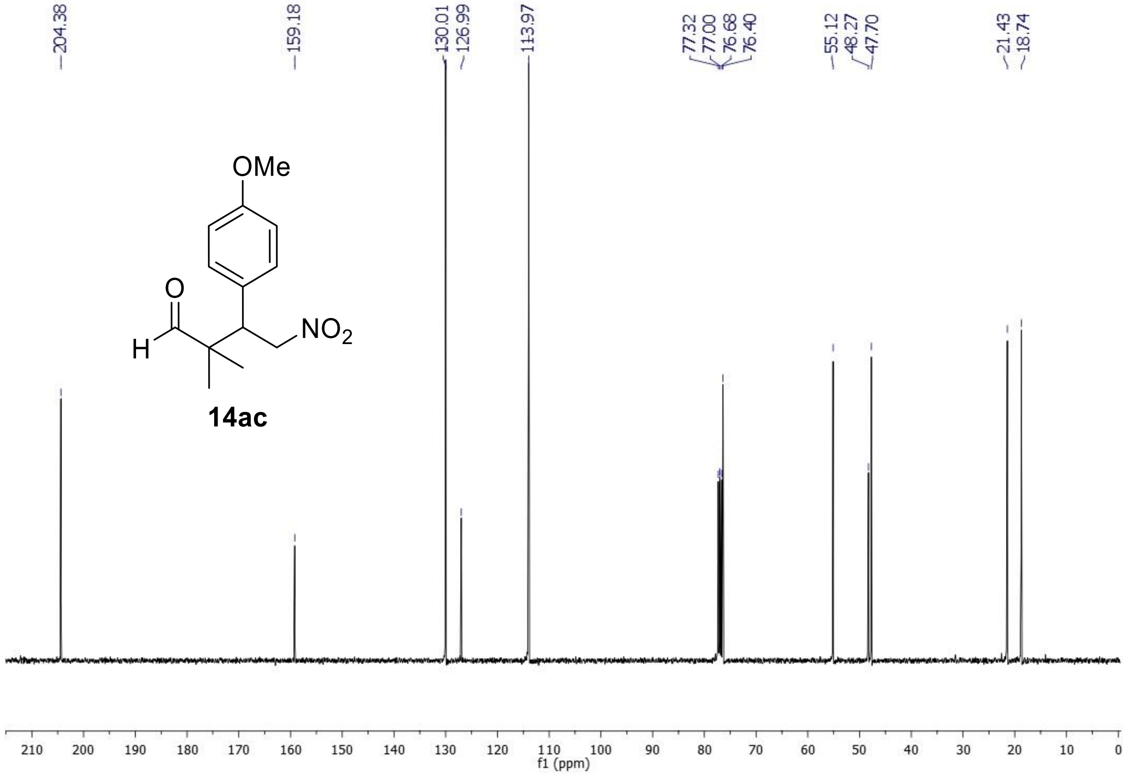
204.38
159.18
130.01
126.99
113.97
77.32
77.00
76.68
76.40
55.12
48.27
47.70
21.43
18.74
OMe
O
H
NO2
14ac
210 200 190 180 170 160 150 140 130 120 110 100 90 80 70 60 50 40 30 20 10 0
f1 (ppm)

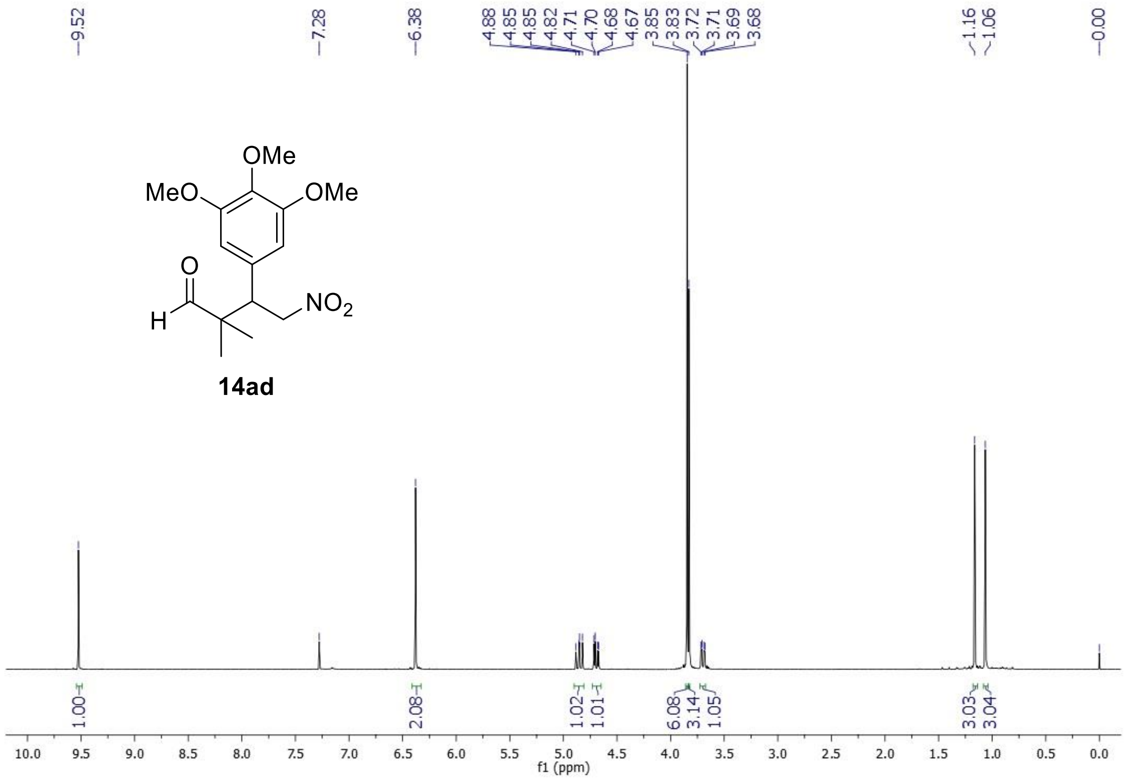
9.52
7.28
6.38
4.88
4.85
4.85
4.82
4.71
4.70
4.68
4.67
3.85
3.83
3.72
3.71
3.69
3.68
1.16
1.06
0.00
OMe
MeO
OMe
O
H
NO2
1.00
2.08
1.02
1.01
6.08
3.14
1.05
3.03
3.04
f1 (ppm)

14ad

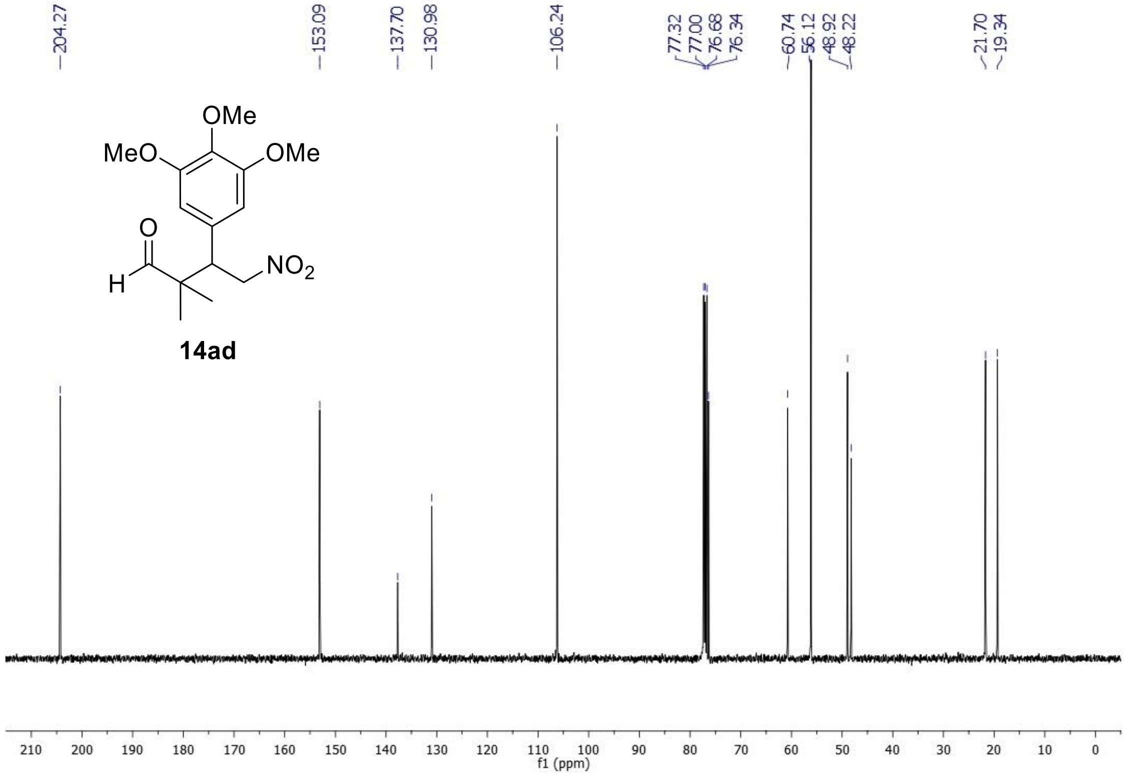
204.27
153.09
137.70
130.98
106.24
77.32
77.00
76.68
76.34
60.74
56.12
48.92
48.22
21.70
19.34
OMe
MeO
OMe
O
H
NO2
f1 (ppm)

14ad

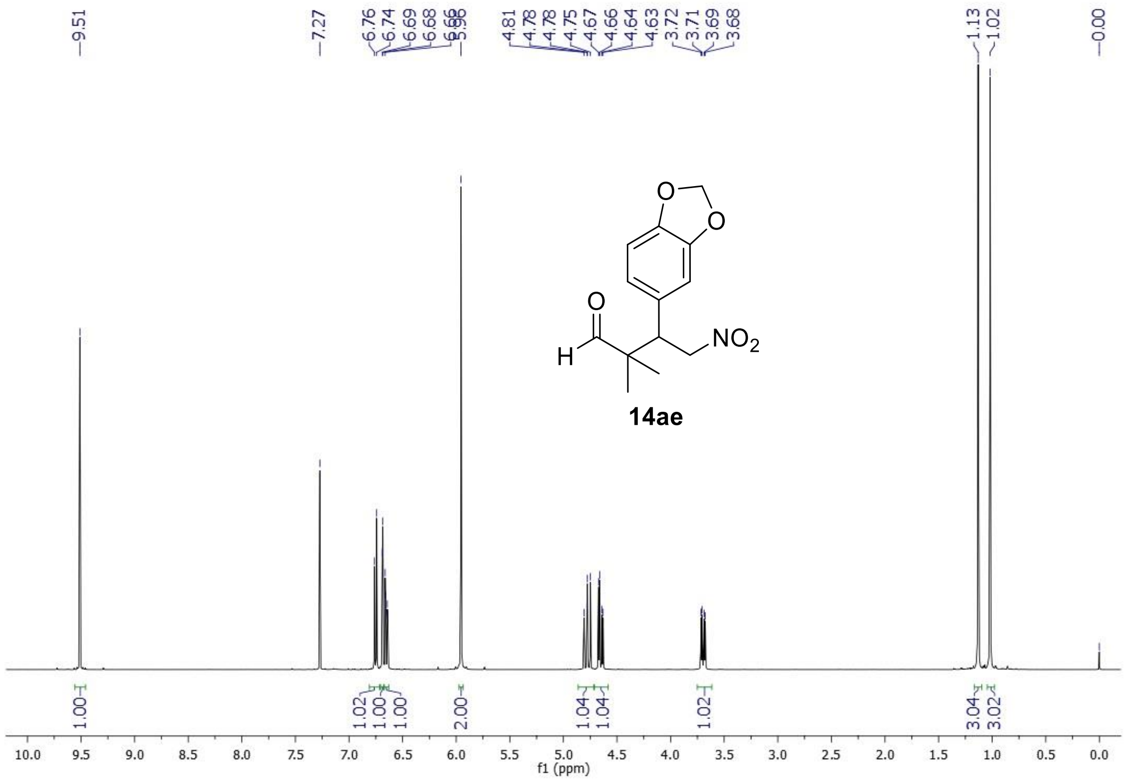
9.51
7.27
6.76
6.74
6.69
6.68
6.66
5.96
4.81
4.78
4.78
4.75
4.67
4.66
4.64
4.63
3.72
3.71
3.69
3.68
1.13
1.02
0.00
NO2
H
O
14ae
1.00
1.02
1.00
1.00
2.00
1.04
1.04
1.02
3.04
3.02
f1 (ppm)

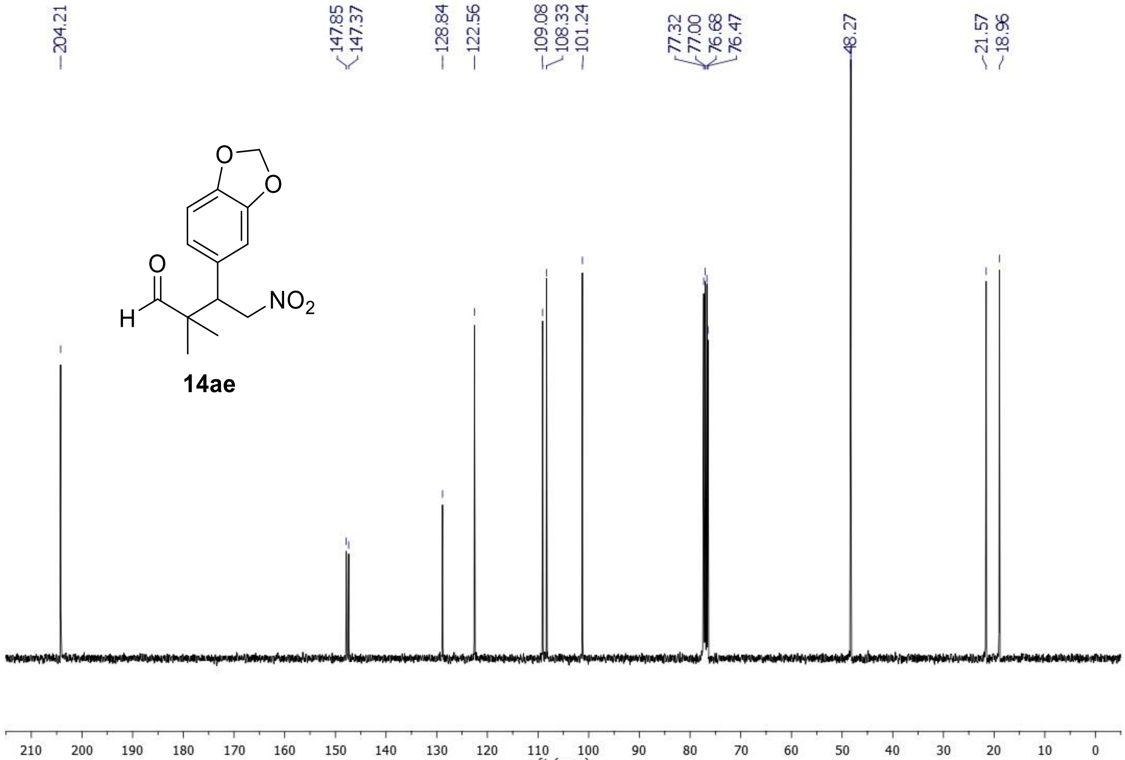
204.21
147.85
147.37
128.84
122.56
109.08
108.33
101.24
77.32
77.00
76.68
76.47
48.27
21.57
18.96
NO2
H
O
14ae
f1 (ppm)

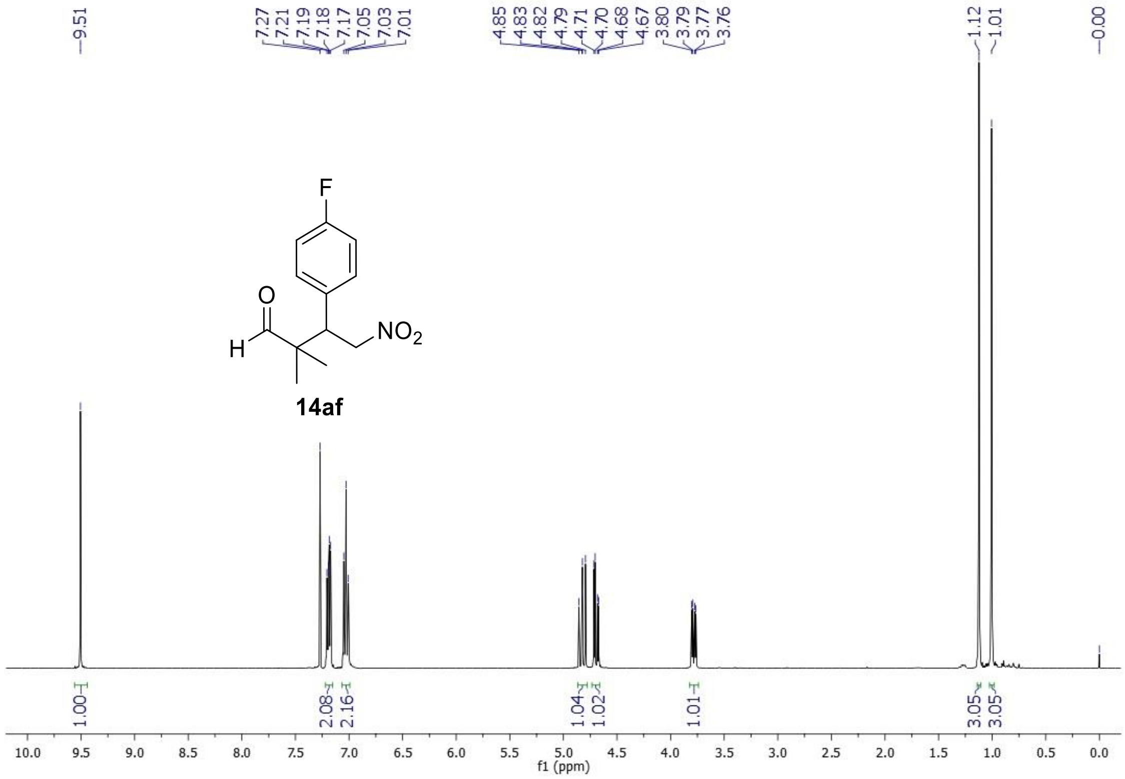

9.51
7.27
7.21
7.19
7.18
7.17
7.05
7.03
7.01
4.85
4.83
4.82
4.79
4.71
4.70
4.68
4.67
3.80
3.79
3.77
3.76
1.12
1.01
0.00
F
O
H
NO2
14af
1.00
2.08
2.16
1.04
1.02
1.01
3.05
3.05
10.0 9.5 9.0 8.5 8.0 7.5 7.0 6.5 6.0 5.5 5.0 4.5 4.0 3.5 3.0 2.5 2.0 1.5 1.0 0.5 0.0
f1 (ppm)

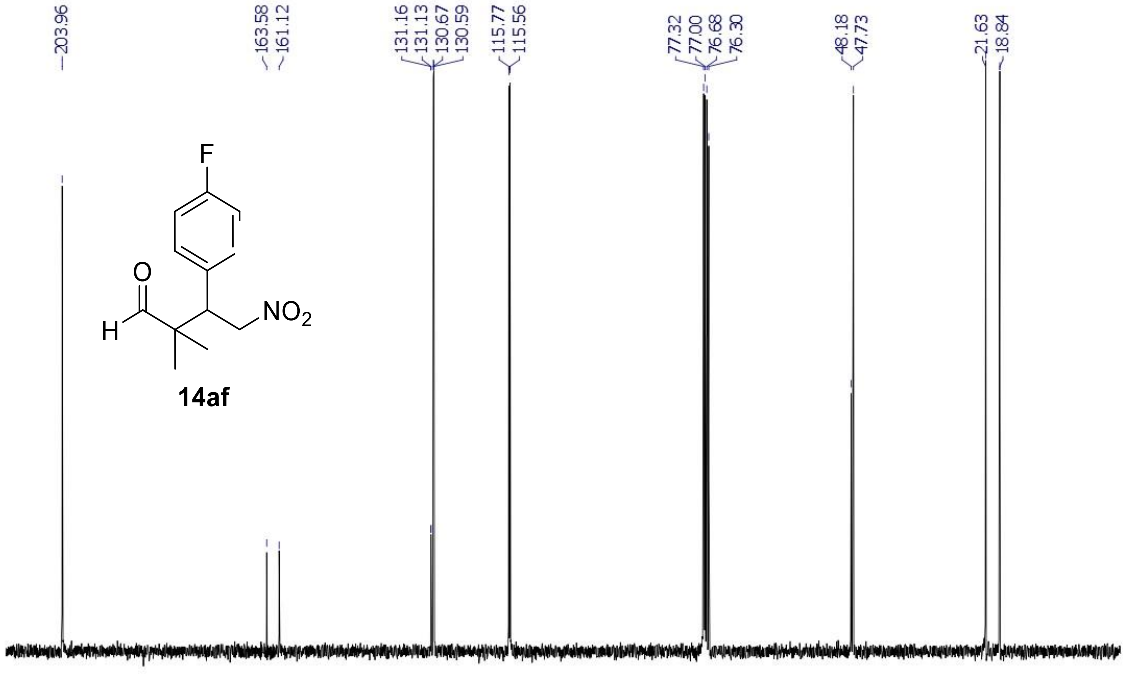

203.96
163.58
161.12
131.16
131.13
130.67
130.59
115.77
115.56
77.32
77.00
76.68
76.30
48.18
47.73
21.63
18.84
F
O
H
NO2
14af
210 200 190 180 170 160 150 140 130 120 110 100 90 80 70 60 50 40 30 20 10 0
f1 (ppm)

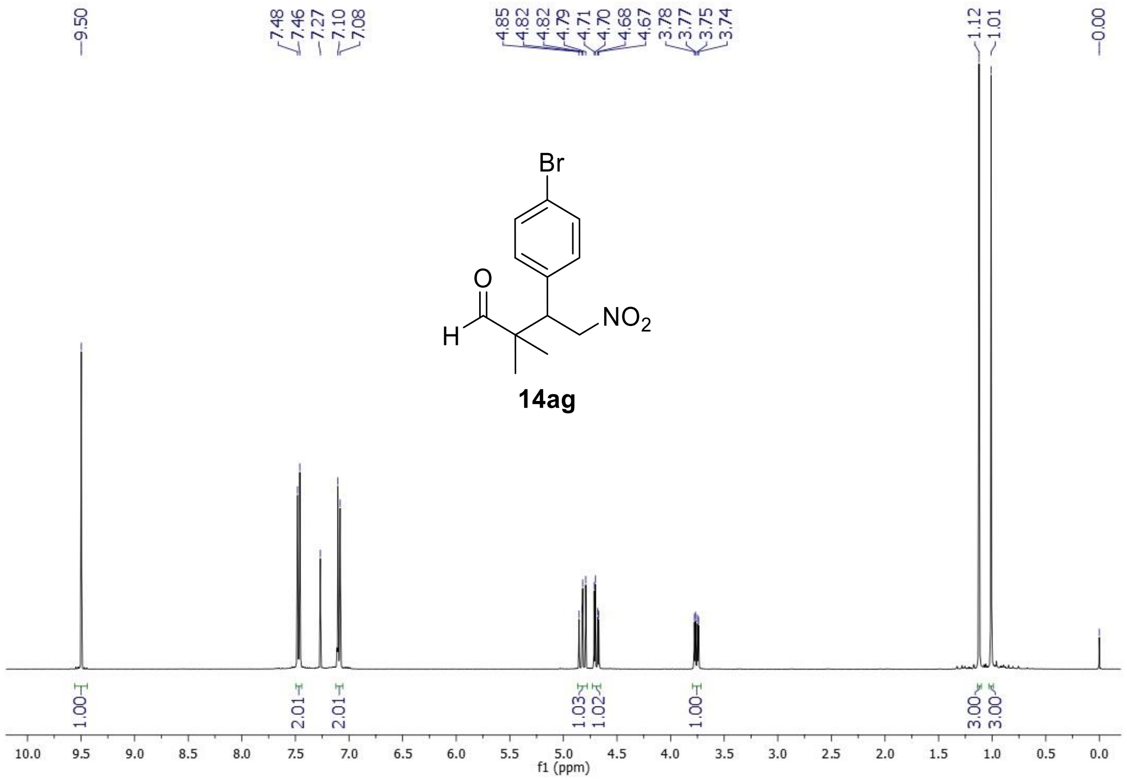
9.50
7.48
7.46
7.27
7.10
7.08
4.85
4.82
4.82
4.79
4.71
4.70
4.68
4.67
3.78
3.77
3.75
3.74
1.12
1.01
0.00
Br
O
H
NO2
14ag
1.00
2.01
2.01
1.03
1.02
1.00
3.00
3.00
10.0 9.5 9.0 8.5 8.0 7.5 7.0 6.5 6.0 5.5 5.0 4.5 4.0 3.5 3.0 2.5 2.0 1.5 1.0 0.5 0.0
f1 (ppm)

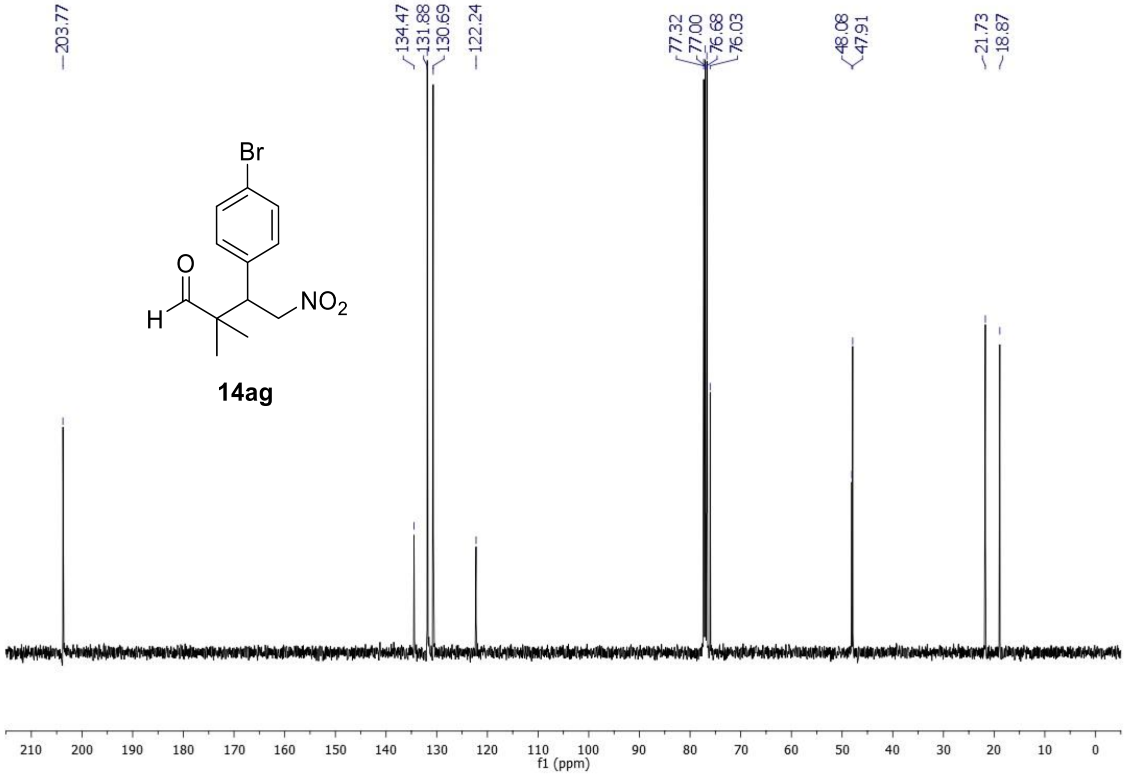
203.77
134.47
131.88
130.69
122.24
77.32
77.00
76.68
76.03
48.08
47.91
21.73
18.87
Br
O
H
NO2
14ag
210 200 190 180 170 160 150 140 130 120 110 100 90 80 70 60 50 40 30 20 10 0
f1 (ppm)

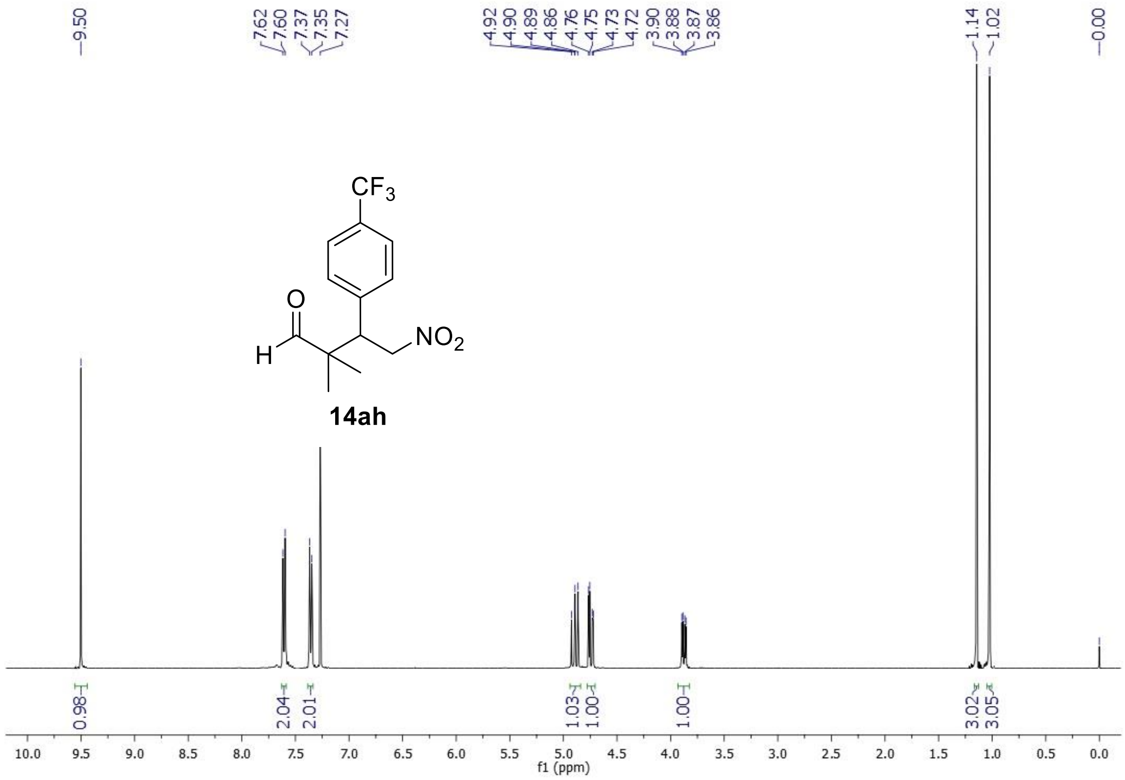
9.50
7.62
7.60
7.37
7.35
7.27
4.92
4.90
4.89
4.86
4.76
4.75
4.73
4.72
3.90
3.88
3.87
3.86
1.14
1.02
0.00
CF3
O
H
NO2
14ah
0.98
2.04
2.01
1.03
1.00
1.00
3.02
3.05
f1 (ppm)

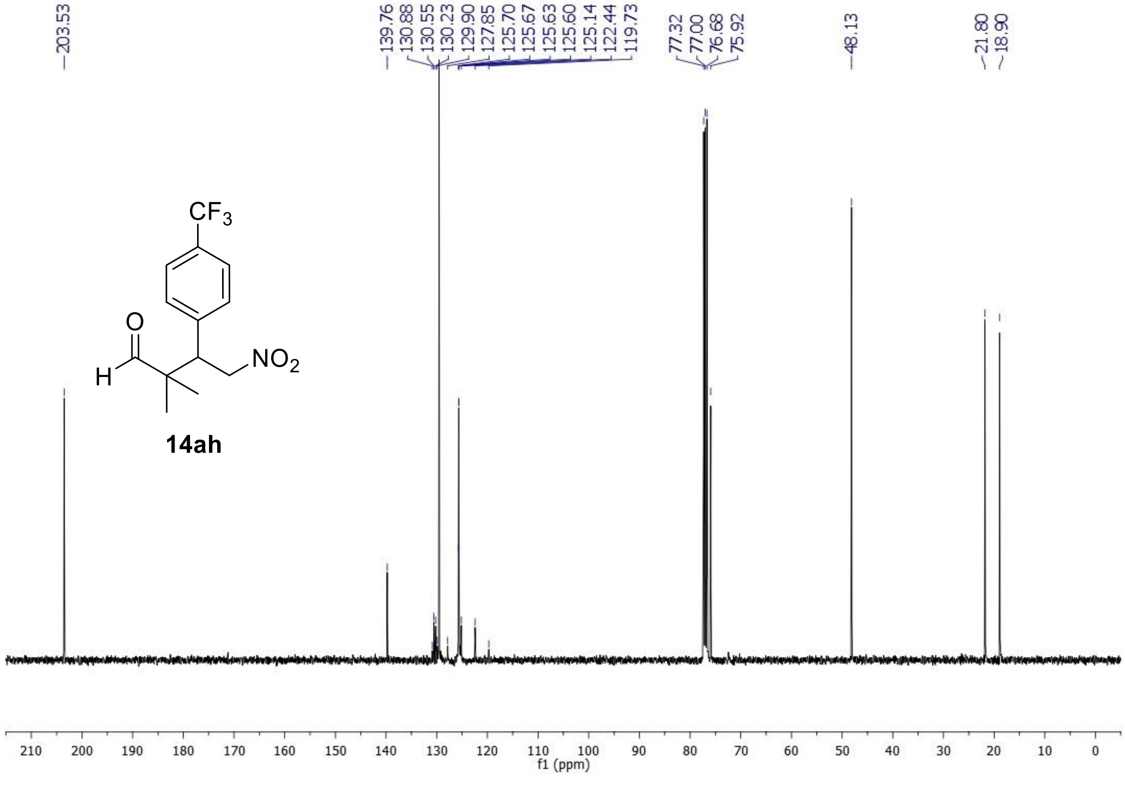
203.53
139.76
130.88
130.55
130.23
129.90
127.85
125.70
125.67
125.63
125.60
125.14
122.44
119.73
77.32
77.00
76.68
75.92
48.13
21.80
18.90
CF3
O
H
NO2
14ah
f1 (ppm)

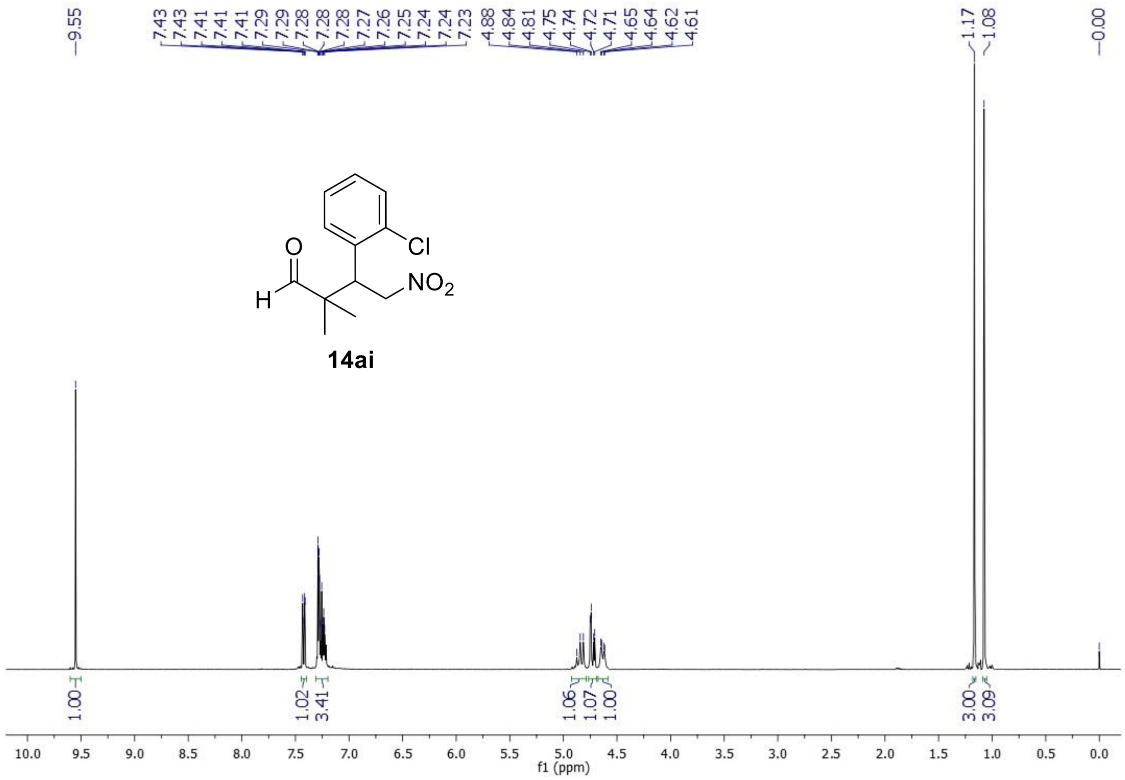
9.55
7.43
7.43
7.41
7.41
7.41
7.29
7.29
7.28
7.28
7.28
7.27
7.26
7.25
7.24
7.24
7.23
4.88
4.84
4.81
4.75
4.74
4.72
4.71
4.65
4.64
4.62
4.61
1.17
1.08
0.00
O
H
Cl
NO2
14ai
1.00
1.02
3.41
1.06
1.07
1.00
3.00
3.09
10.0 9.5 9.0 8.5 8.0 7.5 7.0 6.5 6.0 5.5 5.0 4.5 4.0 3.5 3.0 2.5 2.0 1.5 1.0 0.5 0.0
f1 (ppm)

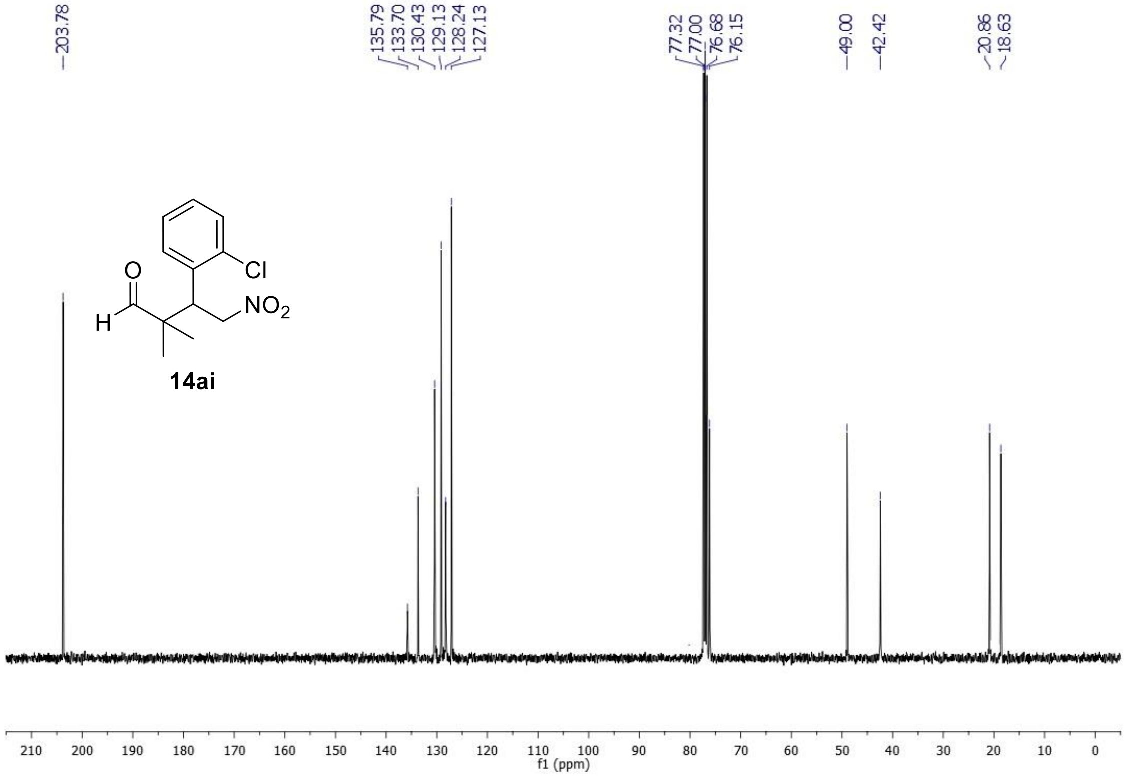
203.78
135.79
133.70
130.43
129.13
128.24
127.13
77.32
77.00
76.68
76.15
49.00
42.42
20.86
18.63
O
H
Cl
NO2
14ai
210 200 190 180 170 160 150 140 130 120 110 100 90 80 70 60 50 40 30 20 10 0
f1 (ppm)

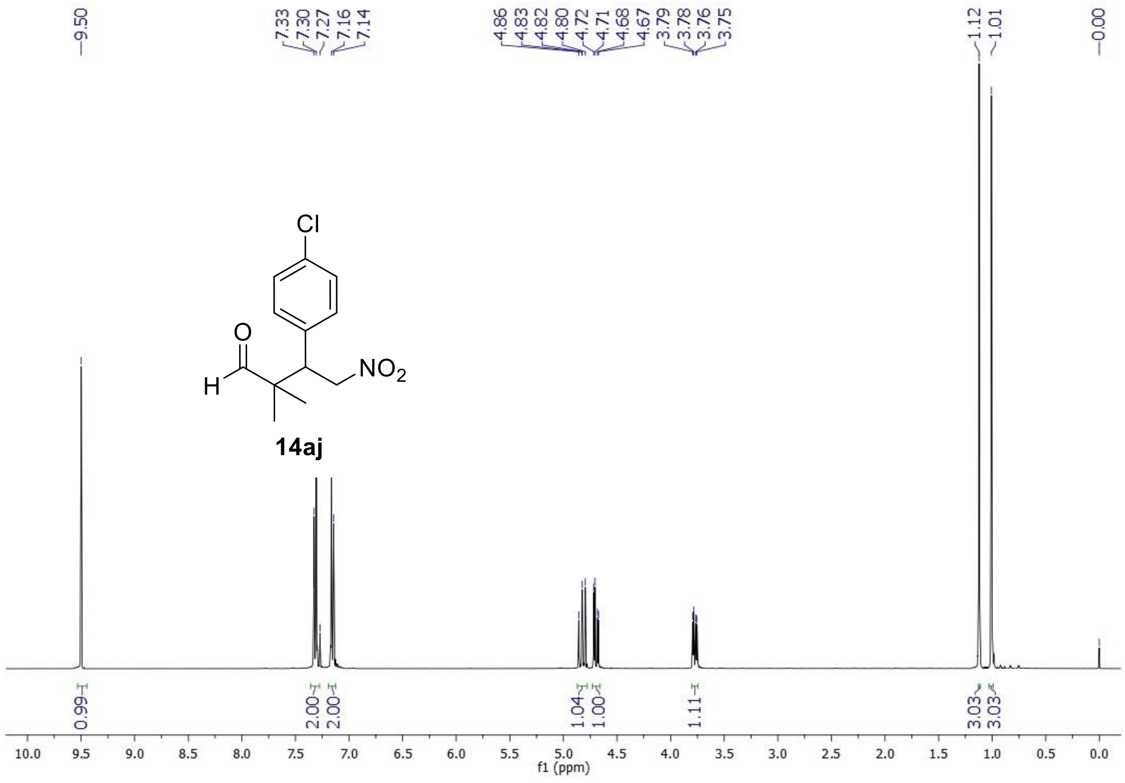
9.50
7.33
7.30
7.27
7.16
7.14
4.86
4.83
4.82
4.80
4.72
4.71
4.68
4.67
3.79
3.78
3.76
3.75
1.12
1.01
0.00
Cl
O
H
NO2
14aj
0.99
2.00
2.00
1.04
1.00
1.11
3.03
3.03
10.0 9.5 9.0 8.5 8.0 7.5 7.0 6.5 6.0 5.5 5.0 4.5 4.0 3.5 3.0 2.5 2.0 1.5 1.0 0.5 0.0
f1 (ppm)

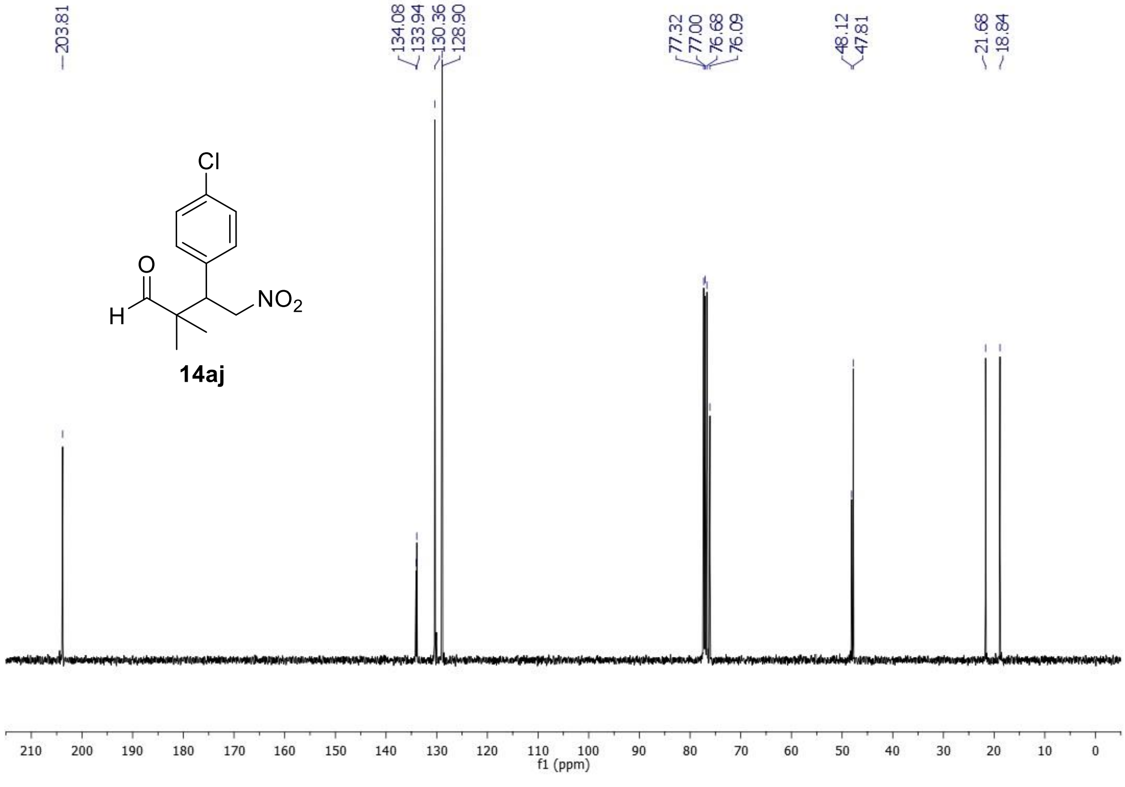
203.81
134.08
133.94
130.36
128.90
77.32
77.00
76.68
76.09
48.12
47.81
21.68
18.84
Cl
O
H
NO2
14aj
210 200 190 180 170 160 150 140 130 120 110 100 90 80 70 60 50 40 30 20 10 0
f1 (ppm)

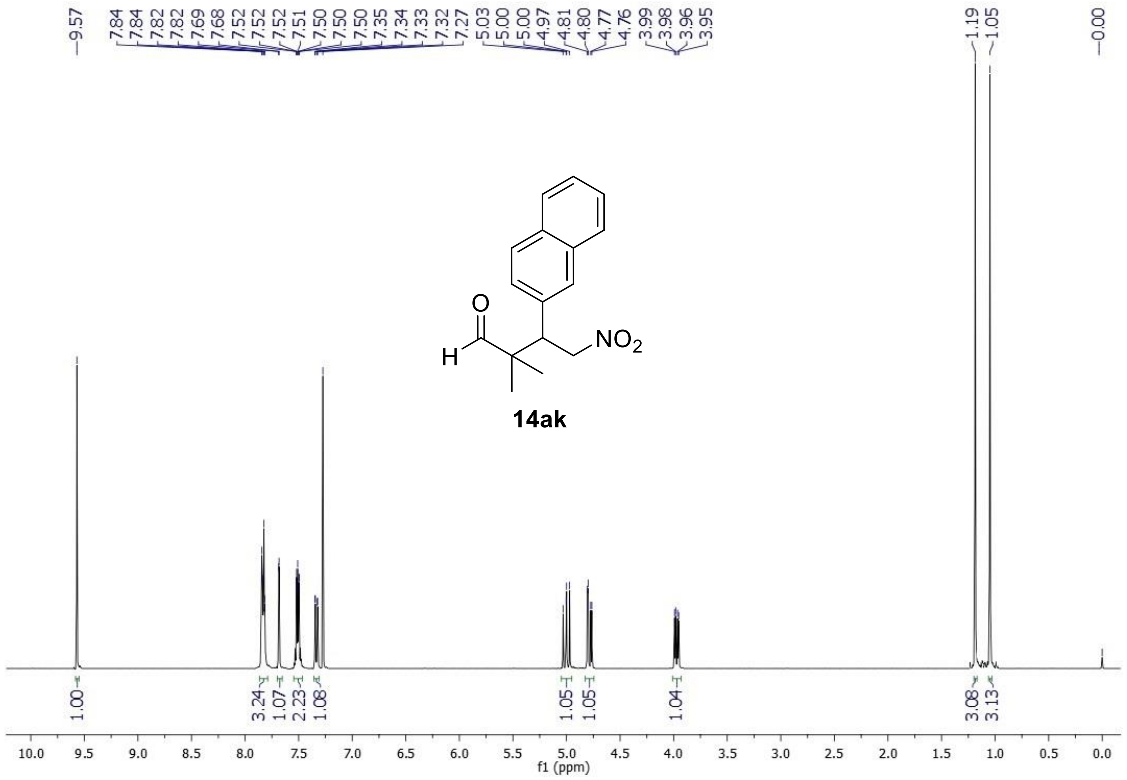
9.57
7.84 7.84 7.82 7.82 7.69 7.68 7.52 7.52 7.52 7.51 7.50 7.50 7.50 7.35 7.34 7.33 7.32 7.27
5.03 5.00 5.00 4.97 4.81 4.80 4.77 4.76
3.99 3.98 3.96 3.95
1.19 1.05
0.00
O
H
NO2
14ak
1.00
3.24 1.07 2.23 1.08
1.05 1.05
1.04
3.08 3.13
10.0 9.5 9.0 8.5 8.0 7.5 7.0 6.5 6.0 5.5 5.0 4.5 4.0 3.5 3.0 2.5 2.0 1.5 1.0 0.5 0.0
f1 (ppm)

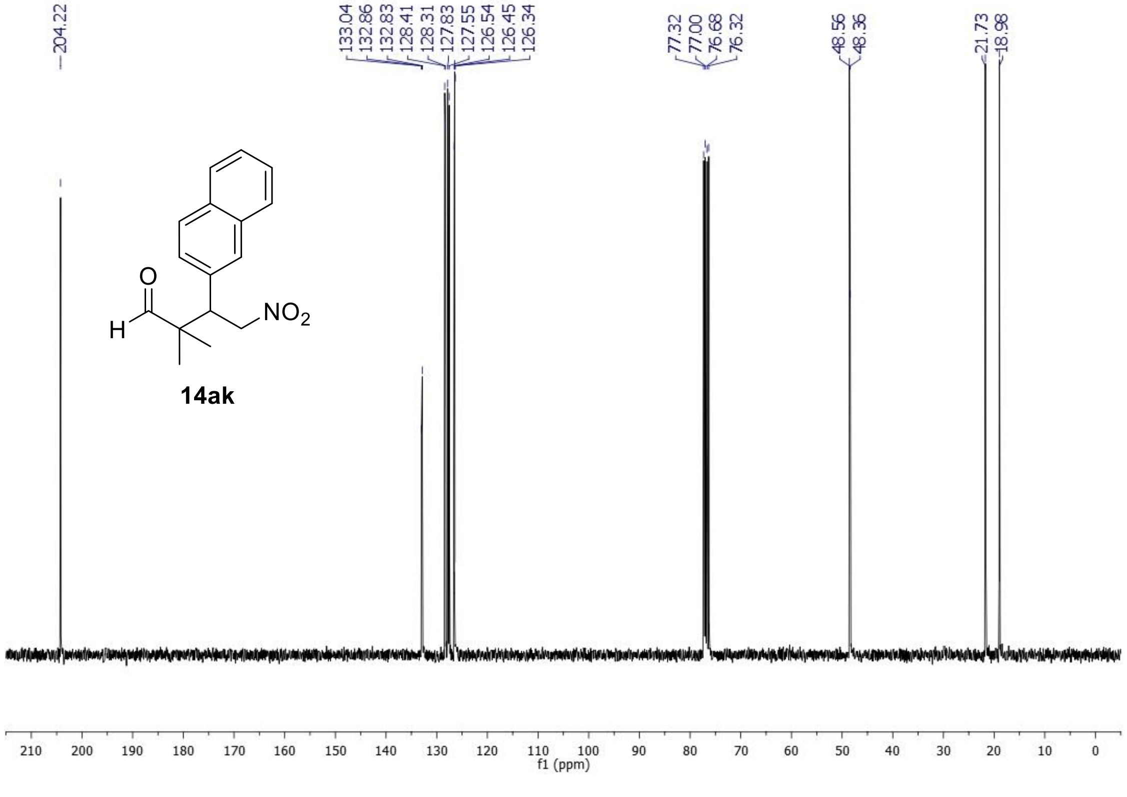
204.22
133.04 132.86 132.83 128.41 128.31 127.83 127.55 126.54 126.45 126.34
77.32 77.00 76.68 76.32
48.56 48.36
21.73 18.98
O
H
NO2
14ak
210 200 190 180 170 160 150 140 130 120 110 100 90 80 70 60 50 40 30 20 10 0
f1 (ppm)

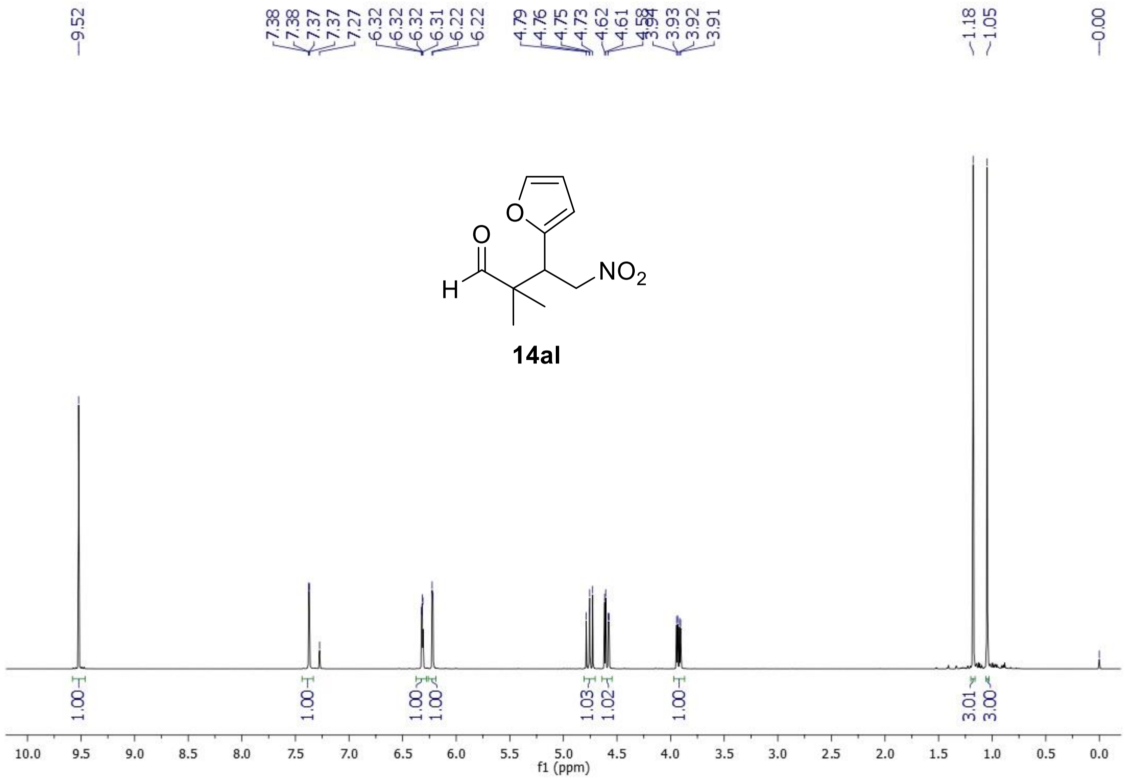
9.52
7.38
7.38
7.37
7.37
7.27
6.32
6.32
6.32
6.31
6.22
6.22
4.79
4.76
4.75
4.73
4.62
4.61
4.58
3.94
3.93
3.92
3.91
1.18
1.05
0.00
O
O
H
NO2
14al
1.00
1.00
1.00
1.00
1.03
1.02
1.00
3.01
3.00
f1 (ppm)

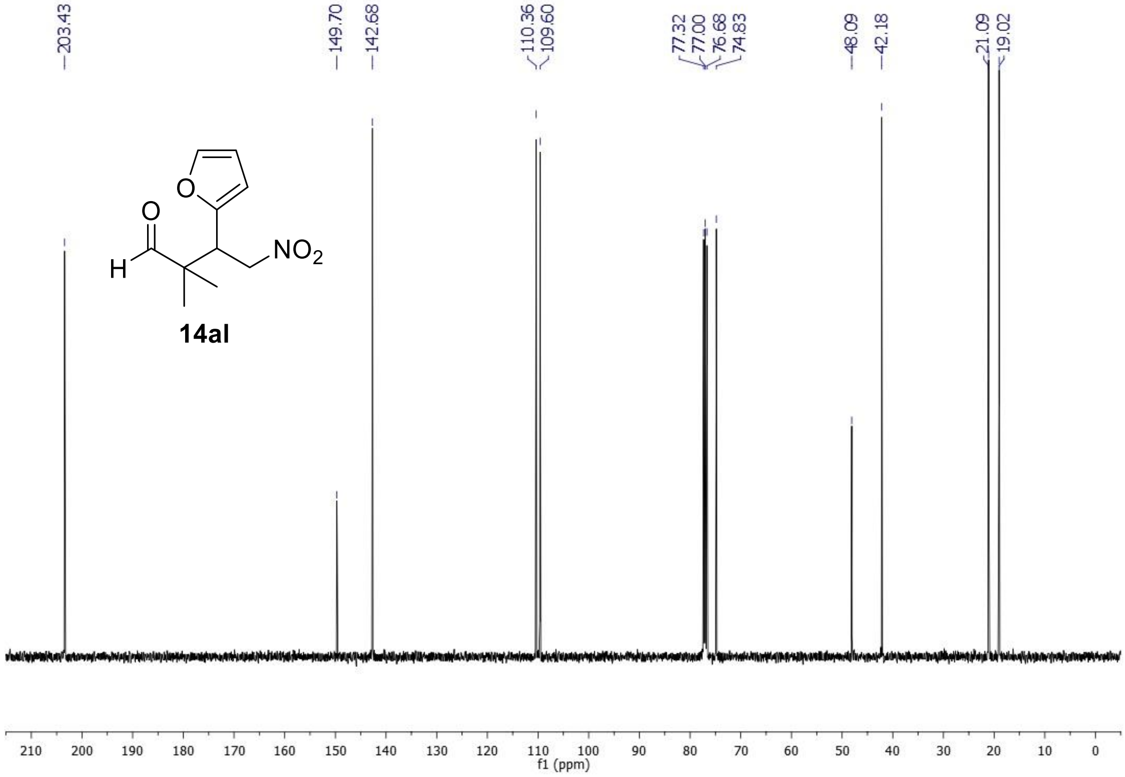
203.43
149.70
142.68
110.36
109.60
77.32
77.00
76.68
74.83
48.09
42.18
21.09
19.02
O
O
H
NO2
14al
f1 (ppm)

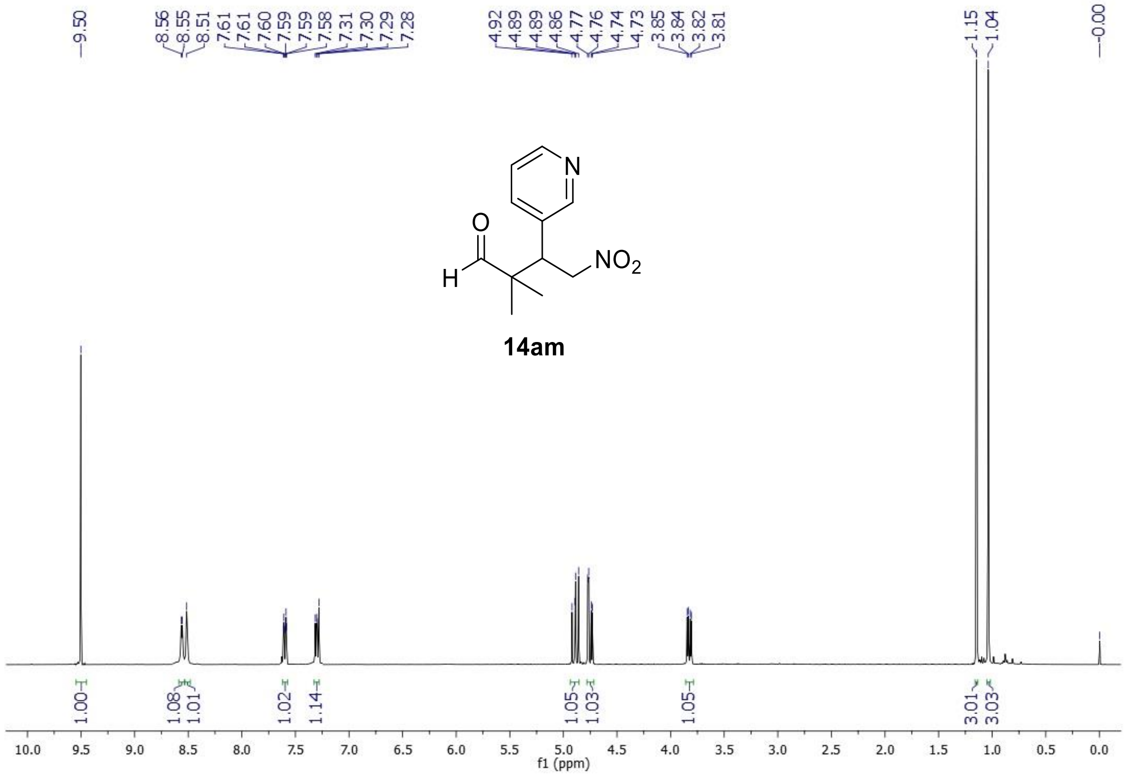
9.50
8.56 8.55 8.51 7.61 7.61 7.60 7.59 7.59 7.58 7.31 7.30 7.29 7.28
4.92 4.89 4.89 4.86 4.77 4.76 4.74 4.73 3.85 3.84 3.82 3.81
1.15 1.04
0.00
O
H
N
NO_2
14am
1.00 1.08 1.01 1.02 1.14 1.05 1.03 1.05 3.01 3.03
10.0 9.5 9.0 8.5 8.0 7.5 7.0 6.5 6.0 5.5 5.0 4.5 4.0 3.5 3.0 2.5 2.0 1.5 1.0 0.5 0.0
f1 (ppm)

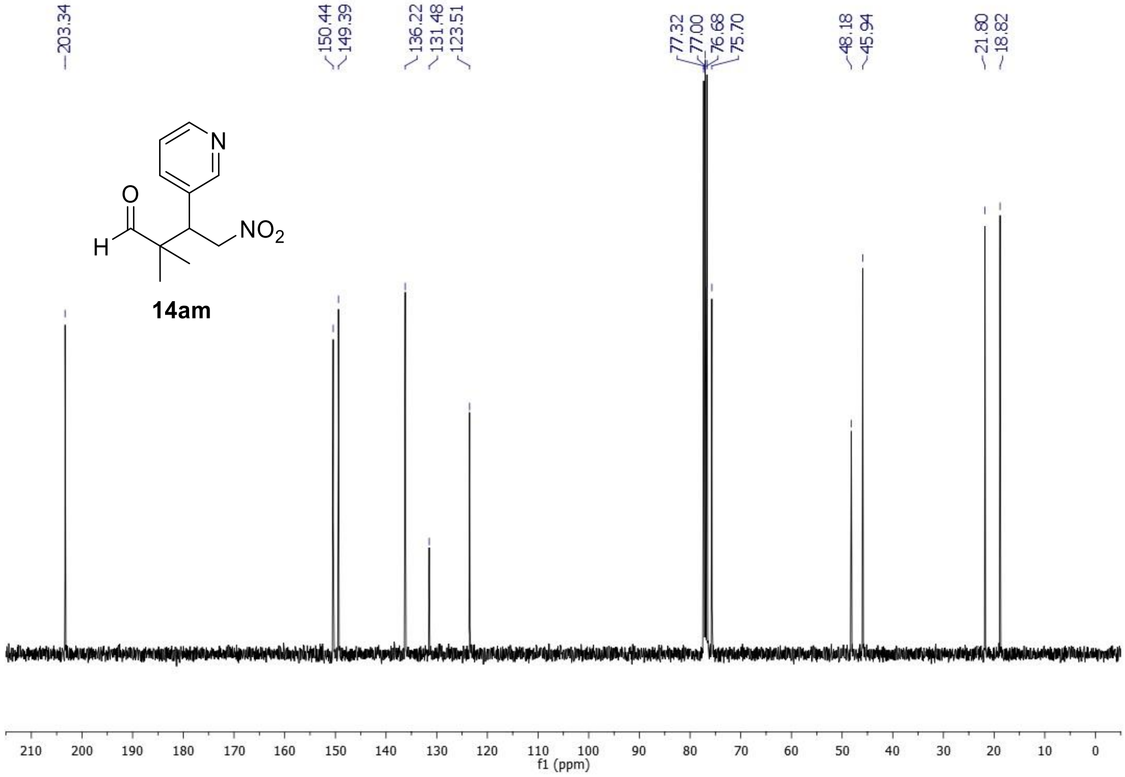
203.34
150.44 149.39
136.22 131.48 123.51
77.32 77.00 76.68 75.70
48.18 45.94
21.80 18.82
O
H
N
NO_2
14am
210 200 190 180 170 160 150 140 130 120 110 100 90 80 70 60 50 40 30 20 10 0
f1 (ppm)

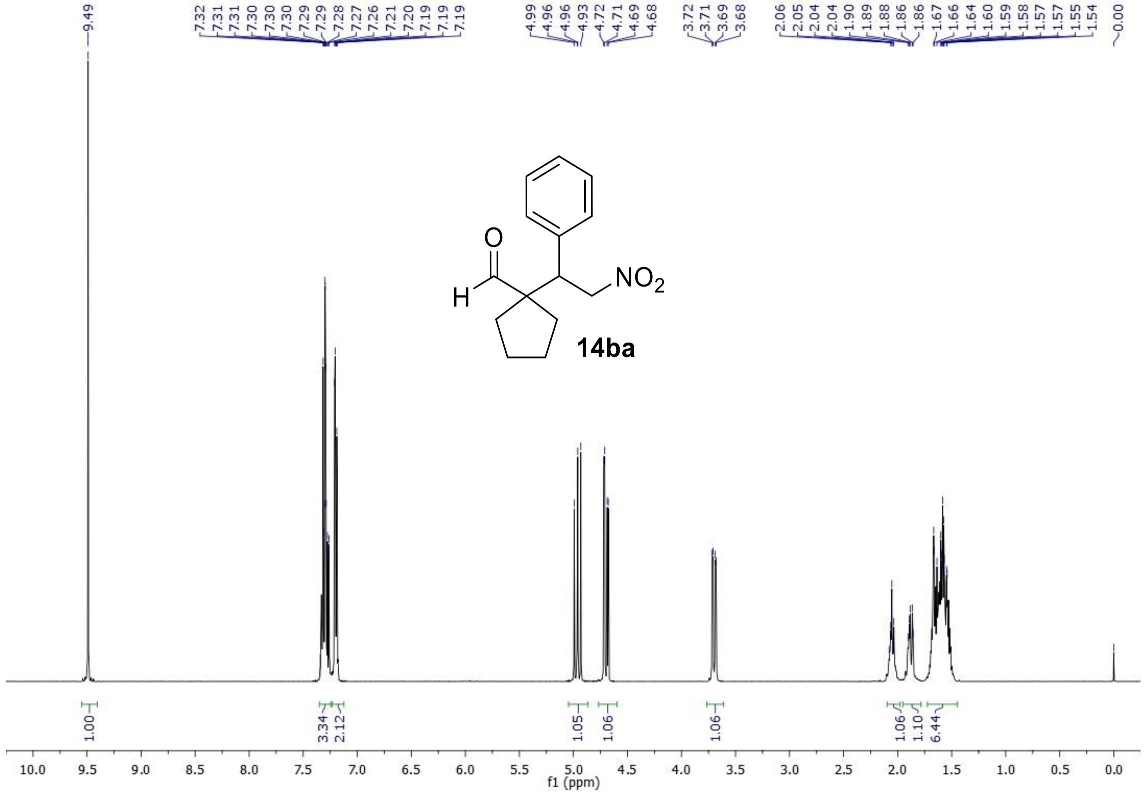
9.49
7.32 7.31 7.31 7.30 7.30 7.30 7.29 7.29 7.28 7.27 7.26 7.21 7.20 7.19 7.19 7.19
4.99 4.96 4.96 4.93 4.72 4.71 4.69 4.68
3.72 3.71 3.69 3.68
2.06 2.05 2.04 2.04 1.90 1.89 1.88 1.86 1.86 1.67 1.66 1.64 1.60 1.59 1.58 1.57 1.55 1.54
0.00
O
H
NO2
14ba
1.00
3.34
2.12
1.05
1.06
1.06
1.06
1.10
6.44
f1 (ppm)

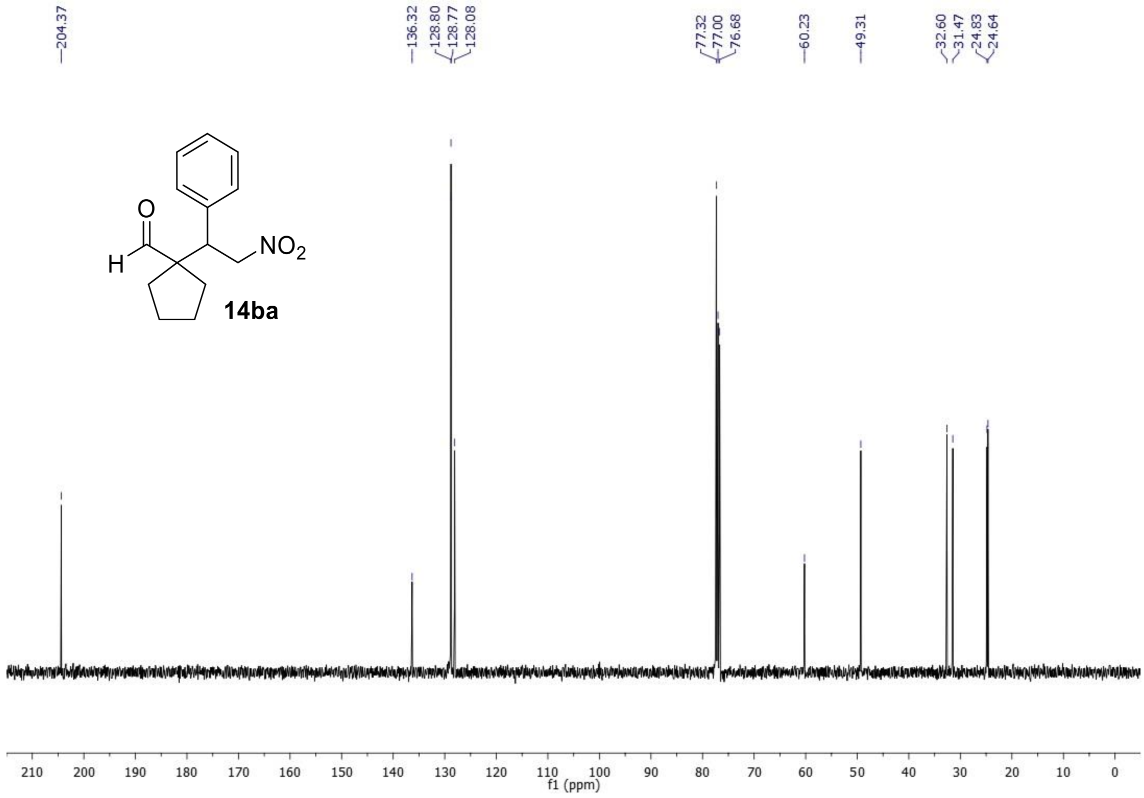
204.37
136.32 128.80 128.77 128.08
77.32 77.00 76.68
60.23
49.31
32.60 31.47 24.83 24.64
O
H
NO2
14ba
f1 (ppm)

HPLC Chromatograms

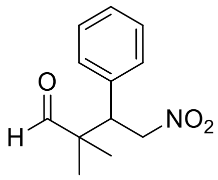

14aa

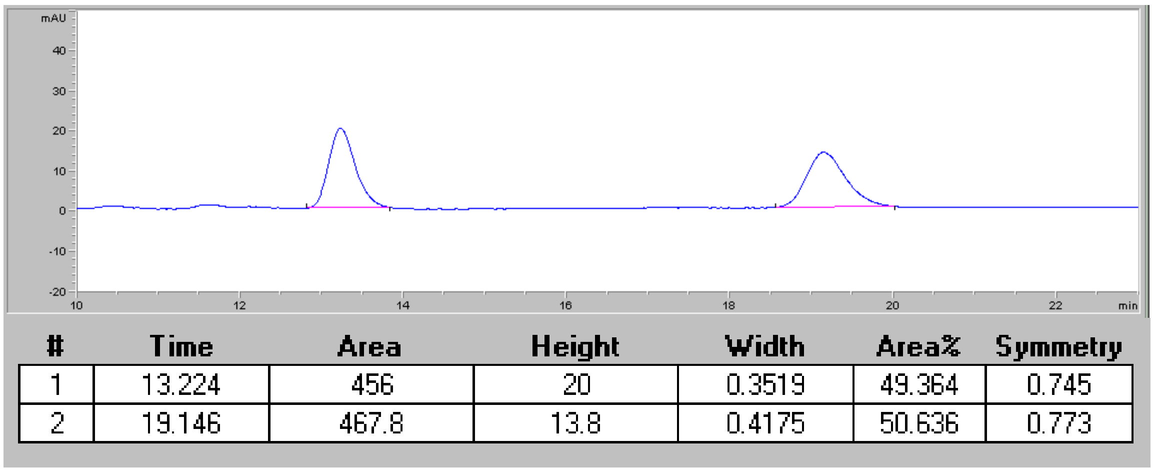

#	Time	Area	Height	Width	Area%	Symmetry
1	13.224	456	20	0.3519	49.364	0.745
2	19.146	467.8	13.8	0.4175	50.636	0.773

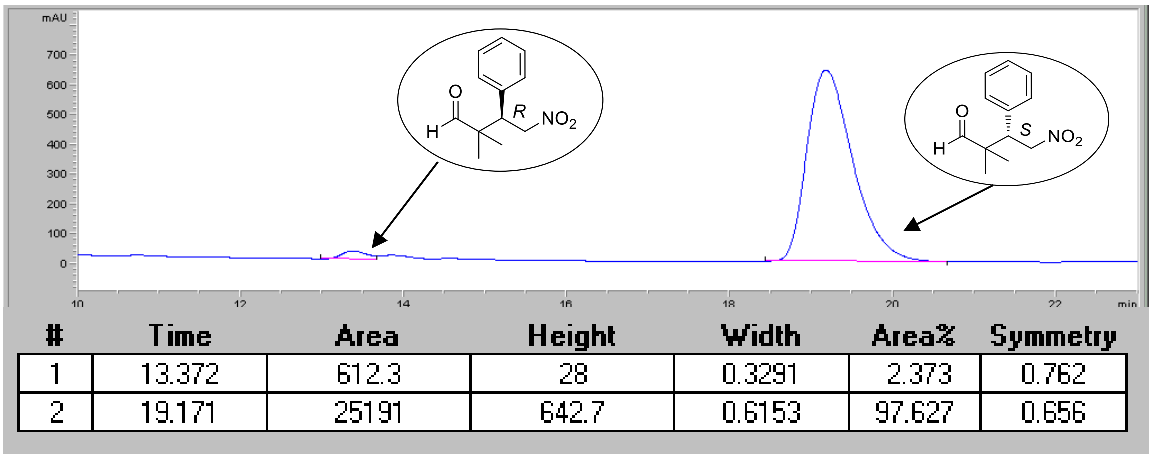

#	Time	Area	Height	Width	Area%	Symmetry
1	13.372	612.3	28	0.3291	2.373	0.762
2	19.171	25191	642.7	0.6153	97.627	0.656

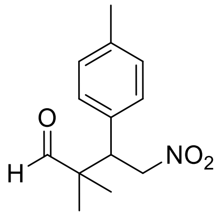

14ab

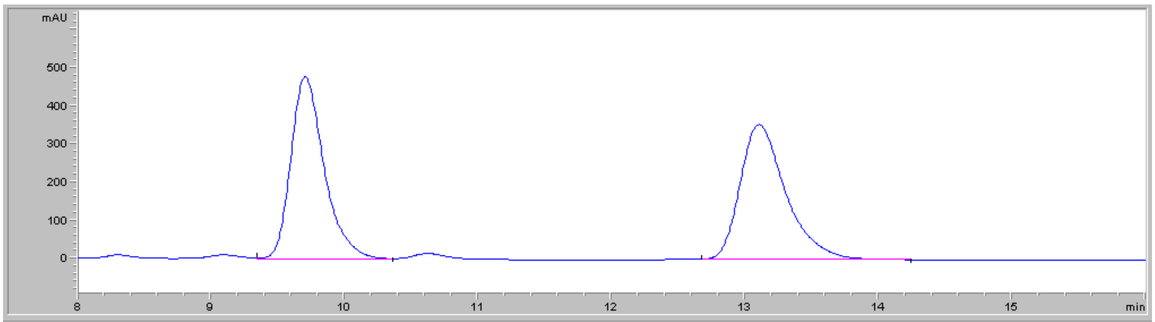

#	Time	Area	Height	Width	Area%	Symmetry
1	9.701	8434.7	480.5	0.2668	49.935	0.713
2	13.1	8456.6	354.3	0.3624	50.065	0.658

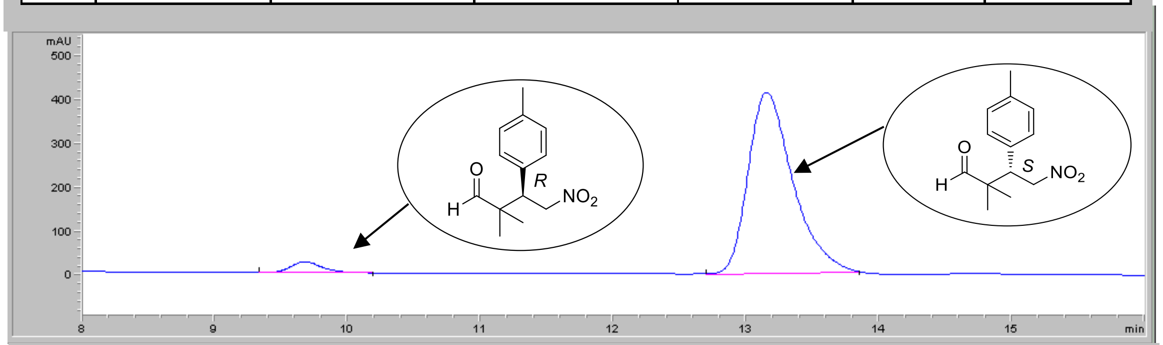

#	Time	Area	Height	Width	Area%	Symmetry
1	9.676	397	24.9	0.2661	3.895	0.781
2	13.151	9797.3	414.3	0.3941	96.105	0.684

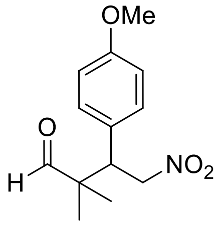

14ac

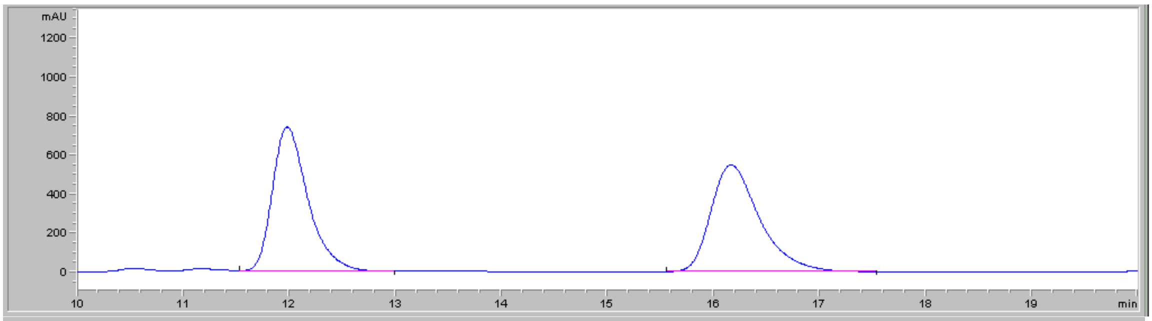

#	Time	Area	Height	Width	Area%	Symmetry
1	11.977	17301.2	740.9	0.3564	49.741	0.682
2	16.161	17481.1	548.3	0.484	50.259	0.659

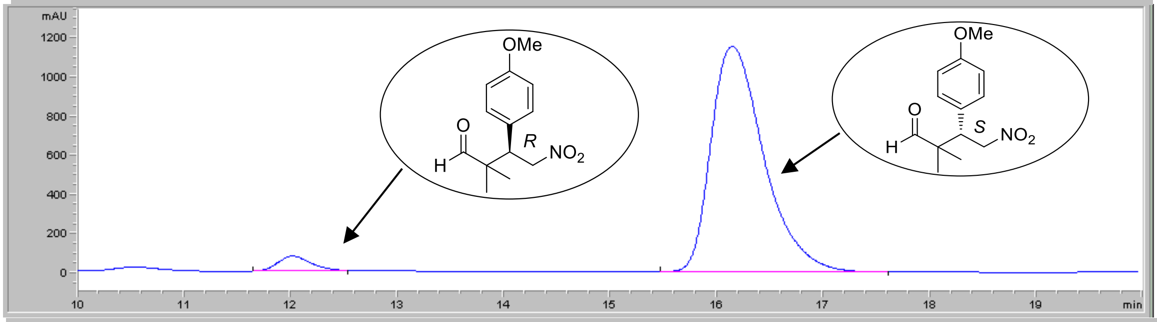

#	Time	Area	Height	Width	Area%	Symmetry
1	12.015	1625.1	75.4	0.3593	3.932	0.757
2	16.143	39701.9	1151.7	0.5322	96.068	0.631

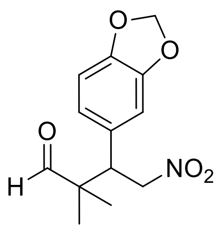

14ad

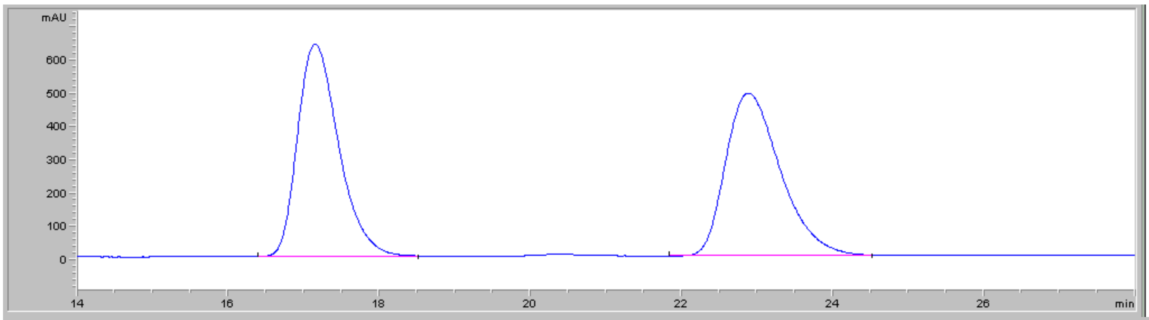

#	Time	Area	Height	Width	Area%	Symmetry
1	17.142	24292.9	640.3	0.5586	49.467	0.691
2	22.888	24816.1	489.4	0.7135	50.533	0.692

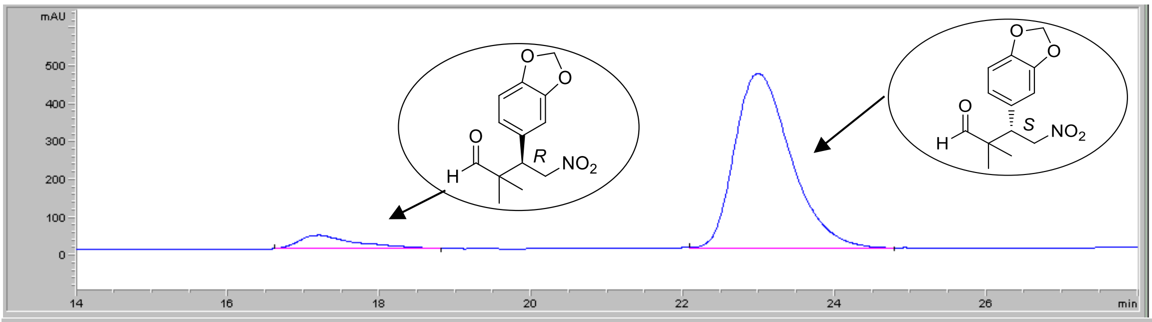

#	Time	Area	Height	Width	Area%	Symmetry
1	17.206	1898.8	35.8	0.8839	7.297	0.536
2	22.988	24123.3	463.7	0.655	92.703	0.696

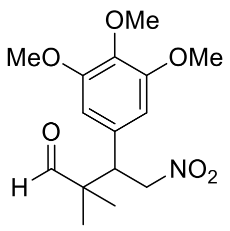

14ae

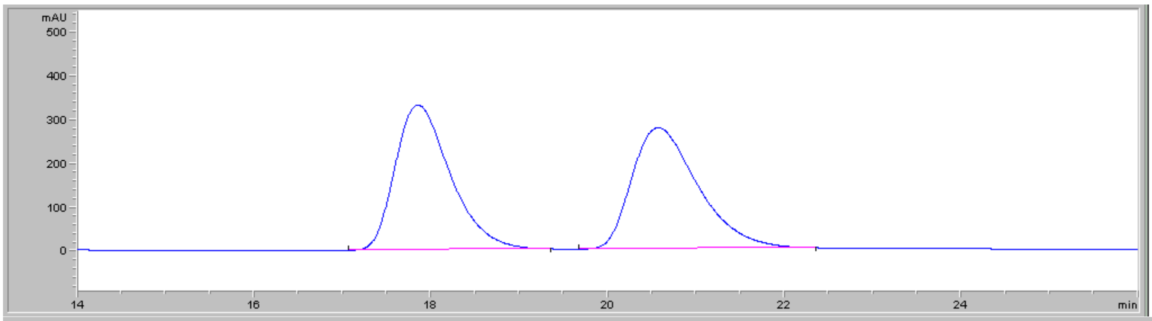

#	Time	Area	Height	Width	Area%	Symmetry
1	17.85	14376.5	331	0.6587	49.906	0.664
2	20.567	14430.8	276.7	0.7984	50.094	0.617

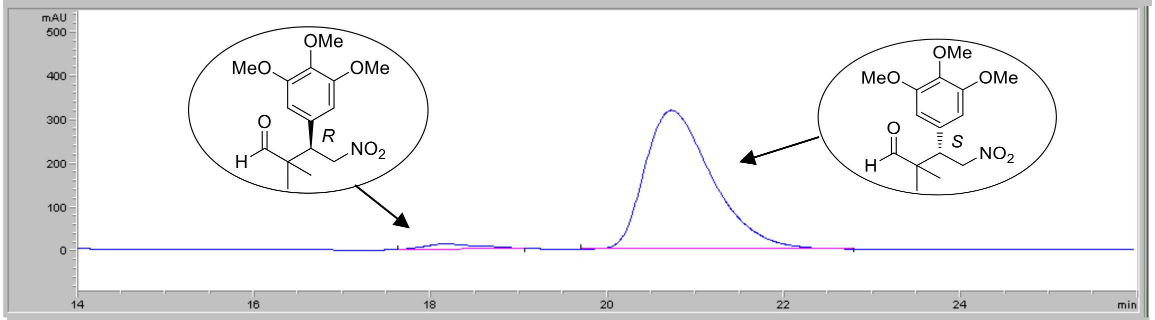

#	Time	Area	Height	Width	Area%	Symmetry
1	18.167	525.5	12	0.727	3.024	0.584
2	20.72	16853	317.5	0.8847	96.976	0.629

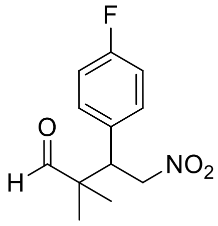

14af

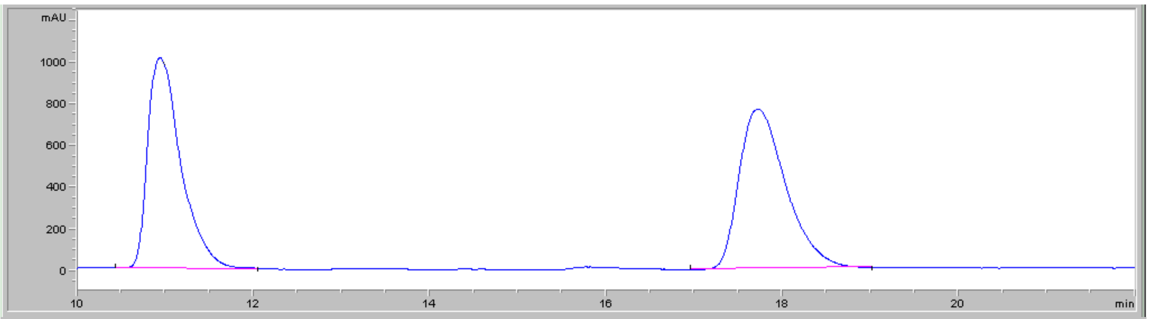

#	Time	Area	Height	Width	Area%	Symmetry
1	10.938	26541.1	1010.5	0.4377	48.379	0.566
2	17.722	28319.7	766.9	0.6155	51.621	0.66

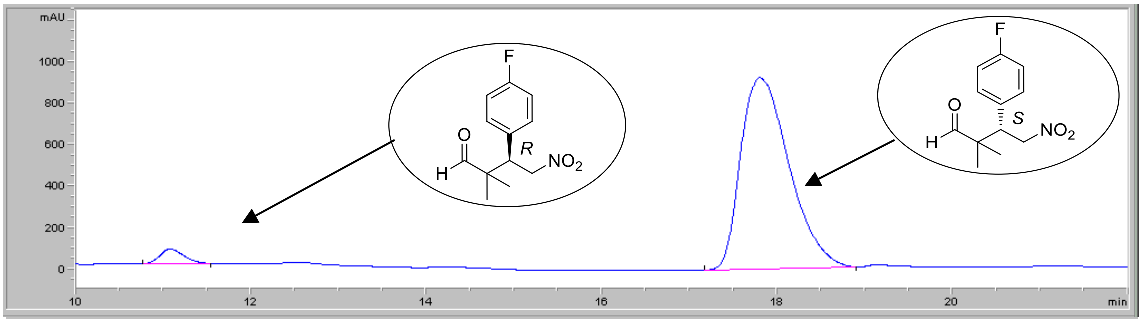

#	Time	Area	Height	Width	Area%	Symmetry
1	11.077	1440	72.7	0.3301	3.877	0.724
2	17.799	35699.6	926.2	0.6424	96.123	0.624

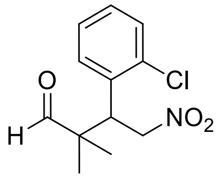

14ag

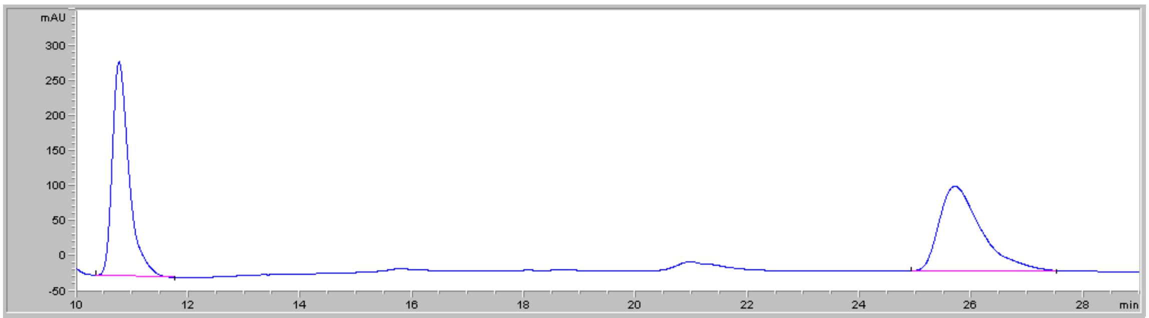

#	Time	Area	Height	Width	Area%	Symmetry
1	10.758	6324.2	305.9	0.3132	50.214	0.63
2	25.695	6270.2	120.7	0.7599	49.786	0.561

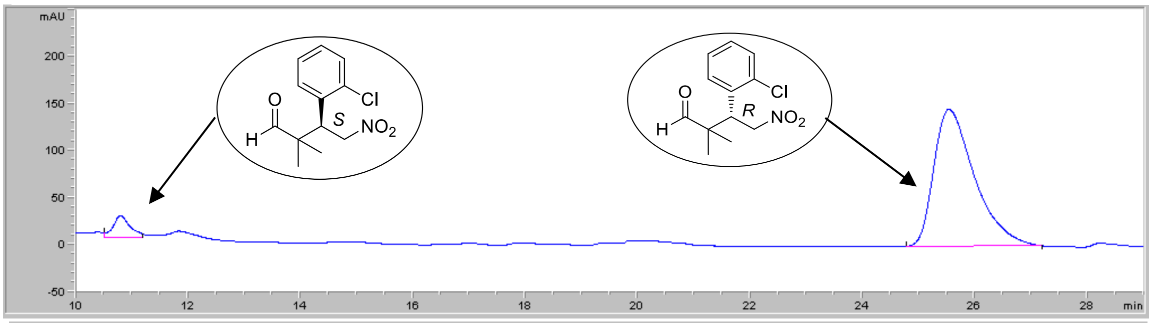

#	Time	Area	Height	Width	Area%	Symmetry
1	10.798	506.4	23.5	0.3598	6.402	0.797
2	25.536	7403.9	146.1	0.8444	93.598	0.584

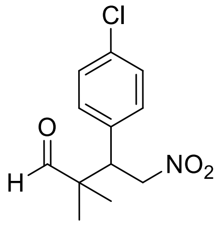

14ah

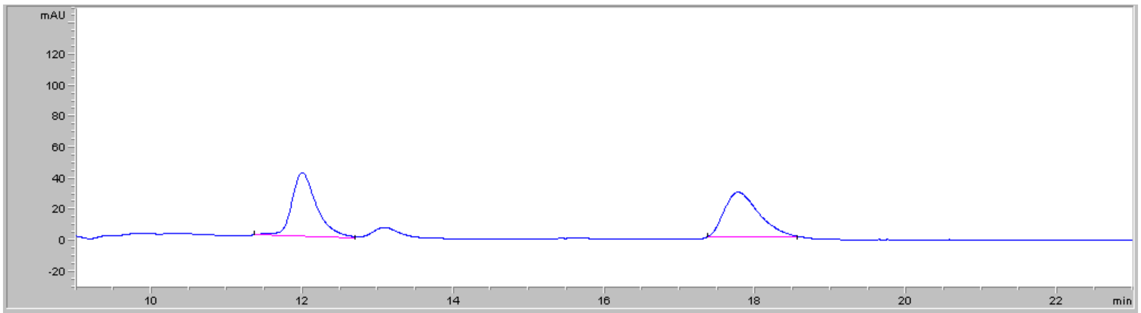

#	Time	Area	Height	Width	Area%	Symmetry
1	11.995	956	41.4	0.3849	49.932	0.702
2	17.767	958.6	29.4	0.5442	50.068	0.626

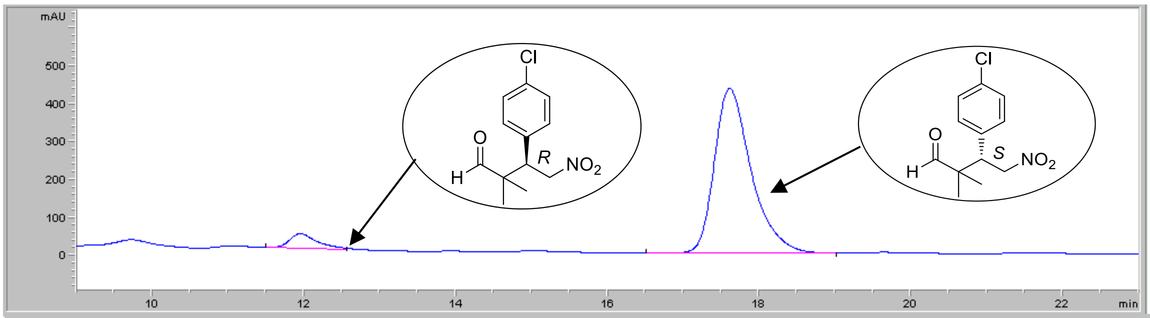

#	Time	Area	Height	Width	Area%	Symmetry
1	11.953	969.1	38.3	0.4216	6.162	0.599
2	17.607	14757.4	434.7	0.514	93.838	0.666

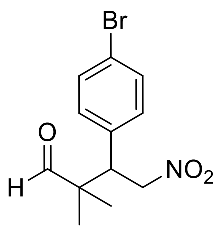

14ai

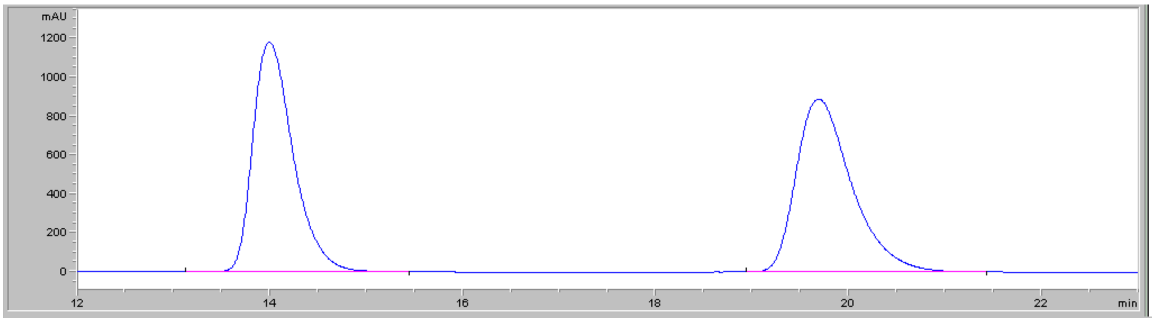

#	Time	Area	Height	Width	Area%	Symmetry
1	13.989	34349.9	1181.5	0.4512	49.166	0.654
2	19.685	35515.3	889.6	0.6137	50.834	0.64

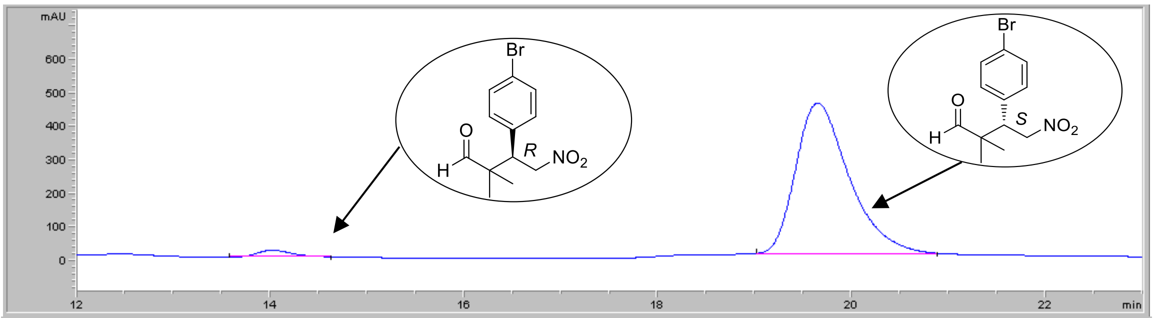

#	Time	Area	Height	Width	Area%	Symmetry
1	14.021	527.4	20.8	0.422	2.933	0.745
2	19.647	17457.2	450.8	0.6455	97.067	0.672

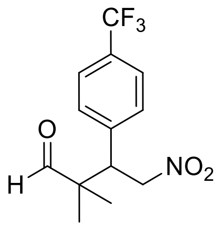

14aj

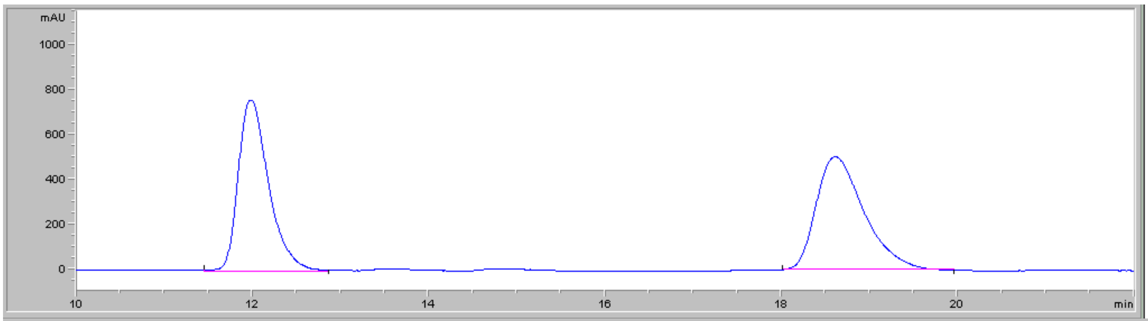

#	Time	Area	Height	Width	Area%	Symmetry
1	11.979	18440.9	760.7	0.3728	49.508	0.655
2	18.609	18807.2	500.5	0.6263	50.492	0.638

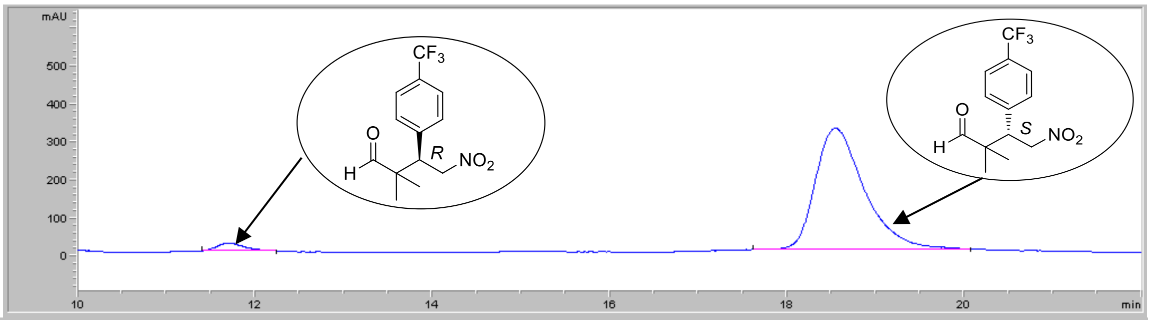

#	Time	Area	Height	Width	Area%	Symmetry
1	11.697	463.4	20.5	0.3763	3.680	0.701
2	18.544	12128.1	318.7	0.6343	96.320	0.664

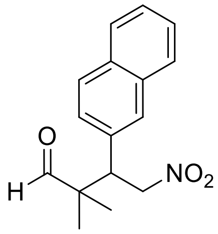

14ak

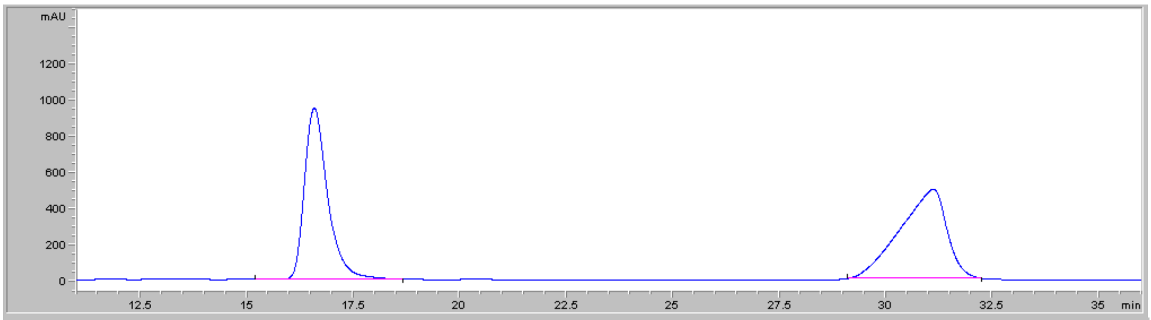

#	Time	Area	Height	Width	Area%	Symmetry
1	16.574	36039.7	941.8	0.5844	49.026	0.686
2	31.107	37471.2	490.7	1.2726	50.974	2.274

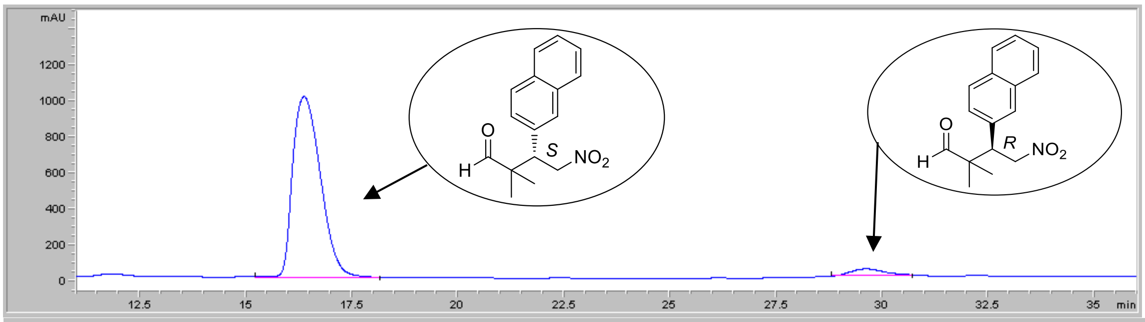

#	Time	Area	Height	Width	Area%	Symmetry
1	16.366	45907.9	1001.9	0.7637	95.480	0.672
2	29.615	2173.3	38.4	0.9445	4.520	0.78

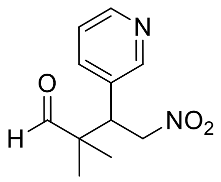

14al

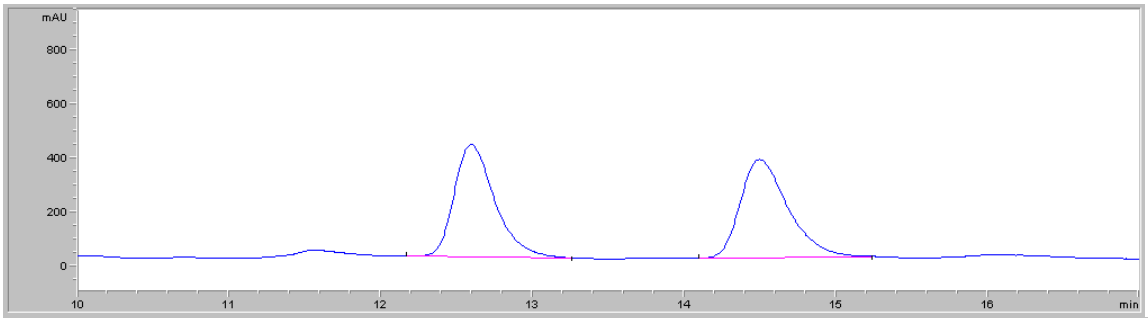

#	Time	Area	Height	Width	Area%	Symmetry
1	12.594	7765.4	417.2	0.3102	49.102	0.709
2	14.493	8049.6	365.9	0.3667	50.898	0.65

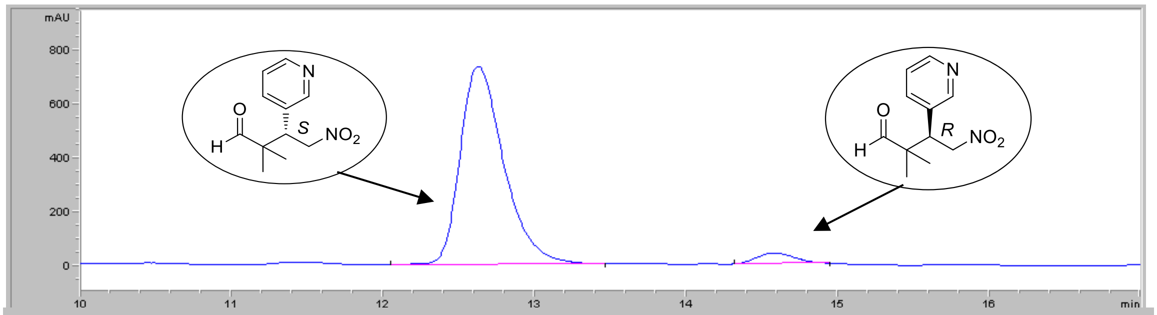

#	Time	Area	Height	Width	Area%	Symmetry
1	12.627	14775.6	735.1	0.335	95.599	0.701
2	14.582	680.2	39.4	0.2875	4.401	0.855

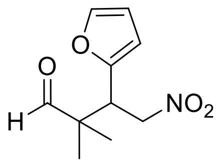

14am

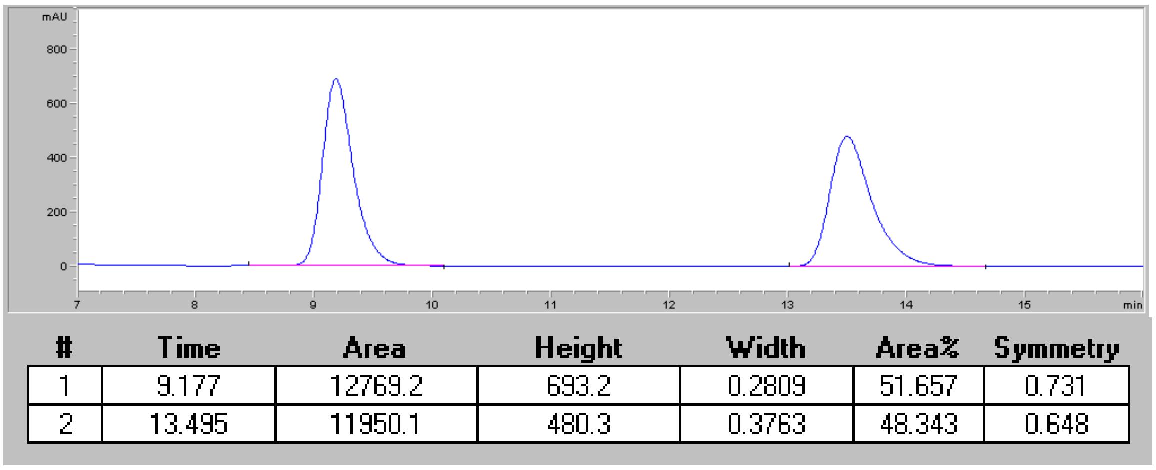

#	Time	Area	Height	Width	Area%	Symmetry
1	9.177	12769.2	693.2	0.2809	51.657	0.731
2	13.495	11950.1	480.3	0.3763	48.343	0.648

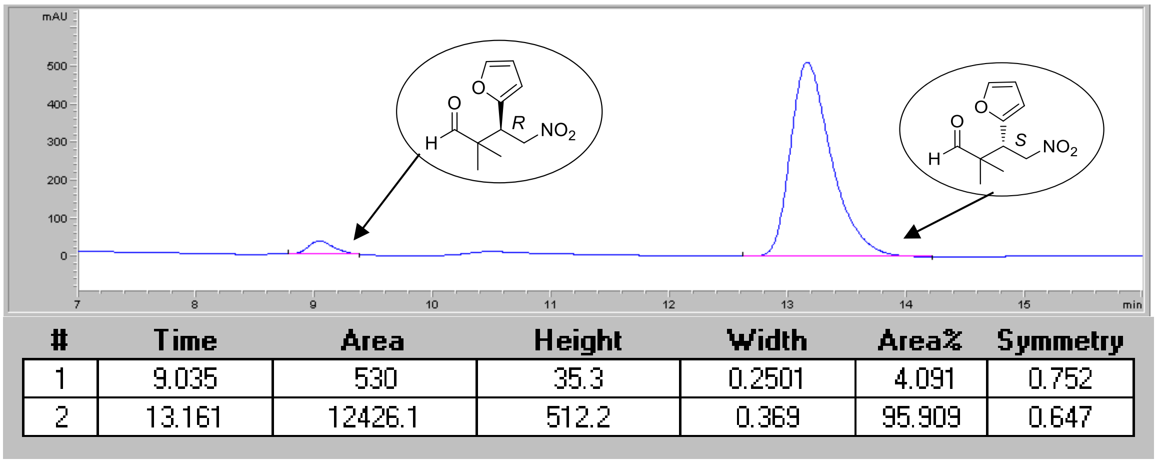

#	Time	Area	Height	Width	Area%	Symmetry
1	9.035	530	35.3	0.2501	4.091	0.752
2	13.161	12426.1	512.2	0.369	95.909	0.647

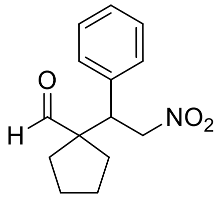

14ba

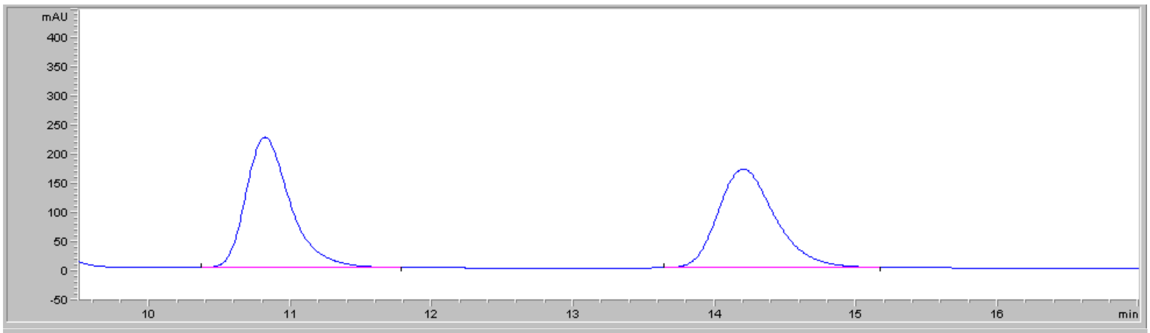

#	Time	Area	Height	Width	Area%	Symmetry
1	10.814	4911.1	224.7	0.335	50.775	0.7
2	14.198	4761.3	170.2	0.4304	49.225	0.747

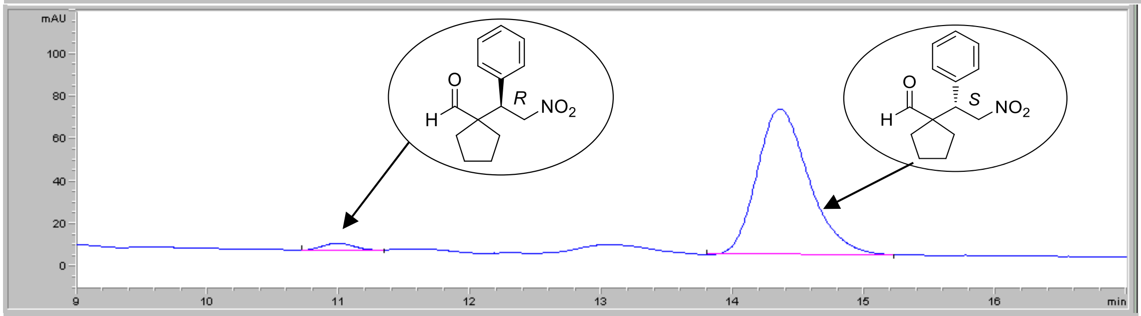

#	Time	Area	Height	Width	Area%	Symmetry
1	10.976	56.2	3.3	0.2812	2.892	0.802
2	14.356	1888.3	68.5	0.4195	97.108	0.761

Computational Methods

All reported structures were optimized at Density Functional Theory level by using the B3LYP functional as implemented in Gaussian 09. Optimizations were carried out with the 6-31G(d,p) basis set. The reported energy values correspond to Gibbs Free energies, including single point refinements at M06-2X/6-311+G(d,p) level of theory in a solvent model (IEFPCM, dichloromethane) on the previously optimized structures. The critical stationary points were characterized by frequency calculations in order to verify that they have the right number of imaginary frequencies, and the intrinsic reaction coordinates (IRC) were followed to verify the energy profiles connecting the key transition structures to the correct associated local minima.

Cartesian Coordinates of the computed structures:

13a

G at M06-2X/6-311+G** (IEFPCM, CH2Cl2) = -513,980183 Hartrees

Standard orientation:

Center Number	Atomic Number	Atomic Type	Coordinates (Angstroms) X	Y	Z
1	1	0	1.522514	-1.538099	0.000669
2	1	0	-4.677650	-0.379811	-0.000003
3	1	0	-3.138981	-2.331390	0.000421
4	1	0	-3.749962	1.926930	-0.000460
5	1	0	-1.298842	2.273250	-0.000498
6	1	0	-0.701712	-1.992260	0.000386
7	1	0	0.920817	1.483909	-0.000374
8	8	0	3.253046	1.174674	0.000737
9	8	0	3.833837	-0.934557	-0.000917
10	7	0	2.992496	-0.030920	0.000054
11	6	0	1.606458	-0.461125	0.000366
12	6	0	-2.736907	-1.322916	0.000231
13	6	0	-3.083060	1.070334	-0.000274
14	6	0	-1.704040	1.265180	-0.000294
15	6	0	-1.360730	-1.129951	0.000215
16	6	0	-3.603081	-0.224256	-0.000011
17	6	0	-0.819838	0.170969	-0.000039
18	6	0	0.614745	0.440596	-0.000040

Enamine of **12a** + catalyst **11**

G at M06-2X/6-311+G** (IEFPCM, CH_2Cl_2) = -921,769155 Hartrees

Standard orientation:

Center Number	Atomic Number	Atomic Type	Coordinates (Angstroms) X	Y	Z
1	6	0	-0.163696	3.441356	0.631502
2	6	0	3.826672	-0.266121	0.209409
3	6	0	1.537759	-1.349017	0.268465
4	7	0	0.142555	-1.306876	-0.175899
5	1	0	2.104025	-2.735544	-1.289673

6	1	0	5.495279	-1.648991	0.069909
7	1	0	1.021921	0.998548	0.960397
8	1	0	0.233515	4.765806	-1.852262
9	1	0	2.308337	-0.125423	-1.318503
10	1	0	3.870596	-0.206714	1.305051
11	1	0	1.505814	-1.322602	1.363031
12	1	0	1.386393	5.432328	-0.703770
13	1	0	3.711785	-2.839814	1.316595
14	1	0	-0.461777	2.476643	1.052934
15	1	0	-1.061898	3.884541	0.182453
16	1	0	1.591395	-3.506723	0.210043
17	1	0	4.491632	-1.589201	-1.372138
18	6	0	-0.867327	-0.780560	0.558620
19	6	0	1.143122	4.528576	-1.281793
20	6	0	0.941556	3.331114	-0.388998
21	7	0	1.768634	1.113218	0.286777
22	6	0	4.456998	-1.578086	-0.273294
23	6	0	2.349538	-0.124477	-0.215743
24	6	0	2.174534	-2.671232	-0.192839
25	6	0	3.647783	-2.783764	0.221452
26	6	0	1.747582	2.257434	-0.502923
27	1	0	1.955379	4.371233	-1.998923
28	1	0	4.383664	0.598828	-0.167398
29	1	0	2.521792	2.262200	-1.269196
30	1	0	4.068160	-3.719666	-0.163763
31	1	0	0.116630	4.092446	1.472208
32	1	0	-0.048891	-1.588495	-1.125324
33	6	0	-2.249677	-0.853768	0.028235
34	6	0	-2.608392	-1.633605	-1.088414
35	6	0	-3.260335	-0.094408	0.678727
36	6	0	-3.903550	-1.643260	-1.581896
37	1	0	-1.867270	-2.269743	-1.564464
38	6	0	-4.568163	-0.097664	0.162092
39	6	0	-4.881638	-0.858943	-0.952165
40	1	0	-4.157226	-2.256430	-2.440051
41	1	0	-5.314736	0.502347	0.671065
42	1	0	-5.899192	-0.855574	-1.332297
43	8	0	-0.626518	-0.233580	1.662939
44	8	0	-3.022485	0.638182	1.775519
45	1	0	-2.078218	0.457344	2.027642

Enamine of **12a** + catalyst **15**

G at M06-2X/6-311+G** (IEFPCM, CH_2Cl_2) = -846,537742 Hartrees

Standard orientation:

Center Number	Atomic Number	Atomic Type	Coordinates (Angstroms) X	Y	Z
1	6	0	-0.894114	3.617692	-0.486190
2	6	0	-3.415397	-1.195940	-0.135510
3	6	0	-0.904810	-1.449179	-0.193961
4	7	0	0.377928	-0.832514	0.143088
5	1	0	-0.934464	-2.744992	1.537146
6	1	0	-4.515771	-3.027133	0.232377
7	1	0	-0.966327	0.962722	-0.704991
8	1	0	-2.753973	4.832900	1.277762
9	1	0	-2.027171	-0.394795	1.307814

10	1	0	-3.477090	-1.284515	-1.227598
11	1	0	-0.899548	-1.551957	-1.283847
12	1	0	-3.412977	4.949621	-0.348889
13	1	0	-2.445563	-3.700591	-0.955535
14	1	0	-0.153258	2.812276	-0.534153
15	1	0	-0.484816	4.390888	0.176400
16	1	0	-0.214532	-3.468996	0.100590
17	1	0	-3.580371	-2.466166	1.611291
18	6	0	1.464336	-0.876288	-0.693980
19	6	0	-3.187027	4.239717	0.459909
20	6	0	-2.243635	3.155997	0.004548
21	7	0	-1.932557	0.761263	-0.476806
22	6	0	-3.558469	-2.576639	0.517837
23	6	0	-2.071734	-0.525064	0.207899
24	6	0	-1.043095	-2.842297	0.447050
25	6	0	-2.394133	-3.493907	0.122078
26	6	0	-2.634016	1.867141	0.010702
27	1	0	-4.139159	3.829609	0.810963
28	1	0	-4.241051	-0.544306	0.170977
29	1	0	-3.626484	1.623024	0.383851
30	1	0	-2.473529	-4.462048	0.629689
31	1	0	-0.951913	4.063362	-1.489088
32	1	0	0.497152	-0.478685	1.080961
33	6	0	2.755308	-0.331463	-0.142734
34	6	0	3.048171	-0.249453	1.225749
35	6	0	3.717838	0.085475	-1.071879
36	6	0	4.273474	0.259400	1.656197
37	1	0	2.342668	-0.613657	1.967741
38	6	0	4.937499	0.601358	-0.641892
39	1	0	3.484334	-0.008911	-2.126712
40	6	0	5.217169	0.692330	0.723741
41	1	0	4.493221	0.309858	2.718460
42	1	0	5.672511	0.929837	-1.370724
43	1	0	6.169763	1.091075	1.060044
44	8	0	1.389570	-1.302870	-1.843235

Enamine of **12a** + catalyst **16**

G at M06-2X/6-311+G** (IEFPCM, CH2Cl2) = -921,76483 Hartrees

Standard orientation:

Center Number	Atomic Number	Atomic Type	Coordinates (Angstroms) X	Y	Z
1	6	0	0.662917	3.580306	0.826685
2	6	0	3.944348	-0.692729	0.037759
3	6	0	1.536787	-1.444485	0.298748
4	7	0	0.129534	-1.219334	-0.033477
5	1	0	1.778620	-2.900571	-1.278403
6	1	0	5.388905	-2.292217	-0.228646
7	1	0	1.384209	0.934716	0.993187
8	1	0	1.049094	4.856445	-1.685598
9	1	0	2.334621	-0.335057	-1.357045
10	1	0	4.088565	-0.642119	1.125143
11	1	0	1.595815	-1.408459	1.392746
12	1	0	2.397584	5.299070	-0.647437
13	1	0	3.572322	-3.219967	1.184303
14	1	0	0.301084	2.675580	1.323702

15	1	0	-0.217865	4.118810	0.450751
16	1	0	1.292366	-3.588983	0.269433
17	1	0	4.280354	-2.096170	-1.579166
18	6	0	-0.733214	-0.518728	0.759830
19	6	0	1.952595	4.458500	-1.200623
20	6	0	1.632403	3.298393	-0.293585
21	7	0	2.113015	0.955159	0.288530
22	6	0	4.344112	-2.078775	-0.481601
23	6	0	2.469860	-0.345417	-0.261204
24	6	0	1.949199	-2.843111	-0.192131
25	6	0	3.422479	-3.159307	0.097757
26	6	0	2.225193	2.103050	-0.485389
27	1	0	2.657261	4.177539	-1.990287
28	1	0	4.582688	0.086954	-0.392762
29	1	0	2.918009	1.992108	-1.318956
30	1	0	3.674897	-4.145839	-0.307747
31	1	0	1.110316	4.222383	1.599521
32	1	0	-0.182071	-1.501970	-0.950725
33	6	0	-2.166649	-0.491819	0.332046
34	6	0	-2.742258	-1.443138	-0.520460
35	6	0	-2.981990	0.530354	0.843934
36	6	0	-4.087076	-1.366128	-0.873117
37	1	0	-2.155924	-2.278930	-0.891130
38	6	0	-4.321291	0.621763	0.495655
39	6	0	-4.879871	-0.327747	-0.370390
40	1	0	-4.524156	-2.117658	-1.526815
41	1	0	-4.953280	1.414446	0.881290
42	8	0	-0.354992	0.064858	1.782121
43	8	0	-6.199562	-0.193712	-0.679483
44	1	0	-6.463340	-0.905372	-1.277783
45	1	0	-2.536768	1.249432	1.522475

TS1

G at M06-2X/6-311+G** (IEFPCM, CH_2Cl_2) = -1435,73369 Hartrees

Standard orientation:

Center Number	Atomic Number	Atomic Type	Coordinates (Angstroms) X	Y	Z
1	6	0	-1.877841	-0.836883	-2.136535
2	6	0	2.156237	-3.648702	-0.550861
3	6	0	2.730666	-1.201709	-0.249790
4	7	0	2.215350	0.096748	0.173778
5	1	0	3.822301	-1.613504	1.567016
6	1	0	3.807436	-5.010558	-0.218278
7	1	0	0.430651	-0.927073	-1.112342
8	1	0	-4.076098	-2.392673	-1.274784
9	1	0	1.414284	-2.362565	1.024387
10	1	0	2.332351	-3.586187	-1.633026
11	1	0	2.954568	-1.112391	-1.318672
12	1	0	-2.987713	-3.417634	-2.209388
13	1	0	4.841410	-2.909075	-1.026599
14	1	0	-1.262176	0.021045	-1.844680
15	1	0	-2.864985	-0.444424	-2.386215
16	1	0	4.773946	-0.824560	0.308263
17	1	0	3.232369	-4.208353	1.238019
18	6	0	2.532577	1.237755	-0.495091
19	6	0	-3.104867	-2.893791	-1.252554

20	6	0	-1.981862	-1.888568	-1.038663
21	7	0	0.416152	-1.862554	-0.721377
22	6	0	3.450100	-4.051717	0.173144
23	6	0	1.655428	-2.286438	-0.045610
24	6	0	4.022474	-1.599610	0.487675
25	6	0	4.532277	-2.972476	0.025824
26	6	0	-0.750777	-2.472858	-0.614252
27	1	0	-3.127262	-3.648165	-0.459223
28	1	0	1.384784	-4.411206	-0.394928
29	1	0	-0.744341	-3.435835	-0.118206
30	1	0	5.425528	-3.250459	0.596021
31	1	0	-1.446790	-1.266984	-3.049011
32	6	0	-1.392988	-0.216326	1.203528
33	6	0	-2.515094	-0.875195	0.620325
34	1	0	1.704627	0.113654	1.062591
35	1	0	-1.181461	0.839935	1.141124
36	7	0	-0.495842	-0.952540	1.909419
37	8	0	-0.671571	-2.206532	2.018720
38	8	0	0.537321	-0.400941	2.427437
39	6	0	-3.686624	-0.039619	0.229245
40	6	0	-5.960519	1.500093	-0.416067
41	6	0	-4.983593	-0.546776	0.418586
42	6	0	-3.555299	1.261610	-0.288561
43	6	0	-4.678639	2.021388	-0.606721
44	6	0	-6.109033	0.213219	0.100873
45	1	0	-5.107741	-1.539633	0.841484
46	1	0	-2.568798	1.687046	-0.442491
47	1	0	-4.552491	3.024954	-1.002060
48	1	0	-7.100440	-0.198865	0.264281
49	1	0	-6.834521	2.095031	-0.663459
50	1	0	-2.791737	-1.775662	1.161532
51	6	0	2.030298	2.539047	0.016319
52	6	0	1.289753	2.669383	1.209273
53	6	0	2.311913	3.709042	-0.743079
54	6	0	0.832251	3.906812	1.640327
55	1	0	1.063628	1.795072	1.812613
56	6	0	1.840552	4.956067	-0.295419
57	6	0	1.111621	5.051045	0.879246
58	1	0	0.267751	3.984087	2.563773
59	1	0	2.070807	5.829060	-0.896892
60	1	0	0.757998	6.023315	1.211522
61	8	0	3.215420	1.179009	-1.545673
62	8	0	3.012991	3.680637	-1.885553
63	1	0	3.265128	2.724888	-2.021533

TS2

G at M06-2X/6-311+G** (IEFPCM, CH_2Cl_2) = -1435,72418 Hartrees

Standard orientation:

Center Number	Atomic Number	Atomic Type	Coordinates (Angstroms) X	Y	Z
1	1	0	4.144167	-1.710471	-1.612901
2	1	0	3.269984	-3.094533	-0.935099
3	1	0	3.029009	-2.629106	-2.623773
4	1	0	2.838285	0.373492	-2.310780
5	1	0	1.406101	-0.351282	-3.046710
6	1	0	1.253990	0.681671	-1.610323

7	1	0	-3.586810	-4.047293	-1.316823
8	1	0	-4.091522	-1.783061	1.745443
9	1	0	-4.320688	-1.801657	-0.002504
10	1	0	0.922388	-2.991378	-0.422510
11	1	0	-0.934679	-3.984711	0.215582
12	1	0	-2.908018	-5.364947	-0.369050
13	1	0	-0.443252	-0.590591	-1.429062
14	1	0	-4.679338	-4.093340	0.903416
15	1	0	-2.316636	-1.864561	-1.511829
16	1	0	-3.118635	-4.052165	1.713151
17	1	0	-1.625879	-1.936089	1.443028
18	1	0	-1.114225	-4.071437	-1.543381
19	6	0	3.186099	-2.236230	-1.610708
20	6	0	1.873960	-0.086912	-2.088177
21	6	0	2.050468	-1.308085	-1.203043
22	7	0	-2.211535	-0.138486	0.707802
23	7	0	-0.361834	-1.512415	-1.019601
24	6	0	0.858741	-1.995470	-0.841064
25	6	0	-2.738269	0.715213	-0.193081
26	6	0	-2.976549	-4.273285	-0.432102
27	6	0	-3.680454	-2.169272	0.806818
28	6	0	-3.658021	-3.705002	0.821249
29	6	0	-1.570654	-3.681269	-0.625702
30	6	0	-1.650203	-2.151695	-0.693397
31	6	0	-2.262946	-1.596703	0.620600
32	1	0	1.586196	1.210652	1.391742
33	1	0	7.250337	2.172565	-0.482705
34	1	0	5.048257	3.311622	-0.678382
35	1	0	7.341294	-0.213606	0.217166
36	1	0	5.253989	-1.440311	0.709483
37	1	0	2.969276	2.089897	-0.195607
38	1	0	2.912677	-1.536996	1.013775
39	8	0	0.742813	-1.847972	1.869087
40	8	0	-0.182049	0.024978	2.610424
41	7	0	0.726385	-0.580799	1.949834
42	6	0	1.688147	0.139880	1.310866
43	6	0	5.097009	2.268465	-0.380252
44	6	0	6.384732	0.291101	0.119224
45	6	0	5.205977	-0.403075	0.390584
46	6	0	3.920525	1.575533	-0.105826
47	6	0	6.334412	1.629465	-0.270236
48	6	0	3.952135	0.223705	0.284100
49	6	0	2.724741	-0.539376	0.629030
50	1	0	-1.649670	0.196542	1.491477
51	6	0	-2.566589	2.177479	0.021077
52	6	0	-1.777561	2.718071	1.056834
53	6	0	-3.223808	3.068945	-0.871250
54	6	0	-1.651026	4.089689	1.224158
55	1	0	-1.238575	2.061844	1.734388
56	6	0	-3.094485	4.456590	-0.685521
57	6	0	-2.320157	4.957311	0.349070
58	1	0	-1.038795	4.483669	2.028757
59	1	0	-3.614871	5.110359	-1.377405
60	1	0	-2.230109	6.032582	0.477051
61	8	0	-3.350052	0.282475	-1.206351
62	8	0	-3.974072	2.642641	-1.898637
63	1	0	-3.915813	1.647125	-1.877547

TS3

G at M06-2X/6-311+G** (IEFPCM, CH_2Cl_2) = -1435,71999 Hartrees

Standard orientation:

Center Number	Atomic Number	Atomic Type	Coordinates (Angstroms) X	Y	Z
1	1	0	2.966207	-1.121527	-2.422272
2	1	0	1.214614	-1.403085	-2.346408
3	1	0	1.879026	-0.198998	-3.457989
4	1	0	3.870066	1.171827	-1.761842
5	1	0	2.585631	2.231813	-2.357943
6	1	0	2.987785	2.179282	-0.632717
7	1	0	-2.301401	4.559164	1.552413
8	1	0	-4.393047	2.270475	-0.618811
9	1	0	-3.734448	2.474756	0.996745
10	1	0	-0.303436	0.203424	-1.418171
11	1	0	-0.733187	4.171832	-1.054878
12	1	0	-2.030602	5.774555	0.308156
13	1	0	1.060330	2.409703	-0.036294
14	1	0	-4.300469	4.686096	0.065425
15	1	0	-1.034182	2.164558	1.244586
16	1	0	-3.292182	4.399353	-1.347888
17	1	0	-2.185905	1.862282	-1.572577
18	1	0	-0.029923	4.310529	0.555727
19	6	0	1.986851	-0.636136	-2.457148
20	6	0	2.878346	1.567066	-1.535444
21	6	0	1.873212	0.431298	-1.379882
22	7	0	-2.538198	0.460149	-0.110741
23	7	0	0.257691	1.877991	-0.351910
24	6	0	0.529629	0.807305	-1.077021
25	6	0	-2.526615	-0.584299	-0.985039
26	6	0	-2.163580	4.699567	0.472061
27	6	0	-3.535760	2.668185	-0.066868
28	6	0	-3.392540	4.177162	-0.276581
29	6	0	-0.911314	3.953127	0.006819
30	6	0	-1.014120	2.420609	0.172907
31	6	0	-2.294345	1.842872	-0.483231
32	1	0	2.444400	0.906305	2.118090
33	1	0	7.769958	-1.264156	0.028399
34	1	0	6.892098	0.837601	1.027357
35	1	0	6.176974	-2.986918	-0.801872
36	1	0	3.742808	-2.606425	-0.633735
37	1	0	4.470814	1.224630	1.180848
38	1	0	1.887189	-1.393217	0.141711
39	8	0	-0.076853	-0.814637	1.130229
40	8	0	0.073472	0.663010	2.773195
41	7	0	0.606087	0.024702	1.829896
42	6	0	1.910838	0.211514	1.487799
43	6	0	6.205071	0.084381	0.652737
44	6	0	5.805985	-2.059639	-0.375362
45	6	0	4.431399	-1.840714	-0.288485
46	6	0	4.831924	0.301437	0.738796
47	6	0	6.699139	-1.096054	0.092628
48	6	0	3.916188	-0.656461	0.266556
49	6	0	2.440388	-0.487676	0.368425
50	1	0	-2.749561	0.246737	0.859112
51	6	0	-2.932673	-1.921401	-0.438507
52	6	0	-3.355752	-2.872930	-1.379568
53	6	0	-2.950310	-2.264327	0.933289

54	6	0	-3.830247	-4.118694	-0.986216
55	1	0	-3.310260	-2.593226	-2.426687
56	6	0	-3.436909	-3.517713	1.323109
57	6	0	-3.880397	-4.434592	0.374742
58	1	0	-4.163552	-4.835630	-1.730164
59	1	0	-3.441864	-3.749671	2.382982
60	1	0	-4.253499	-5.402707	0.697474
61	8	0	-2.221131	-0.435080	-2.175995
62	8	0	-2.536543	-1.410132	1.919646
63	1	0	-1.587432	-1.137887	1.750808

TS4

G at M06-2X/6-311+G** (IEFPCM, CH_2Cl_2) = -1435,71676 Hartrees

Standard orientation:

Center Number	Atomic Number	Atomic Type	Coordinates (Angstroms) X	Y	Z
1	1	0	3.722004	-2.672564	-1.070625
2	1	0	2.539627	-3.769232	-0.334792
3	1	0	2.588866	-3.557380	-2.090555
4	1	0	2.896895	-0.555757	-2.253646
5	1	0	1.471819	-1.199507	-3.076696
6	1	0	1.299932	0.092506	-1.881700
7	1	0	-4.319511	-3.545356	-1.372311
8	1	0	-4.690151	-0.668541	1.132505
9	1	0	-4.717325	-0.998812	-0.595815
10	1	0	0.209863	-3.203369	-0.168357
11	1	0	-1.875345	-3.622901	0.476718
12	1	0	-4.003521	-4.744290	-0.124663
13	1	0	-0.613521	-0.734029	-1.611371
14	1	0	-5.623793	-2.957524	0.646225
15	1	0	-2.610853	-1.678814	-1.772553
16	1	0	-4.178308	-3.030000	1.646737
17	1	0	-2.273553	-1.313595	1.224970
18	1	0	-1.888063	-4.019592	-1.247940
19	6	0	2.691345	-3.032741	-1.131418
20	6	0	1.848485	-0.826278	-2.114677
21	6	0	1.704570	-1.880043	-1.030859
22	7	0	-2.494975	0.422173	0.162012
23	7	0	-0.696435	-1.601692	-1.073912
24	6	0	0.371300	-2.285838	-0.719396
25	6	0	-1.963344	1.159897	-0.838273
26	6	0	-3.857210	-3.690586	-0.386387
27	6	0	-4.253282	-1.301842	0.352407
28	6	0	-4.542544	-2.780788	0.641210
29	6	0	-2.354015	-3.388465	-0.482084
30	6	0	-2.112501	-1.911949	-0.825582
31	6	0	-2.738307	-1.022120	0.275450
32	1	0	1.298902	1.002443	1.221551
33	1	0	7.140596	1.190507	-0.237952
34	1	0	5.089422	2.489351	-0.779430
35	1	0	6.929213	-1.084422	0.747860
36	1	0	4.694254	-2.041868	1.186037
37	1	0	2.863566	1.530559	-0.358884
38	1	0	2.336943	-1.882759	1.275936
39	8	0	0.075941	-1.876628	1.947011
40	8	0	-0.671677	0.135429	2.451223
41	7	0	0.206090	-0.616918	1.895521

42	6	0	1.270816	-0.075543	1.252902
43	6	0	5.007013	1.493279	-0.354562
44	6	0	6.040496	-0.512052	0.498735
45	6	0	4.778731	-1.055673	0.738612
46	6	0	3.747323	0.950590	-0.113202
47	6	0	6.159507	0.764347	-0.051004
48	6	0	3.608652	-0.336541	0.438539
49	6	0	2.284762	-0.934006	0.749713
50	1	0	-2.622094	0.917119	1.038036
51	6	0	-1.694782	2.607366	-0.531757
52	6	0	-1.539682	3.448655	-1.646721
53	6	0	-1.581194	3.170697	0.762883
54	6	0	-1.318740	4.812747	-1.507467
55	1	0	-1.607468	2.989596	-2.626888
56	6	0	-1.361676	4.548946	0.893826
57	6	0	-1.237741	5.364466	-0.225393
58	1	0	-1.214646	5.441466	-2.386179
59	1	0	-1.286408	4.949976	1.899088
60	1	0	-1.068355	6.429955	-0.097203
61	8	0	-1.698992	0.704769	-1.969171
62	8	0	-1.729800	2.466617	1.924431
63	1	0	-1.143407	1.661270	2.005624

TS5

G at M06-2X/6-311+G** (IEFPCM, CH_2Cl_2) = -1360,50237 Hartrees

Standard orientation:

Center Number	Atomic Number	Atomic Type	Coordinates (Angstroms) X	Y	Z
1	6	0	-1.798572	-0.952100	-2.108708
2	6	0	2.441473	-3.413011	-0.450923
3	6	0	2.859903	-0.917392	-0.357851
4	7	0	2.271711	0.373334	-0.019532
5	1	0	4.033058	-1.109475	1.444721
6	1	0	4.187632	-4.634352	-0.065740
7	1	0	0.531041	-0.835655	-1.135086
8	1	0	-3.872838	-2.598799	-1.111083
9	1	0	1.659085	-2.057183	1.043341
10	1	0	2.583020	-3.425724	-1.539896
11	1	0	3.046947	-0.895274	-1.437803
12	1	0	-2.741603	-3.597095	-2.023487
13	1	0	5.058586	-2.543439	-1.072017
14	1	0	-1.240646	-0.039210	-1.872071
15	1	0	-2.814762	-0.644285	-2.360997
16	1	0	4.888235	-0.366224	0.091387
17	1	0	3.601349	-3.753943	1.340011
18	6	0	2.574783	1.491327	-0.752092
19	6	0	-2.870319	-3.034155	-1.090556
20	6	0	-1.811442	-1.949124	-0.956646
21	7	0	0.585519	-1.752041	-0.702865
22	6	0	3.779255	-3.671062	0.259544
23	6	0	1.865838	-2.048947	-0.036765
24	6	0	4.197187	-1.170719	0.360784
25	6	0	4.784017	-2.541190	-0.008113
26	6	0	-0.536207	-2.426916	-0.530115
27	1	0	-2.824943	-3.747925	-0.261306
28	1	0	1.728275	-4.209561	-0.209864
29	1	0	-0.456831	-3.355767	0.022029

30	1	0	5.709186	-2.716579	0.552093
31	1	0	-1.350184	-1.397415	-3.005471
32	6	0	-1.292541	-0.102001	1.163932
33	6	0	-2.374800	-0.880308	0.661161
34	1	0	1.807809	0.432865	0.890387
35	1	0	-1.163307	0.959411	1.020981
36	7	0	-0.323121	-0.715964	1.893149
37	8	0	-0.398809	-1.968221	2.095701
38	8	0	0.672183	-0.049919	2.341869
39	6	0	-3.618856	-0.161971	0.260367
40	6	0	-6.024478	1.160210	-0.392781
41	6	0	-4.866520	-0.751458	0.526669
42	6	0	-3.604927	1.110281	-0.339810
43	6	0	-4.793042	1.762290	-0.661844
44	6	0	-6.056798	-0.099085	0.205736
45	1	0	-4.900411	-1.722868	1.011593
46	1	0	-2.659648	1.598044	-0.555655
47	1	0	-4.757195	2.745660	-1.121418
48	1	0	-7.007758	-0.573150	0.430167
49	1	0	-6.949018	1.671729	-0.643032
50	1	0	-2.564302	-1.763480	1.264846
51	6	0	2.030038	2.808632	-0.262977
52	6	0	1.281799	2.968880	0.914105
53	6	0	2.310543	3.931391	-1.054393
54	6	0	0.825910	4.235659	1.282684
55	1	0	1.046115	2.123650	1.554603
56	6	0	1.852556	5.192618	-0.683161
57	1	0	2.893633	3.783520	-1.956578
58	6	0	1.108149	5.348065	0.488380
59	1	0	0.251304	4.351195	2.197138
60	1	0	2.077639	6.054650	-1.304569
61	1	0	0.752014	6.331456	0.782278
62	8	0	3.243136	1.425445	-1.785636

TS6

G at M06-2X/6-311+G** (IEFPCM, CH_2Cl_2) = -1360,4925 Hartrees

Standard orientation:

Center Number	Atomic Number	Atomic Type	Coordinates (Angstroms) X	Y	Z
1	1	0	3.526565	-2.495183	-1.407451
2	1	0	2.357696	-3.631856	-0.712967
3	1	0	2.302804	-3.239641	-2.436097
4	1	0	2.694003	-0.244687	-2.307133
5	1	0	1.184190	-0.729307	-3.084118
6	1	0	1.166010	0.409531	-1.724576
7	1	0	-4.535811	-3.185182	-1.328602
8	1	0	-4.641132	-0.568018	1.496000
9	1	0	-4.777447	-0.692391	-0.256823
10	1	0	0.069238	-3.015387	-0.306033
11	1	0	-1.997766	-3.557180	0.349374
12	1	0	-4.216454	-4.514142	-0.220891
13	1	0	-0.786931	-0.504036	-1.602849
14	1	0	-5.712168	-2.750868	0.798414
15	1	0	-2.838193	-1.357283	-1.611024
16	1	0	-4.229560	-2.986889	1.714295
17	1	0	-2.268668	-1.355494	1.367853

18	1	0	-2.132789	-3.765596	-1.403489
19	6	0	2.483332	-2.819949	-1.437717
20	6	0	1.648476	-0.493672	-2.116316
21	6	0	1.538389	-1.661376	-1.153129
22	7	0	-2.372482	0.489033	0.525027
23	7	0	-0.870324	-1.375738	-1.089624
24	6	0	0.219473	-2.071570	-0.814215
25	6	0	-2.307799	1.326748	-0.536802
26	6	0	-4.031945	-3.447211	-0.388397
27	6	0	-4.283149	-1.123786	0.623136
28	6	0	-4.625387	-2.615754	0.759235
29	6	0	-2.521122	-3.201526	-0.546957
30	6	0	-2.262630	-1.704225	-0.750003
31	6	0	-2.761988	-0.920683	0.492436
32	1	0	1.460245	1.020809	1.352801
33	1	0	7.239766	0.870840	-0.327542
34	1	0	5.286238	2.372403	-0.667889
35	1	0	6.873542	-1.459889	0.464207
36	1	0	4.583151	-2.272131	0.906769
37	1	0	3.004302	1.561779	-0.236013
38	1	0	2.251238	-1.936471	1.109927
39	8	0	0.032503	-1.796795	1.919441
40	8	0	-0.501773	0.240309	2.604246
41	7	0	0.267722	-0.549666	1.964109
42	6	0	1.354003	-0.051552	1.310100
43	6	0	5.135819	1.351780	-0.328335
44	6	0	6.027677	-0.797949	0.303454
45	6	0	4.733879	-1.258555	0.546541
46	6	0	3.844333	0.891991	-0.082480
47	6	0	6.233708	0.509166	-0.137424
48	6	0	3.617724	-0.424576	0.360032
49	6	0	2.260284	-0.938480	0.682060
50	1	0	-1.923968	0.750677	1.400677
51	6	0	-1.822019	2.729270	-0.272974
52	6	0	-1.106885	3.109980	0.873426
53	6	0	-2.090269	3.684761	-1.262922
54	6	0	-0.683226	4.431133	1.023099
55	1	0	-0.850428	2.385202	1.641571
56	6	0	-1.673605	5.003999	-1.104467
57	1	0	-2.626818	3.365331	-2.149492
58	6	0	-0.969208	5.380984	0.041118
59	1	0	-0.125918	4.716506	1.910636
60	1	0	-1.896164	5.737762	-1.873825
61	1	0	-0.641267	6.409067	0.166179
62	8	0	-2.620618	0.979315	-1.687870

TS7

G at M06-2X/6-311+G** (IEFPCM, CH_2Cl_2) = -1435,72874 Hartrees

Standard orientation:

Center Number	Atomic Number	Atomic Type	Coordinates (Angstroms) X	Y	Z
1	6	0	-1.917708	-1.127713	-2.093820
2	6	0	2.133805	-3.826577	-0.368452
3	6	0	2.747322	-1.368568	-0.357203
4	7	0	2.263358	-0.027933	-0.054213
5	1	0	3.924984	-1.601983	1.438372
6	1	0	3.784087	-5.169937	0.033414

7	1	0	0.424881	-1.135499	-1.125274
8	1	0	-4.099105	-2.573910	-1.016157
9	1	0	1.476200	-2.367425	1.089453
10	1	0	2.259502	-3.883061	-1.457953
11	1	0	2.922769	-1.393188	-1.439141
12	1	0	-3.050802	-3.691081	-1.888487
13	1	0	4.800020	-3.183563	-1.046327
14	1	0	-1.288457	-0.251648	-1.901663
15	1	0	-2.909854	-0.753380	-2.351813
16	1	0	4.818335	-0.967015	0.054941
17	1	0	3.288386	-4.205572	1.418743
18	6	0	2.635262	1.042283	-0.829238
19	6	0	-3.130621	-3.079741	-0.980815
20	6	0	-1.994377	-2.070600	-0.899901
21	7	0	0.411914	-2.036202	-0.658148
22	6	0	3.456744	-4.168472	0.334168
23	6	0	1.672045	-2.409437	0.007962
24	6	0	4.069809	-1.706668	0.354913
25	6	0	4.542389	-3.128937	0.020389
26	6	0	-0.755129	-2.620149	-0.456792
27	1	0	-3.130740	-3.757905	-0.121033
28	1	0	1.363663	-4.557038	-0.095444
29	1	0	-0.740296	-3.529230	0.132602
30	1	0	5.459377	-3.359773	0.573948
31	1	0	-1.514472	-1.644547	-2.973506
32	6	0	-1.326711	-0.181057	1.147719
33	6	0	-2.467566	-0.892143	0.677840
34	1	0	1.818181	0.095772	0.857741
35	1	0	-1.116012	0.860395	0.961165
36	7	0	-0.404509	-0.837982	1.901443
37	8	0	-0.573370	-2.072019	2.151445
38	8	0	0.637107	-0.230613	2.327057
39	6	0	-3.653402	-0.098157	0.246186
40	6	0	-5.951506	1.375270	-0.468149
41	6	0	-4.942196	-0.577324	0.536960
42	6	0	-3.542991	1.141421	-0.409651
43	6	0	-4.677932	1.868169	-0.762105
44	6	0	-6.079322	0.149971	0.185657
45	1	0	-5.049837	-1.520890	1.064284
46	1	0	-2.563281	1.544039	-0.646130
47	1	0	-4.567407	2.823922	-1.265929
48	1	0	-7.063508	-0.239157	0.429388
49	1	0	-6.834540	1.944521	-0.742463
50	1	0	-2.722491	-1.733999	1.315398
51	6	0	2.193903	2.405001	-0.381764
52	6	0	1.467393	2.663911	0.790642
53	6	0	2.544905	3.485944	-1.206921
54	6	0	1.103819	3.967380	1.122671
55	1	0	1.170765	1.863804	1.463174
56	6	0	2.186092	4.786663	-0.884324
57	6	0	1.461454	5.032499	0.288613
58	1	0	0.543969	4.156526	2.036387
59	1	0	2.457500	5.623358	-1.519348
60	8	0	3.282615	0.890040	-1.867934
61	8	0	1.132072	6.326804	0.566107
62	1	0	0.653517	6.358407	1.405207
63	1	0	3.109053	3.272374	-2.108034

TS8

G at M06-2X/6-311+G** (IEFPCM, CH_2Cl_2) = -1435,71829 Hartrees

Standard orientation:

Center Number	Atomic Number	Atomic Type	Coordinates (Angstroms) X	Y	Z
1	1	0	3.629449	-2.570994	-1.424303
2	1	0	2.514264	-3.757607	-0.724965
3	1	0	2.431626	-3.364497	-2.446490
4	1	0	2.702422	-0.352672	-2.303746
5	1	0	1.209640	-0.890073	-3.078543
6	1	0	1.151436	0.235019	-1.708880
7	1	0	-4.398153	-3.597690	-1.304490
8	1	0	-4.608319	-0.953618	1.491329
9	1	0	-4.736162	-1.100597	-0.260342
10	1	0	0.201203	-3.237398	-0.305499
11	1	0	-1.847036	-3.850950	0.376505
12	1	0	-4.027667	-4.898999	-0.180444
13	1	0	-0.765378	-0.764473	-1.600408
14	1	0	-5.593295	-3.183752	0.814969
15	1	0	-2.775763	-1.705593	-1.604211
16	1	0	-4.104502	-3.351596	1.735910
17	1	0	-2.208509	-1.656409	1.374014
18	1	0	-1.975588	-4.082654	-1.374075
19	6	0	2.600612	-2.939276	-1.448356
20	6	0	1.668419	-0.644541	-2.110481
21	6	0	1.609069	-1.822625	-1.155962
22	7	0	-2.375807	0.176952	0.516989
23	7	0	-0.809118	-1.638718	-1.086082
24	6	0	0.309913	-2.287208	-0.812976
25	6	0	-2.330025	1.007690	-0.553553
26	6	0	-3.885007	-3.827724	-0.360936
27	6	0	-4.227569	-1.504029	0.624684
28	6	0	-4.512510	-3.006702	0.776107
29	6	0	-2.384750	-3.525247	-0.522807
30	6	0	-2.185843	-2.021065	-0.740887
31	6	0	-2.714906	-1.245275	0.494560
32	1	0	1.447645	0.859189	1.349816
33	1	0	7.232108	0.874980	-0.330329
34	1	0	5.234310	2.312381	-0.689690
35	1	0	6.935813	-1.457601	0.484734
36	1	0	4.670055	-2.335480	0.930579
37	1	0	2.977023	1.436349	-0.252965
38	1	0	2.327829	-2.074368	1.115058
39	8	0	0.107696	-1.998905	1.927957
40	8	0	-0.491325	0.023946	2.601055
41	7	0	0.303229	-0.745301	1.966190
42	6	0	1.375415	-0.216232	1.312019
43	6	0	5.114465	1.291438	-0.339061
44	6	0	6.070417	-0.823395	0.316325
45	6	0	4.790833	-1.320991	0.561699
46	6	0	3.837165	0.794957	-0.090309
47	6	0	6.237237	0.484828	-0.137683
48	6	0	3.650090	-0.523397	0.365002
49	6	0	2.308972	-1.076113	0.688445
50	1	0	-1.935793	0.459057	1.390079
51	6	0	-1.880671	2.418803	-0.305020
52	6	0	-1.204502	2.842486	0.849314
53	6	0	-2.137959	3.358029	-1.316632

54	6	0	-0.811987	4.171626	0.989846
55	1	0	-0.945763	2.142432	1.639195
56	6	0	-1.758334	4.685832	-1.180862
57	6	0	-1.093747	5.099096	-0.019559
58	1	0	-0.282068	4.487761	1.886085
59	1	0	-1.964861	5.416201	-1.955977
60	8	0	-2.632687	0.637209	-1.701257
61	8	0	-0.739087	6.413233	0.068946
62	1	0	-0.305449	6.567686	0.918753
63	1	0	-2.644225	3.015789	-2.212463

17

G at M06-2X/6-311+G** (IEFPCM, CH_2Cl_2) = -1435,76312 Hartrees

Standard orientation:

Center Number	Atomic Number	Atomic Type	Coordinates (Angstroms) X	Y	Z
1	6	0	-0.990268	-1.469542	-2.173873
2	6	0	4.132140	-1.820622	-0.648778
3	6	0	3.233308	0.500694	-0.218814
4	7	0	2.102058	1.321864	0.213851
5	1	0	4.289274	0.624862	1.665186
6	1	0	6.251751	-2.058840	-0.238161
7	1	0	1.432270	-0.620900	-1.557913
8	1	0	-1.702279	-4.103881	-1.777139
9	1	0	2.745898	-1.268421	0.898077
10	1	0	4.290732	-1.596436	-1.712412
11	1	0	3.409161	0.767925	-1.265538
12	1	0	0.025658	-4.044968	-2.156865
13	1	0	5.967080	0.313730	-0.884314
14	1	0	-0.953981	-0.399422	-1.946588
15	1	0	-1.994633	-1.682713	-2.551117
16	1	0	4.695164	1.905516	0.525540
17	1	0	5.262480	-1.824508	1.195769
18	6	0	1.242900	1.886692	-0.671131
19	6	0	-0.716703	-3.837931	-1.378351
20	6	0	-0.675190	-2.366864	-0.970670
21	7	0	1.702846	-1.359753	-0.916669
22	6	0	5.398458	-1.495240	0.155850
23	6	0	2.914766	-1.014499	-0.158584
24	6	0	4.489237	0.832557	0.603967
25	6	0	5.699430	0.009992	0.137085
26	6	0	0.607525	-1.918154	-0.198485
27	1	0	-0.507390	-4.494932	-0.527510
28	1	0	3.893889	-2.888307	-0.590233
29	1	0	0.967329	-2.758373	0.407488
30	1	0	6.567290	0.236548	0.766641
31	1	0	-0.278373	-1.661777	-2.983076
32	6	0	-0.351294	-1.069710	0.749084
33	6	0	-1.503141	-1.990483	0.328069
34	1	0	1.789559	1.204056	1.171448
35	1	0	-0.438389	-0.032230	0.439403
36	7	0	0.046843	-1.045199	2.180978
37	8	0	-0.204326	-2.021485	2.880446
38	8	0	0.690723	-0.059979	2.576981
39	6	0	-2.921158	-1.486115	0.227965
40	6	0	-5.605286	-0.658839	-0.011845
41	6	0	-3.971827	-2.415412	0.283932

42	6	0	-3.243070	-0.133163	0.055647
43	6	0	-4.572134	0.276629	-0.061040
44	6	0	-5.299799	-2.009922	0.161556
45	1	0	-3.742898	-3.467764	0.432964
46	1	0	-2.462083	0.619499	0.018107
47	1	0	-4.794292	1.331870	-0.189455
48	1	0	-6.095536	-2.747722	0.210387
49	1	0	-6.639068	-0.338562	-0.101870
50	1	0	-1.480310	-2.859830	0.991744
51	6	0	0.103923	2.683990	-0.150672
52	6	0	-0.012366	3.072524	1.199077
53	6	0	-0.918646	3.071005	-1.061663
54	6	0	-1.109812	3.787713	1.653575
55	1	0	0.776862	2.823820	1.900322
56	6	0	-2.034083	3.784037	-0.587842
57	6	0	-2.125435	4.134774	0.750842
58	1	0	-1.177167	4.077661	2.696527
59	1	0	-2.800660	4.060127	-1.303981
60	1	0	-2.989326	4.694453	1.098014
61	8	0	1.395381	1.719745	-1.907314
62	8	0	-0.871413	2.773708	-2.368801
63	1	0	0.018008	2.350575	-2.518458

18

G at M06-2X/6-311+G** (IEFPCM, CH_2Cl_2) = -1435,75898 Hartrees

Standard orientation:

Center Number	Atomic Number	Atomic Type	Coordinates (Angstroms) X	Y	Z
1	6	0	-2.089808	-1.263253	-1.827992
2	6	0	1.931450	-3.792779	-0.527008
3	6	0	2.630463	-1.373589	-0.365036
4	7	0	2.252838	-0.046156	0.130476
5	1	0	3.998365	-1.809873	1.249008
6	1	0	3.560798	-5.221894	-0.434646
7	1	0	0.253973	-0.963549	-0.690112
8	1	0	-4.267972	-2.604749	-0.743029
9	1	0	1.504328	-2.452777	1.110763
10	1	0	1.931939	-3.748870	-1.623807
11	1	0	2.675758	-1.290797	-1.456402
12	1	0	-2.972624	-3.733045	-1.160071
13	1	0	4.518047	-3.173764	-1.452304
14	1	0	-1.465557	-0.365994	-1.867410
15	1	0	-3.063676	-0.997860	-2.245686
16	1	0	4.751772	-1.070741	-0.165621
17	1	0	3.264693	-4.372403	1.076428
18	6	0	2.655374	1.084855	-0.509572
19	6	0	-3.281610	-2.968090	-0.439952
20	6	0	-2.260127	-1.815959	-0.402173
21	7	0	0.246969	-1.964469	-0.521652
22	6	0	3.306967	-4.242285	-0.013864
23	6	0	1.549618	-2.404286	0.012547
24	6	0	4.012820	-1.813237	0.150504
25	6	0	4.389205	-3.210726	-0.361807
26	6	0	-0.912780	-2.379765	0.168822
27	1	0	-3.378055	-3.445250	0.541409
28	1	0	1.164186	-4.522907	-0.242313
29	1	0	-0.942398	-3.470602	0.227530

30	1	0	5.356325	-3.513354	0.055453
31	1	0	-1.626655	-2.013805	-2.472258
32	6	0	-1.653870	0.022078	1.272393
33	6	0	-2.791191	-0.711146	0.622492
34	1	0	1.817007	0.001018	1.048337
35	1	0	-1.576614	1.096399	1.370372
36	7	0	-0.703837	-0.679143	1.821363
37	8	0	-0.849509	-2.075630	1.648110
38	8	0	0.314057	-0.310060	2.455997
39	6	0	-3.840748	0.237228	0.066869
40	6	0	-5.832485	1.957365	-0.941718
41	6	0	-5.200686	-0.045427	0.255903
42	6	0	-3.497523	1.405833	-0.629506
43	6	0	-4.482631	2.257908	-1.128620
44	6	0	-6.188887	0.801809	-0.246476
45	1	0	-5.488620	-0.932779	0.813379
46	1	0	-2.452564	1.657393	-0.786670
47	1	0	-4.192932	3.158005	-1.662650
48	1	0	-7.235874	0.562688	-0.084949
49	1	0	-6.599108	2.621741	-1.328859
50	1	0	-3.268649	-1.293298	1.422169
51	6	0	2.292147	2.412533	0.051625
52	6	0	1.536202	2.579063	1.229239
53	6	0	2.731590	3.573747	-0.644325
54	6	0	1.224013	3.841235	1.714544
55	1	0	1.178492	1.713993	1.779228
56	6	0	2.410255	4.847209	-0.140700
57	6	0	1.668005	4.976888	1.021924
58	1	0	0.644950	3.944087	2.626571
59	1	0	2.762615	5.712061	-0.692721
60	1	0	1.430283	5.968690	1.396759
61	8	0	3.308270	0.996030	-1.575449
62	8	0	3.445948	3.513096	-1.776137
63	1	0	3.561277	2.539869	-1.968072

TS9

G at M06-2X/6-311+G** (IEFPCM, CH_2Cl_2) = -1435,71841 Hartrees

Standard orientation:

Center Number	Atomic Number	Atomic Type	Coordinates (Angstroms) X	Y	Z
1	6	0	-1.132242	-0.229424	-1.810810
2	6	0	3.125189	-2.695355	-0.553319
3	6	0	3.453326	-0.174933	-0.337296
4	7	0	2.849481	1.046099	0.177953
5	1	0	4.870879	-0.445240	1.272608
6	1	0	4.967488	-3.827779	-0.523366
7	1	0	0.906874	-0.120142	-0.267903
8	1	0	-2.708821	-2.449813	-2.264941
9	1	0	2.507677	-1.482003	1.115582
10	1	0	3.099945	-2.644230	-1.650100
11	1	0	3.485207	-0.059227	-1.427140
12	1	0	-1.057885	-2.778532	-2.802818
13	1	0	5.581450	-1.620914	-1.475388
14	1	0	-0.875248	0.637549	-1.200229
15	1	0	-2.107310	-0.035472	-2.260020
16	1	0	5.494373	0.451423	-0.108724

17	1	0	4.557825	-3.086933	1.016646
18	6	0	2.430166	2.063343	-0.658265
19	6	0	-1.696866	-2.672647	-1.919441
20	6	0	-1.190019	-1.540086	-0.997593
21	7	0	1.175850	-1.103602	-0.422284
22	6	0	4.568291	-2.915168	-0.067944
23	6	0	2.558657	-1.392272	0.020458
24	6	0	4.882817	-0.410367	0.174450
25	6	0	5.468965	-1.713670	-0.386456
26	6	0	0.203444	-1.951867	-0.554515
27	1	0	-1.719534	-3.634295	-1.395661
28	1	0	2.507834	-3.547417	-0.249118
29	1	0	0.456460	-3.005180	-0.568705
30	1	0	6.473822	-1.879247	0.017001
31	1	0	-0.397933	-0.307084	-2.618397
32	6	0	-1.559954	-0.689361	1.469792
33	6	0	-2.159144	-1.430782	0.275015
34	1	0	2.342236	0.966988	1.051331
35	6	0	-3.529372	-0.870741	-0.084690
36	6	0	-6.103134	0.104333	-0.708827
37	6	0	-4.595898	-1.747541	-0.332095
38	6	0	-3.783845	0.509202	-0.144492
39	6	0	-5.055515	0.991001	-0.454529
40	6	0	-5.869318	-1.268889	-0.643955
41	1	0	-4.429422	-2.819441	-0.265418
42	1	0	-2.985382	1.216597	0.056655
43	1	0	-5.227545	2.062733	-0.493726
44	1	0	-6.678188	-1.970282	-0.827137
45	1	0	-7.093283	0.481015	-0.947534
46	1	0	-2.308348	-2.474742	0.577038
47	6	0	1.323678	2.928269	-0.126978
48	6	0	1.282954	4.269263	-0.527834
49	6	0	0.268802	2.408753	0.670920
50	6	0	0.250226	5.111524	-0.127954
51	1	0	2.081797	4.629139	-1.168611
52	6	0	-0.773438	3.264339	1.066257
53	6	0	-0.776036	4.600112	0.672337
54	1	0	0.240509	6.151858	-0.436588
55	1	0	-1.565697	2.868098	1.694223
56	1	0	-1.587660	5.247183	0.993951
57	8	0	2.924248	2.236556	-1.769362
58	8	0	0.293391	1.104371	1.016884
59	1	0	-0.646237	0.470742	1.211746
60	7	0	-0.693684	-1.505333	2.233329
61	8	0	-0.428389	-1.233098	3.406351
62	8	0	-0.135749	-2.495115	1.646636
63	1	0	-2.285811	-0.239431	2.144264
